# Effects of *Tcte1* knockout on energy chain transportation and spermatogenesis: implications for male infertility

**DOI:** 10.1093/hropen/hoae020

**Published:** 2024-04-04

**Authors:** Marta Olszewska, Agnieszka Malcher, Tomasz Stokowy, Nijole Pollock, Andrea J Berman, Sylwia Budkiewicz, Marzena Kamieniczna, Hanna Jackowiak, Joanna Suszynska-Zajczyk, Piotr Jedrzejczak, Alexander N Yatsenko, Maciej Kurpisz

**Affiliations:** Institute of Human Genetics, Polish Academy of Sciences, Poznan, Poland; Institute of Human Genetics, Polish Academy of Sciences, Poznan, Poland; Scientific Computing Group, IT Division, University of Bergen, Bergen, Norway; Department of OB/GYN and Reproductive Sciences, School of Medicine, University of Pittsburgh, Pittsburgh, PA, USA; Department of Biological Sciences, University of Pittsburgh, Pittsburgh, PA, USA; Institute of Human Genetics, Polish Academy of Sciences, Poznan, Poland; Institute of Human Genetics, Polish Academy of Sciences, Poznan, Poland; Department of Histology and Embryology, Poznan University of Life Sciences, Poznan, Poland; Department of Biochemistry and Biotechnology, University of Life Sciences, Poznan, Poland; Division of Infertility and Reproductive Endocrinology, Department of Gynecology, Obstetrics and Gynecological Oncology, Poznan University of Medical Sciences, Poznan, Poland; Department of OB/GYN and Reproductive Sciences, School of Medicine, University of Pittsburgh, Pittsburgh, PA, USA; Institute of Human Genetics, Polish Academy of Sciences, Poznan, Poland

**Keywords:** *TCTE1*, N-DRC, male infertility, oligoasthenoteratozoospermia, sperm motility, sperm mitochondria, *mt-Co2*, *Fetub*, haploinsufficiency, MMAF

## Abstract

**STUDY QUESTION:**

Is the *Tcte1* mutation causative for male infertility?

**SUMMARY ANSWER:**

Our collected data underline the complex and devastating effect of the single-gene mutation on the testicular molecular network, leading to male reproductive failure.

**WHAT IS KNOWN ALREADY:**

Recent data have revealed mutations in genes related to axonemal dynein arms as causative for morphology and motility abnormalities in spermatozoa of infertile males, including dysplasia of fibrous sheath (DFS) and multiple morphological abnormalities in the sperm flagella (MMAF). The nexin–dynein regulatory complex (N-DRC) coordinates the dynein arm activity and is built from the DRC1–DRC7 proteins. DRC5 (TCTE1), one of the N-DRC elements, has already been reported as a candidate for abnormal sperm flagella beating; however, only in a restricted manner with no clear explanation of respective observations.

**STUDY DESIGN, SIZE, DURATION:**

Using the CRISPR/Cas9 genome editing technique, a mouse *Tcte1* gene knockout line was created on the basis of the C57Bl/6J strain. The mouse reproductive potential, semen characteristics, testicular gene expression levels, sperm ATP, and testis apoptosis level measurements were then assessed, followed by visualization of N-DRC proteins in sperm, and protein modeling *in silico*. Also, a pilot genomic sequencing study of samples from human infertile males (n = 248) was applied for screening of *TCTE1* variants.

**PARTICIPANTS/MATERIALS, SETTING, METHODS:**

To check the reproductive potential of KO mice, adult animals were crossed for delivery of three litters per caged pair, but for no longer than for 6 months, in various combinations of zygosity. All experiments were performed for wild-type (WT, control group), heterozygous *Tcte1^+/−^* and homozygous *Tcte1^−/−^* male mice. Gross anatomy was performed on testis and epididymis samples, followed by semen analysis. Sequencing of RNA (RNAseq; Illumina) was done for mice testis tissues. STRING interactions were checked for protein–protein interactions, based on changed expression levels of corresponding genes identified in the mouse testis RNAseq experiments. Immunofluorescence *in situ* staining was performed to detect the N-DRC complex proteins: Tcte1 (Drc5), Drc7, Fbxl13 (Drc6), and Eps8l1 (Drc3) in mouse spermatozoa. To determine the amount of ATP in spermatozoa, the luminescence level was measured. In addition, immunofluorescence *in situ* staining was performed to check the level of apoptosis via caspase 3 visualization on mouse testis samples. DNA from whole blood samples of infertile males (n = 137 with non-obstructive azoospermia or cryptozoospermia, n = 111 samples with a spectrum of oligoasthenoteratozoospermia, including n = 47 with asthenozoospermia) was extracted to perform genomic sequencing (WGS, WES, or Sanger). Protein prediction modeling of human-identified variants and the exon 3 structure deleted in the mouse knockout was also performed.

**MAIN RESULTS AND THE ROLE OF CHANCE:**

No progeny at all was found for the homozygous males which were revealed to have oligoasthenoteratozoospermia, while heterozygous animals were fertile but manifested oligozoospermia, suggesting haploinsufficiency. RNA-sequencing of the testicular tissue showed the influence of *Tcte1* mutations on the expression pattern of 21 genes responsible for mitochondrial ATP processing or linked with apoptosis or spermatogenesis. In *Tcte1^−/−^* males, the protein was revealed in only residual amounts in the sperm head nucleus and was not transported to the sperm flagella, as were other N-DRC components. Decreased ATP levels (2.4-fold lower) were found in the spermatozoa of homozygous mice, together with disturbed tail:midpiece ratios, leading to abnormal sperm tail beating. Casp3-positive signals (indicating apoptosis) were observed in spermatogonia only, at a similar level in all three mouse genotypes. Mutation screening of human infertile males revealed one novel and five ultra-rare heterogeneous variants (predicted as disease-causing) in 6.05% of the patients studied. Protein prediction modeling of identified variants revealed changes in the protein surface charge potential, leading to disruption in helix flexibility or its dynamics, thus suggesting disrupted interactions of TCTE1 with its binding partners located within the axoneme.

**LARGE SCALE DATA:**

All data generated or analyzed during this study are included in this published article and its supplementary information files. RNAseq data are available in the GEO database (https://www.ncbi.nlm.nih.gov/geo/) under the accession number GSE207805. The results described in the publication are based on whole-genome or exome sequencing data which includes sensitive information in the form of patient-specific germline variants. Information regarding such variants must not be shared publicly following European Union legislation, therefore access to raw data that support the findings of this study are available from the corresponding author upon reasonable request.

**LIMITATIONS, REASONS FOR CAUTION:**

In the study, the *in vitro* fertilization performance of sperm from homozygous male mice was not checked.

**WIDER IMPLICATIONS OF THE FINDINGS:**

This study contains novel and comprehensive data concerning the role of *TCTE1* in male infertility. The *TCTE1* gene is the next one that should be added to the ‘male infertility list’ because of its crucial role in spermatogenesis and proper sperm functioning.

**STUDY FUNDING/COMPETING INTEREST(S):**

This work was supported by National Science Centre in Poland, grants no.: 2015/17/B/NZ2/01157 and 2020/37/B/NZ5/00549 (to M.K.), 2017/26/D/NZ5/00789 (to A.M.), and HD096723, GM127569-03, NIH SAP #4100085736 PA DoH (to A.N.Y.). The authors declare that there is no conflict of interest that could be perceived as prejudicing the impartiality of the research reported.

WHAT DOES THIS MEAN FOR PATIENTS? Abnormal semen parameters (sperm count, motility, and morphology) are the most obvious symptoms that may be related to male fertility problems. The main structural element responsible for sperm motility is the sperm flagellum (tail), which is controlled by a specific sperm structure, the axoneme. This study aimed to determine the role of *Tcte1* gene in male infertility using a mouse model of the gene inactivation. The Tcte1 protein is one of the structural elements building N-DRC, a complex within the sperm axoneme that is responsible for the coordination of activities of the sperm tail related to sperm movement. We have found that mutations in the mouse *Tcte1* switched-off gene model revealed two phenotypes, dependent on the number of affected alleles (or zygosity): infertile homozygotic males (with two identical switched-off alleles) with oligoasthenoteratozoospermia (reduced sperm number, motility and morphology) and fertile heterozygotic males (with one switched-off and normal allele) with oligozoospermia (reduced sperm number only). A pilot study on human samples with *TCTE1* gene variants revealed a wide spectrum in quality of semen including non-obstructive azoospermia (no spermatozoa in ejaculate), cryptozoospermia (few spermatozoa in ejaculate), and severe oligoasthenozoospermia (severely reduced sperm number and motility). Thus, the *TCTE1* gene is the next one that should be added to the ‘male infertility list’ because of its crucial role in spermatogenesis, affecting a variety of testicular molecular networks (including energy machinery processing) and proper sperm function.

## Introduction

Infertility has been defined by the World Health Organization as a social disease concerning ∼10–18% of couples of reproductive age who are unable to conceive within 1 year of regular unprotected sexual intercourse (WHO, 2020). Approximately 7% of males and ∼12% of females worldwide have reproductive problems, while the male factor is responsible for ∼20–30% of all infertility cases (Barratt *et al.*, 2017; Vander Borght and Wyns, 2018; Agarwal *et al.*, 2021). Male infertility is a disease with multifactorial etiology, including genetic factors, chromosomal aberrations, epigenetic mutations, hormonal abnormalities, infections, or reproductive tract abnormalities (Matzuk and Lamb 2008; Zorrilla and Yatsenko, 2013; Krausz and Riera-Escamilla, 2018; Agarwal *et al.*, 2021). Genetic causes of male infertility can be detected in ∼10–15% of cases, while for 25–50% of infertile males, the etiology remains unexplained (Matzuk and Lamb, 2008; Zorrilla and Yatsenko, 2013; Agarwal *et al.*, 2021). The male infertility phenotype mostly results in abnormal semen parameters, including low sperm concentration, abnormal morphology, or diminished motility. The actual standards for genetic testing of male infertility rely on chromosomal screening for structural and numerical abnormalities, followed by analyses of deletions in the AZF (azoospermia factor) regions of the Y chromosome (Hwang *et al.*, 2018). However, it is still not sufficient due to the fact that ∼2000 genes may be related to spermatogenesis, while only ∼10% of them seem to have a documented fertility role, so far (Krausz and Riera-Escamilla, 2018; Houston *et al.*, 2021). In recent years, the development of NGS technology, including genomic sequencing of DNA (exome or genome for large cohorts of patients) and RNA (RNAseq, scRNAseq on reproductive tissues), followed by animal models of infertility (mostly mouse models with more than 860 genes evaluated, so far (Mouse Genome Informatics, 24 June 2021)) have started the new era of exploration on a massive scale (Wyrwoll *et al.*, 2020; Hardy *et al.*, 2021; Houston *et al.*, 2021; Oud *et al.*, 2021; Xavier *et al.*, 2021).

Besides the genes related to spermatogenesis and low sperm count, also studies of candidates responsible for diminished sperm motility are pending. The main structural element responsible for motility, the sperm flagellum, has to be of high quality to guarantee sperm motility and then fertilization success. It is known that mutations in genes encoding factors involved in the ciliogenesis process, lead to defects in the structure and/or functioning of cilia (Girardet *et al.*, 2019; Sironen *et al.*, 2020; Kumar and Reiter, 2021; Smith *et al.*, 2020). There are plenty of data concerning respiratory cilia; however, still little is known about the axonemal dynein arms functioning in the sperm tail. The latest data have revealed mutations in genes related to axonemal dynein arms as causative for morphology and motility abnormalities in the spermatozoa of infertile males (Girardet *et al.*, 2019; Sironen *et al.*, 2020; Aprea *et al.*, 2021; Lorès *et al.*, 2021; Touré *et al.*, 2021; Martinez *et al.*, 2022). Two definitions were established: (i) for sperm cells with disorganization in axonemal structures, so-called dysplasia of fibrous sheath (DFS) (Chemes *et al.*, 1998), and (ii) for multiple morphological abnormalities in the sperm flagella (MMAF), concerning all possible defects of the sperm tail, mainly short and thick flagella, and a lack of the central pair (CP) of microtubules or dynein arms (Ben Khelifa *et al.*, 2014). The observed spectrum of abnormal sperm tail phenotypes allows the qualification of some of the disorders into both listed groups (DFS and MMAF) (Sironen *et al.*, 2020; Touré *et al.*, 2021).

The basic structural element of motile cilia and flagella is the axoneme which is built from nine microtubules arranged in doublets positioned radially and one central pair (CP) of singlet microtubules. This central pair is connected to radial spokes (RS), which are: protein complexes of the mechanical regulatory network responsible for the regular waveform beating of the cilia (Gui *et al.*, 2019). RS are linked to inner dynein arms (IDA) localized within each outer doublet. Each outer doublet carries outer dynein arms (ODA), which are: multi-subunit motor protein complexes, responsible for the generation and regulation of the sliding between dynein arms leading to ATP-dependent beating (Satir *et al.*, 2014; Wilson *et al.*, 2015). The 9 + 2 basic structure of the axoneme is evolutionary conserved (Ishikawa 2017; Girardet *et al.*, 2019; Touré *et al.* 2021). Doublets are linked with the nexin–dynein regulatory complex (N-DRC), which coordinates the dynein arm activity and stabilizes the attachment of doublet microtubules. The N-DRC is built from the DRC1–DRC7 proteins, among which DRC1, DRC2, and DRC4 are responsible for physical linkage between the A and B microtubules of doublets (Wirschell *et al.*, 2013; Bower *et al.*, 2018; Gui *et al.*, 2019; Morohoshi *et al.*, 2020). In terms of male infertility, the best studied gene among the N-DRC is *DRC7* (MIM: 618769), which is amenable for proper assembly of the N-DRC structure (in *Drc7^−/−^* male mice, the length of the sperm tail was reduced, followed by abnormalities of sperm head morphology) (Morohoshi *et al.*, 2020). A mouse model of the *Drc6* (*Fbxl13*) gene (MIM: 609080) revealed no essential role in sperm formation and motility (Morohoshi *et al.*, 2020); however, its disruption together with CEP192 (MIM: 616426) leads to defects in HEK293T cell motility (Fung *et al.*, 2018). Mutations in the *Drc3* gene (MIM: 618758) indirectly revealed male infertility as the reason for a lack of pregnancy with wild-type females (Ha *et al.*, 2016).

**Figure 1.**
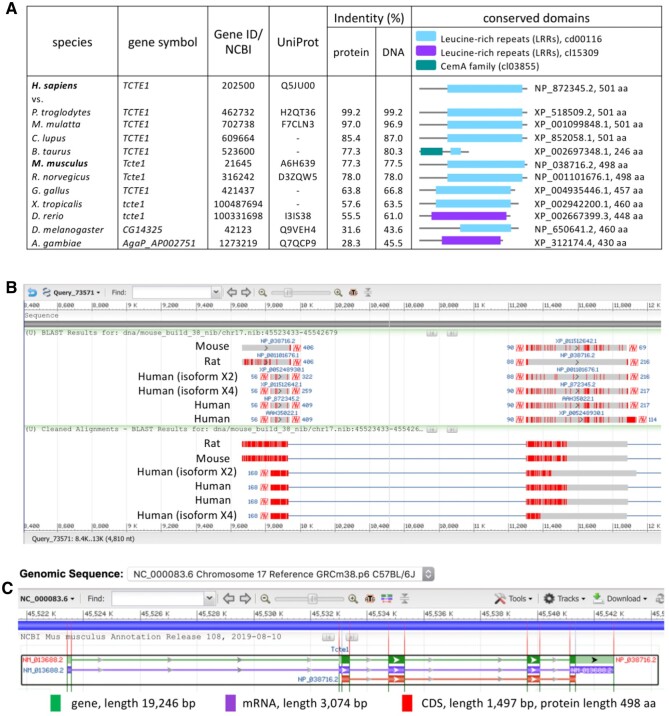
**Characteristics of the *Tcte1* gene**. (**A**) Putative homologs of the *Tcte1* gene conserved in Bilateria, according to HomoloGene (8434), including collation of proteins and their conserved domains. (**B**) Sequence alignment between human, mouse, and rat. (**C**) NCBI Gene schematic representation of the *Tcte1* gene in *Mus musculus*.

One of the N-DRC structural genes is *TCTE1* (T-Complex-associated-Testis-Expressed 1), also known as *DRC5*, *FAP155*, and *D6S46* in humans (*locus* 6p21.1; MIM: 186975) or *Tcte1*, *D17S*, *Tcte-1*, and *D17Sil1* in mice (*locus* 17 B3; 17 22.54 cM; MGI: 98640). *TCTE1* is evolutionary conserved in most eukaryotic organisms that contain motility cilia or flagella in their life cycle (Fig. 1) and is required for proper flagella functioning. Expression of *TCTE1* is observed to be highest in the testis (with expression starting at the spermatid stage) but is also present in the choroid plexus and Fallopian tube. TCTE1 contains five leucine-rich repeats (LRRs). To date, only two reports have described a role for the *Tcte1* gene: as a mice model of infertility, where the *Tcte1*-null sperm cells exhibited asthenozoospermia, or in a group of patients without a clear explanation of the linkage between the observed variant and sperm phenotype (Castaneda *et al.*, 2017; Zhou *et al.*, 2022). Thus, our understanding of the nature of this specific motility-type defect in *Tcte1*-null observations has been limited so far.

In our study, we aimed to determine the role of *Tcte1* in male infertility using a mouse knockout model. We created homo- and heterozygous animals, which were then examined for their reproductive potential, differences in anatomy and histology, and semen parameters. We also performed RNAseq in testis tissue to obtain an answer about the influence of *Tcte1* null mutations on the expression pattern of other gonadal genes. In addition, ATP measurements of spermatozoa were performed. Finally, human samples from males with severely decreased sperm counts were screened for *TCTE1* variants, followed by protein prediction modeling of the revealed variants. Thus, this study contains novel and comprehensive data concerning the role of *TCTE1* in male infertility.

## Materials and methods

### Animals

**Figure 2.**
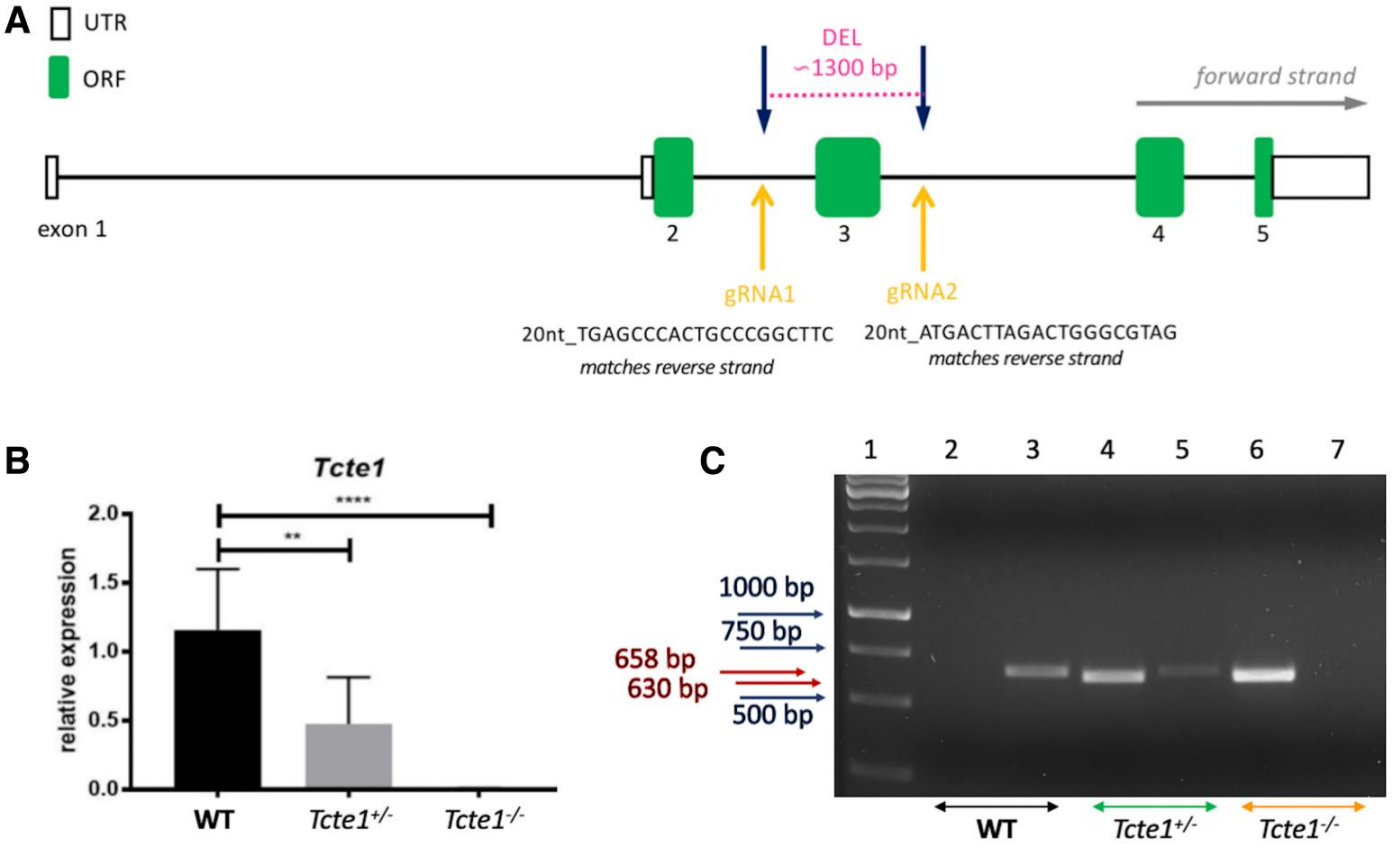
**Characteristics of *Tcte1* knockout mice**. (**A**) Schematic representation of gRNA localization in CRISPR/Cas9 KO creation. (**B**) Relative expression of the *Tcte1* gene in wild-type (WT), hetero- ^(+/−)^ and homozygous mice ^(−/−)^ (Real-time PCR); **** *P* < 0.0001, ** *P* < 0.01. (**C**) Example of genotyping result. Lane 1: 1 kb marker (GeneRuler), lanes 2, 3: wild-type (WT), lanes 4, 5: hetero- ^(+/−)^, and lanes 6, 7: homozygous mice ^(−/−)^; lanes: 2, 4, 6 identified the lack (0 bp) or presence of mutation (630 bp), lanes: 3, 5, 7 differentiated hetero- (658 bp) and homozygotes (0 bp).

Using the CRISPR/Cas9 genome editing technique, the mouse heterozygous knockout line of the *Tcte1* gene (*Tcte1^+/−^*) was created on the basis of the C57Bl/6J strain by the Cyagen Biosciences company (Santa Clara, CA, USA). The mouse *Tcte1* gene is located on chromosome 17 B3; 17 22.54 cM (GenBank: NM_013688.2; Ensembl: ENSMUSG00000023949). Exon 3 of the *Tcte1* gene was selected as the target site (deletion of 1292 bp) (Fig. 2A). gRNAs targeting vectors (constructed, sequenced, and generated by *in vitro* transcription; Supplementary Data File S1) were injected into fertilized oocytes. The founders were genotyped and those with positive results were bred to the next generation. Then, in the animal facility of the Institute of Human Genetics PAS (Poznan, Poland), adult *Tcte1^+/−^* animals were mated to generate homozygous *Tcte1^−/−^* mice. Wild-type *Tcte1^+/+^* (WT) animals C57Bl/6J were purchased from Charles River Company (Sulzfeld, Germany). Relative expression of the *Tcte1* gene was verified using the real-time PCR method (Fig. 2B; Supplementary Data File S2). All mice were housed in a pathogen-free animal facility with food and water *ad libitum*, a stable temperature of 22°C, and a light: dark light cycle of 12:12 h. All procedures were in accordance with the guidelines and regulations for animal experimentations and restricted usage of genetically modified organisms (GMO) and were approved by the Local Ethical Committee for Animal Experiments at the Poznan University of Life Sciences (no. 1/2014, 18/2017), and Ministry of Environment (no. 70/2017). Mouse models with infertility phenotypes were reviewed in the Mouse Genome Informatics database (Jackson Laboratory; http://www.informatics.jax.org/) and through literature searches (PubMed; https://pubmed.ncbi.nlm.nih.gov/).

### Reproductive potential

To check the reproductive potential of KO mice, adult animals (7–8 weeks old) were crossed for delivery of three litters per caged pair, but no longer than for 6 months. Crossing combinations included: (i) four pairs: homozygous *Tcte1^−/−^* male × wild-type (WT) female, (ii) five pairs: heterozygous *Tcte1^+/−^* male × heterozygous *Tcte1^+/−^* female, and (iii) three pairs: heterozygous *Tcte1^+/−^* male × wild-type (WT) female. Vaginal plug presence was checked every morning to confirm mating incidence. The time period from the mating start point to the first pedigree appearance was checked. Numbers of pups were counted on the day after birth. For each crossed pair, the number of litters, pups, and the sex ratio were estimated (also according to pups’ genotypes) (Supplementary Table S1).

### Genotyping

Genotyping of the pups delivered was performed via a PCR assay, with two sets of primers: (i) to identify wild-type and positive KO (heterozygous and homozygous) animals (Tcte1-F: CGTTTTAAGAATGTATTGAGGGTTGGG, Tcte1-R: CTCCGTAGGCTCCTGCCAATATG; estimated product size: for WT 1915 bp, for KO ∼630 bp (deleted ∼1300 bp); PCR results analysis: one band of 1915 bp or 0 bp determined for WT, with one band of 630 bp evidenced for hetero- or homozygotes); and (ii) to distinguish between hetero- and homozygotes (Tcte1-WT/He-F: AGCTTGCCACACCCTCAAGGTACTA, Tcte1-R: CTCCGTAGGCTCCTGCCAATATG; estimated product size: 658 bp for heterozygotes and 0 bp for homozygotes; PCR results analysis: one band of 658 bp determined for heterozygotes, with no band (0 bp) indicated for homozygotes (Fig. 2C). The PCR mixtures and reaction conditions are described in Supplementary Data File S3.

### Tissue gonadal preparation/collection

Gross anatomy was performed on testis and epididymis of WT (n = 21), *Tcte1^+/−^* (n = 22), and *Tcte1^−/−^* (n = 18) male animals (10 weeks old). Mice were weighed, and then euthanized with a carbon dioxide (CO_2_) overdose (euthanasia Minerve Easy-box; Minerve; Esternay, France) with a stable flow rate per minute. Testes and epididymides were collected, weighed, and measured. For histological evaluation, two fixing approaches were applied. Both approaches started with anaesthetization under 4% isoflurane (Baxter, Warsaw, Poland) and transcardial perfusion with 50 ml/animal of ice-cold PBS (Gibco, Paisley, UK), followed by 50 ml/animal of 10% formalin solution, neutrally buffered (Sigma Aldrich, St. Louis, MO, USA, cat. no. HT501850) for three males per zygosity and technical variant. Next, the tissues were immersed in formalin solution for 24 h, 4°C, in darkness. Then, in the first approach, Paraplast-embedded sections were prepared (5 µm thickness) using a Leica 2055 Microtome (Leica, Deer Park, IL, USA). In the second technique, gonads were sequentially immersed in 15%, 20% and 30% of sucrose (Sigma Aldrich, cat. no. S0389), each for 24 h, at room temperature. Thereafter, gonads were coated with Tissue-Tek O.C.T. Compound (Sakura, Alphen aan den Rijn, The Netherlands, cat. no. 4583) and frozen in cold (−80°C) 2-Methylbutane (Sigma Aldrich, cat. no. M32631). Then, cryo-sections were prepared (10 µm thickness), using a Leica CM1950 Cryostat (Leica Biosystems, Nussloch, Germany) and kept at −80°C until further stainings. To obtain the histological picture, slides were stained using the Masson-Goldner protocol (Romeis *et al.*, 2010) for classic paraffin blocks or standard progressive H&E staining method for frozen samples. Briefly, after fixation twice in 100% EtOH (PoCH, Gliwice, Poland) (10 min), slides were first hydrated in an EtOH series (95%, 70% for 5 min), washed in distilled water and stained in Mayer’s hematoxylin (Sigma Aldrich) for 5 min, then washed in distilled water (three times, 5 min) and dehydrated in 50%, 70%, 80%, 96% EtOH (5 min each). Counterstaining with eosin Y (Sigma Aldrich) for 30 seconds was applied, followed by washing in 100% EtOH, clearing in xylene (PoCH) (5 min each, two repeats), and mounting in DPX (Sigma Aldrich). Thereafter, slides were documented with the Leica DM5500 light microscope (×40 dry or ×63 oil immersive objectives, motorized stage), LASX software (with Navigator tool) (Leica Microsystems GmbH, Wetzlar, Germany). All the evaluations were performed blindly by two experienced scientists per technique.

For each zygosity, the geometrical features of the tubule sections were evaluated, including tubule diameter (D) and cell layer thickness (CL) (the mean of two measurements made at opposite sides of the tubule) and lumen diameter (l), using the Fiji (Image J; University of Wisconsin, USA) measurement tool. To check density of the cells, ratios of D: CL, D: l and CL: l were also estimated. In total, for each zygosity, 133–144 tubule sections were evaluated (three random sections per zygosity, each with a minimum of 30 tubules).

### Mouse semen samples

Mouse spermatozoa were collected from epididymis *post mortem*. Collected epididymis were placed immediately in a 2.0 ml test tube with warm 500 µl of a non-capacitating Whitten’s HEPES medium (pH 7.2–7.4, 37°C), then cut into 5–7 fragments, and incubated in 37°C for 15 min to allow the sperm cells to swim out. Whittens-HEPES medium contained: 100.0 mM NaCl, 4.4 mM KCl, 1.2 mM KH_2_PO_4_, 1.2 mM MgSO_4_, 5.4 mM glucose, 0.8 mM pyruvic acid, 4.8 mM lactic acid (hemi-Ca), 20 mM HEPES (all from Sigma Aldrich) (Wertheimer *et al.*, 2013). Then, spermatozoa were collected and seminological analysis performed (concentration, motility, morphology). Next, semen samples were fixed in a fresh fixative solution (methanol: acetic acid, 3:1 v/v, −20°C; 3×; both from PoCH) and stored until further use at −20°C.

### Semen analysis

A 10-μl aliquot of sperm suspension was applied to the Makler counting chamber (Sefi Medical Instrument Ltd, Haifa, Israel) to test the sperm concentration and total sperm count. Also, the ratio of progressive (fast and slow), non-progressive (total and circular), and immotile spermatozoa were calculated (WHO, 2021). For assessment of sperm morphology, Papanicolaou staining was performed. Briefly, the air-dried smears were fixed in ethanol: diethyl ether (1:1 ratio, vol/vol; PoCH) solution for 10 min and left to air-dry. Then, slides were stained by the Papanicolau procedure according to WHO guidelines (WHO, 2021), and mounted in DPX Mountant (Sigma Aldrich, cat. no. 06522). A total of 200 spermatozoa were analyzed using a Leica DM5500 light microscope (×1000 magnification, oil immersion). Various morphological forms of spermatozoa were determined including abnormalities of the head, midpiece, and tail. Also, the length of the sperm tail, midpiece, and the tail:midpiece ratio were measured to check possible differences between males with various zygosities (6 males/per zygosity); for each male, at least 100 spermatozoa were measured with the ‘length measurement’ option by CellSens Dimension software (Olympus BX41, Tokyo, Japan).

### Sequencing of RNA

For RNA extraction, testis tissue was collected in RNAlater buffer (Invitrogen, Waltham, MA, USA) and frozen immediately (−80°C). Samples were processed using the AllPrep DNA/RNA/Protein Mini Kit (Qiagen, Hilden, Germany). Quality and quantity of extracted RNA samples were checked using NanoDrop (ND-1000, Thermo Scientific, Waltham, MA, USA) and Quantus (Promega, Madison, WI, USA), and 1 μg of RNA per sample was used for sequencing (Macrogen, Seoul, South Korea). RNA sequencing was performed with the Illumina NovaSeq 6000 platform using TruSeq Stranded mRNA LT Sample Prep Kit (Illumina), with specified conditions of paired-end reads at 100 bp, and throughput 100 M reads per sample. RNASeq raw reads were aligned to the mouse reference genome mm10 using hisat 2.0.5 with Gencode vM13 transcriptome reference. Reads aligned in the coding regions of the genome were counted using Feature Counts. Then, read counts were normalized using DESeq2 and subjected to differential analysis. The differential analysis included computing of median-based fold change, statistical significance using the Students’ *t*-test, and false discovery rates (correction for multiple testing) in the R/Bioconductor programming environment. Testis tissue expression was retrieved from GTEx (https://gtexportal.org/home/), BioGPS (http://biogps.org/), and AceView (https://www.ncbi.nlm.nih.gov/IEB/Research/Acembly/). All RNAseq expression data have been deposited in Gene Expression Omnibus (GEO), accession number GSE207805.

### STRING interactions

For analysis of protein–protein interactions, based on changed expression levels of corresponding genes identified in the mouse testis RNAseq experiments, STRING analysis was performed (version 11.0). The STRING database allows to delineate probability of the interactions between evaluated proteins using different evidence channels (von Mering *et al.*, 2005; Szklarczyk *et al.*, 2019). This tool enables the integration of all data available in public resources concerning protein–protein interactions.

### Immunofluorescence on sperm: N-DRC visualization

Immunofluorescence *in situ* staining was performed to detect the N-DRC complex proteins: Tcte1 (Drc5), Drc7, Fbxl13 (Drc6), and Eps8l1 (Drc3) in mouse spermatozoa. Mouse spermatozoa were collected and fixed as described above. Specific antibodies were applied as primary antibodies: (rabbit anti-human-TCTE1 (cat. no. orb357083), rabbit anti-mouse-Drc7 (orb586958), anti-mouse-Fbxl13 (orb314611), and anti-mouse-Eps8l1 (orb382538; all from Biorbyt, Cambridge, UK)), or the secondary antibody: (goat anti-rabbit-AF594 (Abcam, Cambridge, UK, cat. no. ab150160)). The UniProt IDs and homology of the immunization regions of primary antibodies between human and mouse are described in Supplementary Table S2. Antibodies were diluted in 0.5% PBST (PBS: Gibco; Triton X-100: Sigma Aldrich) (primary: 1:100, secondary: 1:500). First, slides with fixed sperm smears were washed in a series of washes in 1× PBST, and then incubated in 25 mM DTT/1 M Tris-HCl (Sigma Aldrich), pH 9.5, at room temperature for 20 min. Then, washing in 1× PBST, and incubation in 6 N HCl (PoCH) for 30 min was applied, followed by a blocking step with 1% BSA/1× PBST for 30 min (BSA: Sigma Aldrich). Next, overnight incubation with antibodies was performed at 4°C in a humidified container. After washing, the samples with 1× PBST, a secondary AF594-conjugated antibody was applied for 1 h. Next, unconjugated antibodies were washed out (4× in 1× PBST, 5 min each, slight rotation). For the final detection, 20 µl of Fluoroshield with DAPI (Sigma Aldrich, cat. no. F6057) was added to the samples, and microscopic analysis was performed. Images were acquired using a fluorescence microscope with a proper filter set: Leica DM5500, filters: DAPI/TxR/Triple; objectives: 10× and 63× with oil immersion; DFC 7000T camera; software: LASX.

### Sperm ATP measurement

For measurement of ATP in spermatozoa, the CellTiter-Glo 2.0 Assay (Promega) was used. This ready-to-use assay allows to determine the quantitation of the ATP amount, indicating the presence of metabolically active cells. The assay is based on the thermostability of luciferase (Ultra-GloTM Recombinant Luciferase) and was performed as described in the manufacturer’s protocol. Briefly, all laboratory work was done in a stable temperature of 22°C. After the collection of mouse spermatozoa (as described above), the amount of ∼1 × 10^6^ of sperm cells was transferred to a black opaque 1.5 ml test tube and the proper amount of CellTiter-Glo kit was added (1:1, v: v). Next, 2 min of orbital mixing of the content was performed to activate cell lysis. Then, after 10 min of incubation (to stabilize the luminescence), the measurement of the luminescent signal was completed using a Glomax 20/20 luminometer (Promega). For each sample, three measurements (repeats) were done, each in two time points of 0 and +60 min. Numbers of animals used were: WT (n = 7), *Tcte1^+/−^* (n = 7), and *Tcte1^−/−^* (n = 6). Mean luminescence values obtained (represented as RLU: relative luminescence unit) were compared between tested mice groups.

### Immunofluorescence on testis

Immunofluorescence *in situ* staining on testis was performed to check: (i) the mean cell count of spermatogonia and spermatocytes (preleptotene and pachytene) in tubules’ sections (cryo-sections), and (ii) to estimate the level of apoptosis via caspase-3 visualization on mouse testicular samples (paraffin embedded sections) from all three zygosities. Caspases play an important role in the induction, transduction, and amplification of intracellular apoptotic signals, and thus, caspase-3 can be used as one of the apoptotic indicators (Lei *et al.*, 2022). Mouse testes were collected and fixed as described above. Specific antibodies were applied as primary antibodies: (mouse anti-MLH1 (Abcam, cat. no. ab14206), 1:30 in 0.5% PBST, rabbit anti-RAD51 (Abcam, cat. no. ab63801), 1:200 in 0.5% PBST) and: rabbit anti-Casp3, (Biorbyt, cat. no. orb10237), 1:100 in 0.5% PBST) or as secondary antibodies: (goat anti-mouse-FITC (Sigma Aldrich, cat. no. F2012), 1:400 and goat anti-rabbit-AF594 (Abcam, cat. no. 150080), 1:500 in 0.5% PBST). First, slides with paraffin fixed testis cross sections were deparaffinized in three changes of xylene (10 min each), while for cryo-sections, this step was omitted. Then, slides were incubated in a series of ethanol: 100%, 96%, and 70%, twice for 5 mins in each solution. Next, after rinsing in distilled water, tissue sections were incubated sequentially in freshly prepared 2% NaBH_4_/1× PBS, and in 0.1M glycine (30 min, room temp. each; Sigma Aldrich). Heat-induced epitope retrieval was performed by immersing the slides in 0.01 M sodium citrate/0.05% Tween 20 (Sigma Aldrich), pH 6.0 for 30 min, followed by cooling down at room temperature for the next 20 min. A blocking step was performed in an aliquot of freshly prepared 1% BSA/1× PBST, at 37°C for 1 h. Then, tissue sections were incubated with primary antibody in a humidified container overnight at 4°C. A secondary antibody was applied for 1 h, followed by washing out of the unconjugated antibody (4× in 1× PBST, 5 min each, slight rotation). For the final detection, 20 µl of Fluoroshield with DAPI was added. Images were captured using a fluorescence Leica DM5500 microscope with a proper filter set: DAPI/TxR/SpG/Triple; objectives: 10× and 63× with oil immersion; DFC 7000T camera, LASX software. The analysis was performed for three sections per zygosity, each with a minimum of 30 tubules (for cell count) or two cross-sections per zygosity, 15–20 tubules per tissue section (for Casp-3 evaluation).

### TUNEL assay

TUNEL assay was performed to check the level of apoptosis and necrosis on the testicular tissue according to the manufacturer’s protocol (Abcam, ab66110 TUNEL Assay Kit: BrdU-Red). Briefly, paraffin embedded sections (or two cross-sections per zygosity, with 15–20 random tubules analyzed/slide) were deparaffinized in xylene (twice, each 5 min), followed by an EtOH series: 100% (twice, 5 min), 95%, 85%, 70%, and 50% (3 min). Next, slides were incubated in 0.85% NaCl (5 min) and washed in PBS. After incubation with Proteinase K solution (Qiagen) (20 µg/ml in Tris-HCl–EDTA; 5 min) and washing with PBS, slides were immersed in 4% formaldehyde/PBS. Then, Wash Buffer was applied twice for 10 min with a plastic coverslip on the top. Next, DNA Labeling Solution was added followed by incubation in a dark humidified incubator for 1.5 h at 37°C. After the next two washes with PBS, Antibody Solution was applied, and slides were incubated for 30 min at room temperature. The last steps included ddH_2_O washing (5 min, twice) and the application of 20 µl of Fluoroshield with DAPI for the final detection. Slides were analyzed within 1 h using the fluorescence Leica DM5500 microscope with a proper filter set: DAPI/TxR/Triple; objectives: 10× and 63× with oil immersion; DFC 7000T camera, LASX software.

### Sequencing of the human samples

A pilot genomic sequencing study of samples from human infertile males was also performed. Bioethical committee approvals were received for the study (Local Bioethics Committee of Poznan University of Medical Sciences, approval no. 1003/18; National Bioethical Committee, Ministry of Health, Warsaw, Poland, approval no. OKB-5-2/15). All participants were notified about the aim of the study and provided written informed consent. All experiments were performed in accordance with relevant guidelines and regulations. DNA from whole blood samples (collected into sterile single test tubes with EDTA) was extracted using standard protocols of the MagCore HF16 Automated Nucleic Acid Extractor (RBC Biosciences, New Taipei City, Taiwan), or Gentra Puregene kit (Qiagen). The total number of sequenced patients was n = 248, including: 137 with non-obstructive azoospermia (lack of spermatozoa in ejaculate) or cryptozoospermia (spermatozoa detectable in ejaculate after centrifugation), and 111 with a spectrum of seminal abnormalities in concentration, and/or motility, and/or morphology. Among those samples, 47 revealed decreased motility (asthenozoospermia). Semen evaluation was performed according to WHO guidelines, followed by AUA and ASRM guidelines for infertile male diagnosis (Schlegel *et al.*, 2021). All men revealed normal hormonal levels, had no chromosomal abnormalities and no AZF microdeletions. For whole exome samples (n = 76 from Pittsburgh), sequencing libraries were constructed using: the SureSelect XT All Exon V4 + UTRs capture kit and the SureSelect XT Target Enrichment System (Agilent Technologies, Santa Clara, CA, USA). Libraries were sequenced with an average read coverage of 62–100× (∼6 Gb per sample), using 100 bp paired-end sequencing with TruSeq PE Cluster Kit v3 cBot HS and 4 × 50 TruSeq SBS V3 HS sequencing kit (Illumina) on the HiSeq2000 (Illumina). Whole-genome sequencing (n = 42, Poznan) was performed using Illumina HiSeqX with the coverage of at least 30× (100–120 Gb per sample). Sanger sequencing for all exomes of *TCTE1* gene (n = 130, Poznan) was performed using LightRun96 system of Eurofins Genomics (Eurofins Genomics AT GmbH, Vienna, Austria). Samples were prepared with QIAquick Gel Extraction (cat. no. 28704) or PCR Purification (cat. no. 28104) kits (Qiagen), and analyzed with CLC Main Workbench 8 software (Qiagen).

### Filtering of whole-genome sequencing results

The quality of raw NGS data was evaluated using the FastQC and MultiQC packages. Reads were aligned to the reference human genome GRCh37 using bwa-mem aligner included in the Speedseq package (Chiang *et al.*, 2015). For calling single-nucleotide variants (SNVs) and small indels, freeBayes v0.9.21 (speedseq-var) was implemented, following the previously described pipeline (Supernat *et al.*, 2018). Variant disease association and missense mutation prediction effects were determined using: dbSNP (https://www.ncbi.nlm.nih.gov/snp/), OMIM (https://www.ncbi.nlm.nih.gov/omim/), ClinVar (https://www.ncbi.nlm.nih.gov/clinvar/), MutationTaster (http://www.mutationtaster.org/), SIFT (https://sift.bii.a-star.edu.sg/), Polyphen (http://genetics.bwh.harvard.edu/pph2/), CADD (https://cadd.gs.washington.edu/snv), MetaDome (https://stuart.radboudumc.nl/metadome/), REVEL (rare exome variant ensemble learner) (Ioannidis *et al.*, 2016), Ensembl Variant Effect Predictor (McLaren *et al.*, 2016), and the Human Gene Mutation Database (HGMD; Qiagen). SNV pathogenicity was assessed on the basis of American College of Medical Genetics and Genomics (ACMG) guidelines (Richards *et al.*, 2015). PhyloP (Cold Spring Harbor Laboratory) and Clustal Omega were used for the determination of conservation (https://www.ebi.ac.uk/Tools/msa/clustalo/). All NGS variants were confirmed via the Integrative Genomics Viewer (Broad Institute). Variant frequencies have been obtained from the gnomAD database (frequencies from more than 125k exomes and 15k genomes, http://gnomad.broadinstitute.org; v2.1.1), and were filtered in search of rare variants (with minor allele frequency (MAF) <1%).

### Protein prediction

The amino acid sequence of TCTE1 was input into Phyre2 (Kelley *et al.*, 2015) for both intensive and fast mode homology modeling, I-TASSER (Roy *et al.*, 2010; Yang *et al.*, 2015, Yang and Zhang, 2015), and AlphaFold (Jumper *et al.*, 2021; Varadi *et al.*, 2022), which utilize sequence alignment-based algorithms for generating predicted secondary and tertiary structures; AlphaFold additionally uses machine learning to integrate biological principles of protein folding with the alignment-based algorithms. Confidence scores were obtained from these programs; AlphaFold additionally generates predicted aligned error plots for assessing the quality of the model’s long-range interactions. Models were inspected manually and aligned using the secondary structure matching (SSM) (Krissinel and Henrick, 2004) tool in COOT (Emsley *et al.*, 2010) to minimize user input bias. Docking of the structures into electron tomography data was performed manually in COOT (Emsley *et al.*, 2010). No refinement was performed to minimize bias. All structure model figures and mutations without post refinement, were generated using Pymol (Schrödinger). I-TASSER was used also for prediction of the exon 3 structure deleted in our mouse KO model (Supplementary Fig. S1).

### Statistical analysis

Statistical analyses included the following: normality test (D’Agostino and Pearson or Kolmogorov–Smirnoff), unpaired two-tailed *t*-test with Welch’s correction or Mann–Whitney test, one-way ANOVA test and Fisher’s exact, and two-tailed Pearson correlation. All tests were performed with a significance level of α = 0.05 using GraphPad Prism (v.7.0e) software.

## Results

### Reproductive potential of KO mice

**Figure 3.**
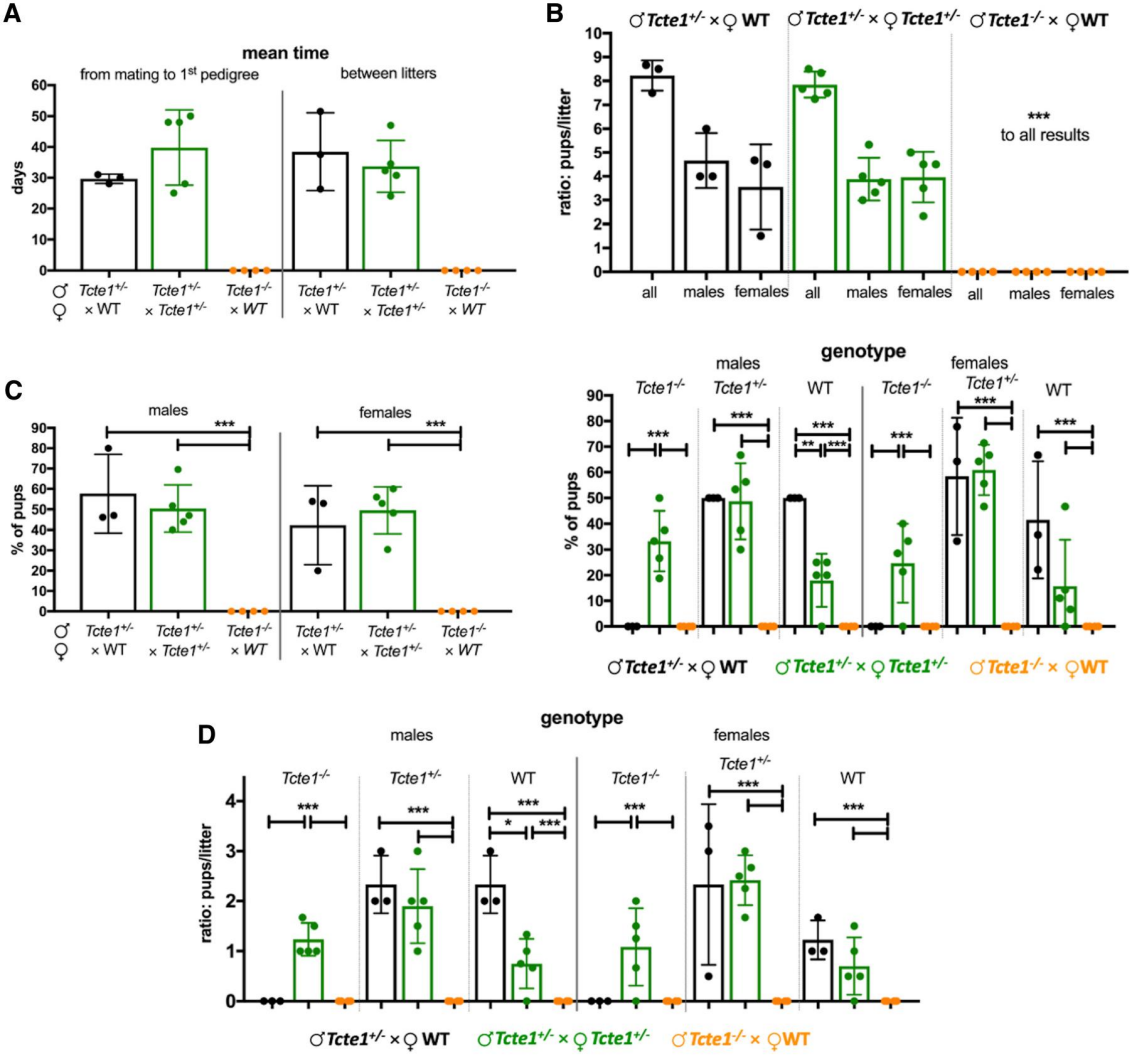
**Reproductive potential of KO *Tcte1* mice**. Mating combinations and their results including time of breeding, number of litters and pups, number of male and female pups, and genotypes observed in pups of each combination. (**A**) Mean time: from mating to first pedigree, and between particular litters. (**B**) Ratio of males and females per litter in each mating combination. (**C**) Frequency of males and females and their genotype observed each mating combination; left panel: according to the sex of pups; right panel: according to mating combinations. (**D**) Ratio of males and females per litter and their genotype observed in each mating combination. ***P < 0.0001, **0.0001 < *P* < 0.01, *0.01 < *P* < 0.05.

Reproductive potential results are shown in Fig. 3 and Supplementary Table S1. Only pairs with homozygous *Tcte1^−/−^* males revealed no progeny (*Tcte1^−/−^* male × WT female). Other combinations (*Tcte1^+/−^* male × *Tcte1^+/−^* female; *Tcte1^+/−^* male × WT female) showed similar results when considering numbers of litters, pups, and sex ratio. The mean time of caging to delivery of the first pedigree was longer in the *Tcte1^+/−^* male × *Tcte1^+/−^* female combination by 25.45% (statistically not significant). When considering genotypes of the pups, statistically significant differences (*P* < 0.05) were observed in the number of *Tcte1^−/−^* male and female pups delivered: no homozygous animals were obtained from the combination of *Tcte1^+/−^* male × WT female, while in mating of *Tcte1^+/−^* male × *Tcte1^+/−^* female, homozygous males (33.25% of all pups) and females (24.67% of all pups) were received.

### Histopathology and semen parameters

**Figure 4.**
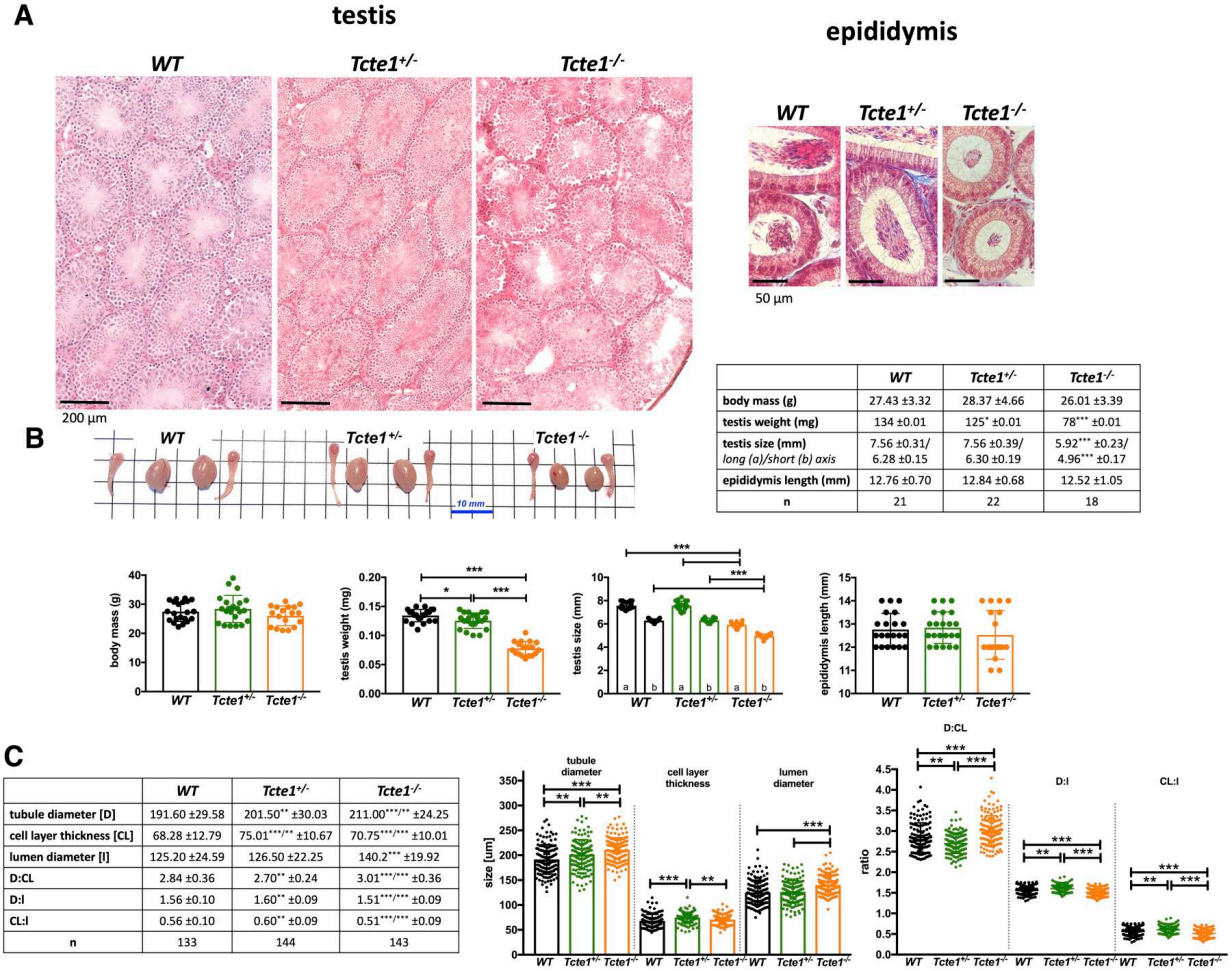
**Characteristics of *Tcte1* wild-type (WT), hetero- ^(+/^** ^−^ **^)^ and homozygous mice ^(^** ^−^^**/**^^−^^**)**^. (**A**) Histopathology of testes and epididymis (caput); staining: classic hematoxylin–eosin staining (testis) or Masson–Goldner protocol (epididymis); microscope: Leica DM5500, objective 40×, LASX software with Navigator function. (**B**) Comparison of body mass, testis weight and size, and epididymis length, followed by gross morphology of gonads (n: no. of mice analyzed). (**C**) Comparison of the dimensions of testicular tubule sections (n: no. of sections analyzed); ***P < 0.0001, **0.0001 < *P* < 0.01, *0.01 < *P* < 0.05.

**Figure 5.**
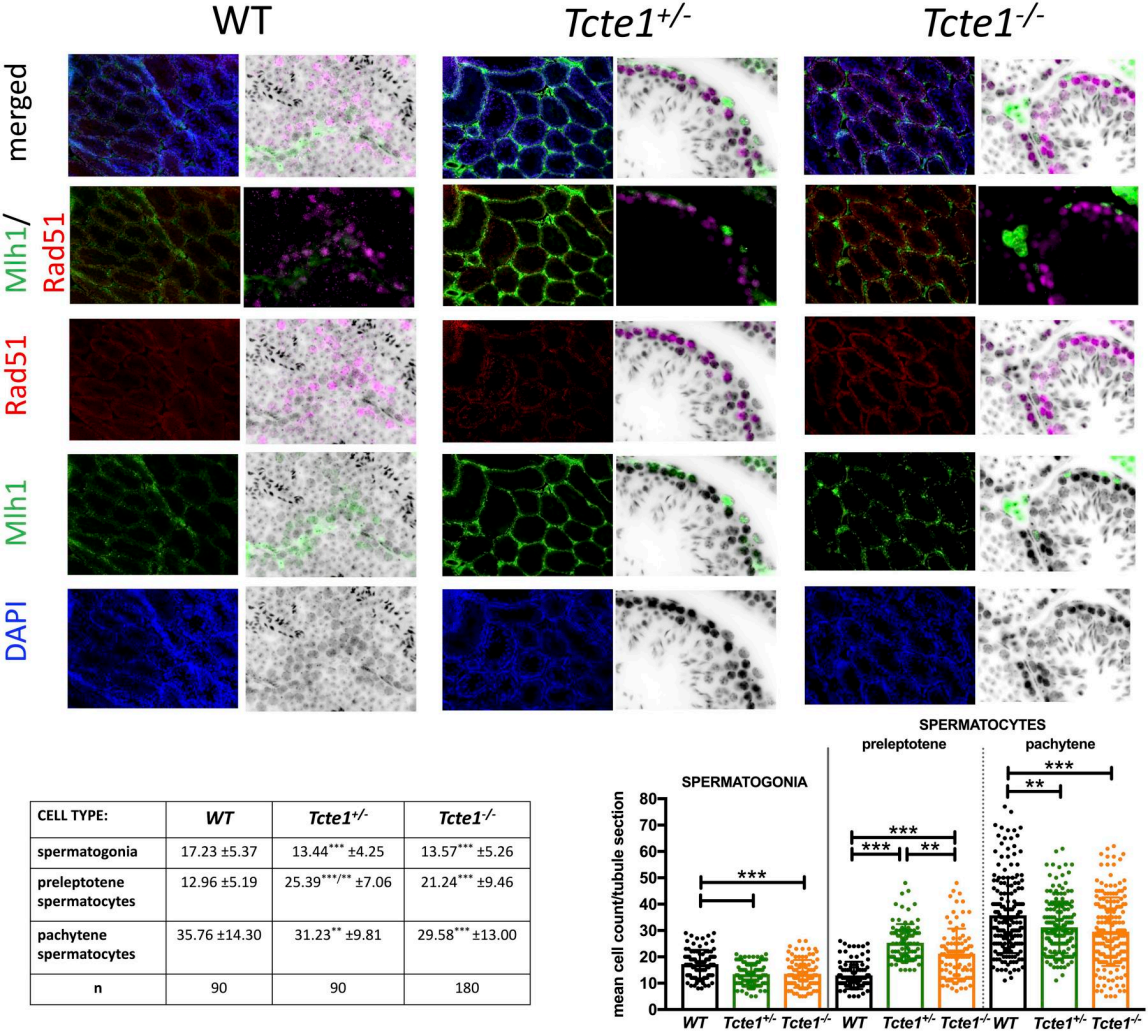
**Evaluation of germ cell types in testes of *Tcte1* wild-type (WT), heterozygous ^(+/^** ^−^ **^)^ and homozygous ^(^** ^−^^**/**^^−^ **^)^ mice**. Three germ cell types were evaluated: spermatogonia (stained with intensive green color), preleptotene spermatocytes (intensive red color), and pachytene spermatocytes (medium green and red). Immunofluorescence staining with antibodies: primary: mouse anti-MLH1 (Abcam, Cambridge, UK, cat. no. ab14206), 1:30, rabbit anti-RAD51 (Abcam, Cambridge, UK, cat. no. ab63801), 1:200; secondary: goat anti-mouse-FITC (Sigma Aldrich, St. Louis, MO, USA, cat. no. F2012), 1:400 and goat anti-rabbit-AF594 (Abcam, Cambridge, UK, cat. no. 150080), 1:500.

Histopathological evaluation of testicular and epididymal sections revealed no visual structural differences between WT, *Tcte1^+/−^*, and *Tcte1^−/−^* males, confirming preserved spermatogenesis (Fig. 4A; Supplementary Fig. S2). No differences were found for body mass of the animals and the length of the epididymis (Fig. 4B). In the *Tcte1^−/−^* males, testis weight was decreased by ∼41% (*P* < 0.0001) followed by a ∼22% decline of its size (*P* < 0.0001), while in *Tcte1^+/−^* males, only testis weight was slightly decreased (∼7%; *P* < 0.05) when compared to control animals. Differences in dimensions of the tubule sections were noted (Fig. 4C), with increasing tubule and lumen diameters and decreasing cell layer thickness in *Tcte1^−/−^*. In *Tcte^+/−^*, cell layer thickness and tubule diameter were increased. When comparing germ cell counts (Fig. 5), reduced numbers of spermatogonia (∼22%) and pachytene spermatocytes (13–17%) were documented for *Tcte1^−/−^*and *Tcte^+−-^* (*P* < 0.001), while the level of preleptotene spermatocytes was elevated (*P* < 0.001). The mean sum of all evaluated cell types was similar between WT and homozygotes (65.95 and 64.39 of cells/tubule section, respectively). Such data suggest some kind of time shift at the first stages of prophase: germ cells from KO mice seem to be delayed at the preleptotene stage, while WT males enter the pachytene stage at a higher rate.

**Figure 6.**
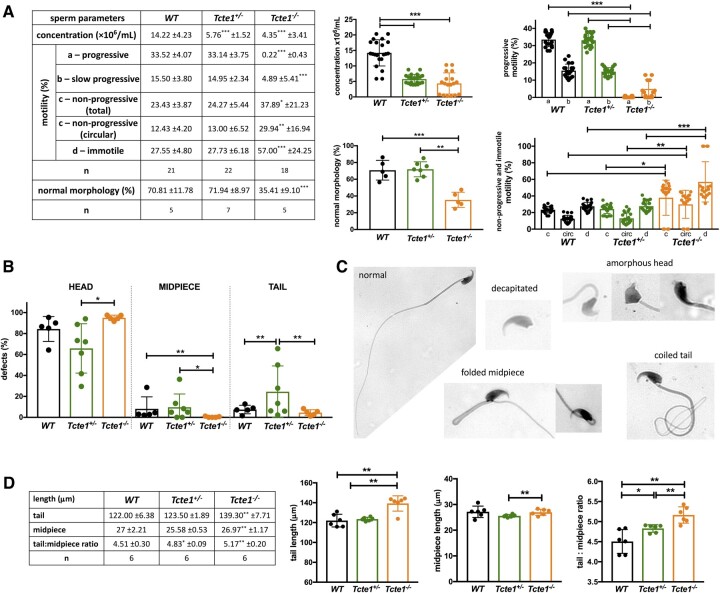
**Characteristics of spermatozoa of *Tcte1* wild-type (WT), heterozygous ^(+/^** ^−^ **^)^ and homozygous ^(^** ^−^^**/**^^−^ **^)^ mice**. (**A**) Sperm parameters: concentration, motility, and morphology comparisons; statistical marks indicate differences according to WT animals; n: number of mice. (**B**) Frequency of morphological defects according to three parts of the spermatozoa. (**C**) Examples of the main sperm morphological abnormalities found (all abnormalities observed available in Supplementary Data File S4); Leica DM5500 microscope, objective 63×, CytoVision software. (**D**) Comparison of the mean length of sperm tail, midpiece and ratio tail:midpiece; n: no. of animals, each with at least 100 spermatozoa analyzed; ***P < 0.0001, **0.0001 < *P* < 0.01, *0.01 < *P* < 0.05.

When considering seminal parameters, both in *Tcte1^−/−^* as well as in *Tcte1^+/−^* males, the sperm concentration was importantly decreased (3.27, and 2.47-fold, respectively), when compared to control results (*P* < 0.0001; Fig. 6A). Additionally, in *Tcte1^−/−^* males, the frequency of progressively motile spermatozoa was residual, with the progressive type of motility in only 0.22% of spermatozoa (decreased ∼150-fold versus control 33.52%, (*P* < 0.0001)), followed by 4.89% being slow progressive spermatozoa (decreased ∼3-fold versus control 15.50%) (*P* < 0.0001). Interestingly, an ∼2.4-fold increase in the circular type of motility (around own axis and non-progressive) was observed for *Tcte1^−/−^* males when compared to WT and *Tcte1^+/−^* animals (*P* < 0.01) (Supplementary Video S1, S2, and S3). The frequency of immotile spermatozoa was also increased in *Tcte1^−/−^* males (2-fold to control; *P* < 0.0001). The frequency of morphologically normal spermatozoa in *Tcte1^−/−^* animals was significantly decreased (2-fold to control; *P* < 0.0001; Fig. 6A–C; Supplementary Data File S4). The majority of morphological defects was observed for the sperm head, mostly of decapitated spermatozoa in *Tcte1^−/−^* animals: 52.55% versus 19.16% for *Tcte1^+/−^* and 23.33% for WT, and amorphous head shape with decreased frequency in *Tcte1^−/−^* (26.11%) and *Tcte1^+/−^* (33.72%) males versus WT animals (48.88%) (Supplementary Data File S4). Midpiece defects were found in *Tcte1^+/−^* (9.71%) and WT (8.20%) animals, while *Tcte1^−/−^* mice revealed only a low frequency (0.38%) of such defects. On the other hand, an increased frequency (∼5-fold) of coiled tail was observed for *Tcte1^+/−/−^* animals when compared to other mice zygosity (*P* < 0.01) (Supplementary Data File S4). To summarize, seminological changes in mice with *Tcte1* mutations resulted in oligoasthenoteratozoospermia in homozygous *Tcte1^−/−^* or oligozoospermia in heterozygous *Tcte1^+/−^* animals.

Increased mean lengths of tail and tail:midpiece ratios in *Tcte1^−/−^* animals (139.30 ± 7.71 µm; 5.16, respectively) were measured when comparing to WT (122.00 ± 6.375 µm; 4.51; *P* < 0.002) and *Tcte1^+/−^* (123.50 ± 1.89 µm; 4.83; *P* < 0.008) (Fig. 6D). Also, in the *Tcte1^+/−^*, the midpiece length differed from *Tcte1^−/−^* animals (*P* = 0.0334), with tail:midpiece ratio differences when compared to WT (*P* = 0.0435).

### RNAseq and STRING pathway analysis

**Figure 7.**
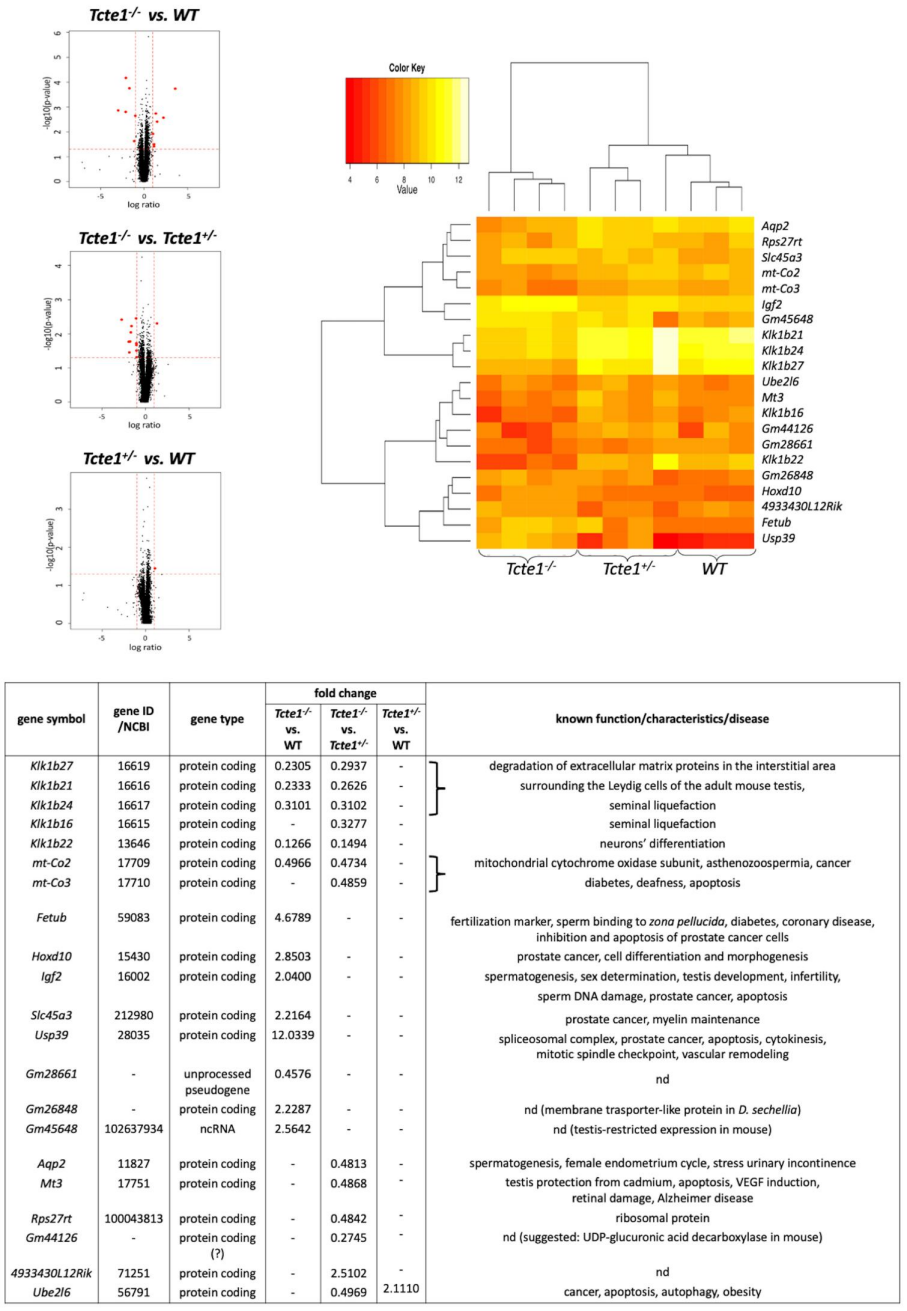
**RNAseq results of gene expression levels found in testes of KO *Tcte1^+/^*** ^−^ **and *Tcte1*** ^−^ **^*/*^** ^−^ **mice versus control (WT) mice**. Volcano plots: black dots indicate all analyzed genes within the genome, red dots represent genes with significantly changed expression levels. A heatmap and a table describing the genes are also shown. Filtering criteria: *P* <0.05, log ratio >1 or <−1 (adequate to fold change value of 2 (expression level increased) or 0.5 (expression level decreased)), expression level >8. nd: no data concerning function or disease.

RNA sequencing analysis of gene expression in testes of KO mice revealed statistically significant changes (*P* < 0.05) in 21 genes, including five genes both for *Tcte1^−/−^* versus *Tcte1^+/−^* and WT, eight genes only for *Tcte1^−/−^* versus WT, seven genes only for *Tcte1^−/−^* versus *Tcte1^+/−^*, and 1 gene both for *Tcte1^+/−^* versus *Tcte1^−/−^* and WT (Fig. 7). In *Tcte1^−/−^* animals versus both *Tcte1^+/−^* and WT, the expression level of four genes from the kallikrein subfamily was decreased 2–8-fold, including genes involved in semen liquefaction and degradation of extracellular matrix proteins in the interstitial area surrounding Leydig cells of the adult mouse testes (*Klk1b27*, *Klk1b21*, *Klk1b24*) or neuronal activity (*Klk1b22*) (Fig. 7; Supplementary Data File S5) (Fink *et al.*, 2007; Du Plessis *et al.*, 2013; Marques *et al.*, 2016; Michaelis *et al.*, 2017). A 2-fold decrease of expression of genes related to mitochondrial cytochrome oxidase subunits and asthenozoospermia was documented (*mt-Co2*, *mt-Co3*) (Fig. 7) (Baklouti-Gargouri *et al.*, 2013; Heidari *et al.*, 2016; Mostafa *et al.*, 2016). In *Tcte1^−/−^* versus WT, eight genes revealed changes in expression level, including a 4.7-fold increase for the *Fetub* gene, which is known to be required for oocyte fertilization (via specific inhibitor activity of the zona pellucida (ZP) ovastacin, the cysteine protease responsible for ZP2 cleavage) (Karmilin *et al.*, 2019; Binsila *et al.*, 2021), and plays also a role of a secreted factor during reprogramming (Bansho *et al.*, 2017).

**Figure 8.**
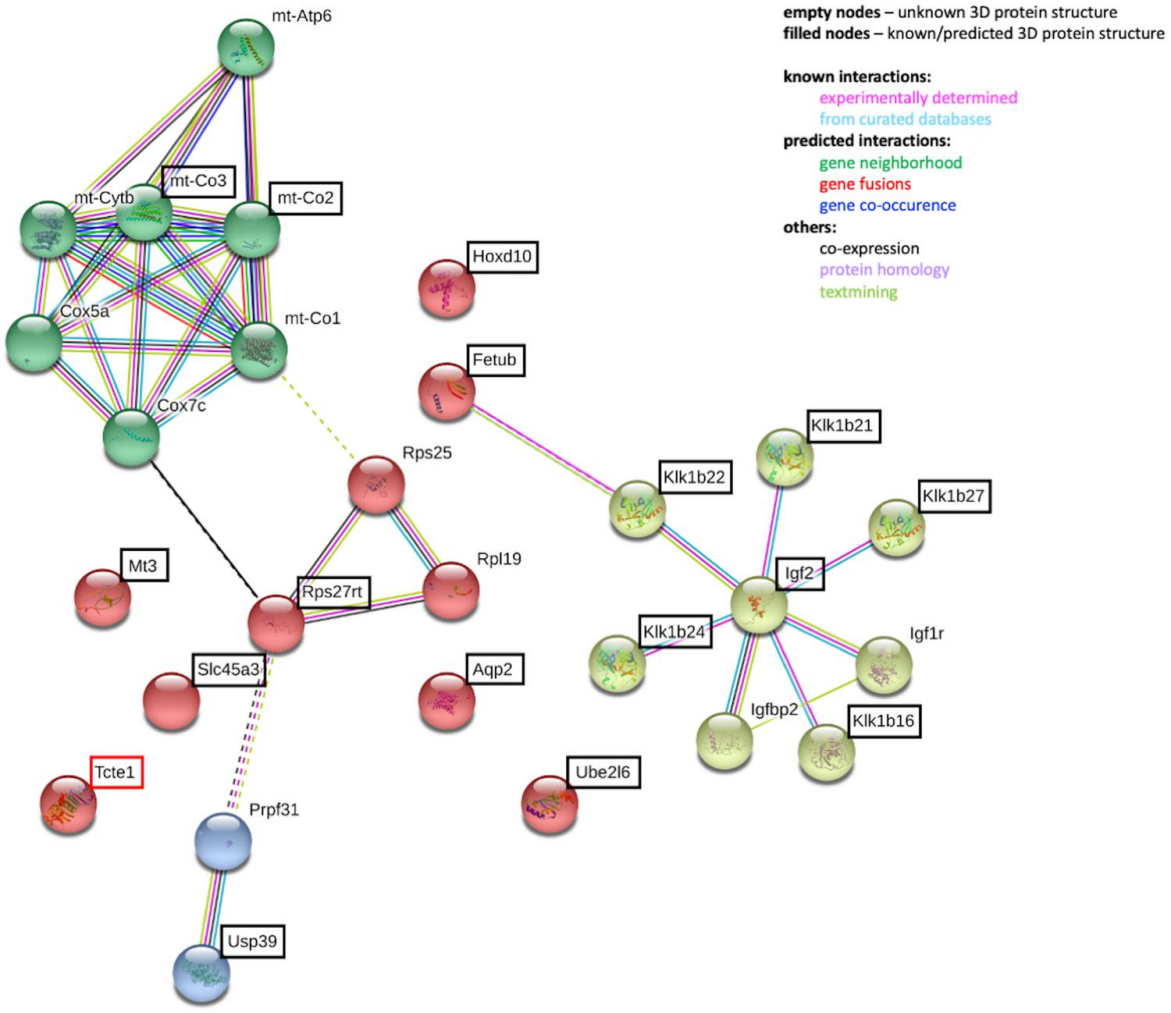
**STRING analyses of potential interactions between proteins with observed differences in gene expression level in RNAseq from mouse testis of the KO model for *Tcte1* gene**. Genes revealed by the RNAseq are bracketed. (STRING database, 24 February 2021).

A two-fold increase for the *Igf2* gene (strongly linked to spermatogenesis, sex determination, testis development, infertility and prostate cancer; Damaschke *et al.*, 2017; Neirijnck *et al.*, 2019; Cannarella *et al.*, 2020; Matsuzaki *et al.*, 2020) has been also observed. Igf2 and Klk proteins are enclosed in a common network consisting of proteins directly linked to fertility (Fig. 8; Supplementary Data File S5 and Fig. S3). One of them is Ambp, a fusion protein, from which proteolysis generates bikunin, a protein whose deficiency results in infertility, due to diminished formation of the stable cumulus–oocyte complex required for maturation and ovulation (Sato and Kan, 2001; Naz and Rajesh, 2005). Bikunin is one of the elements of ITIH domains (Inter-Trypsin Inhibitor Heavy-chain), responsible for hyaluronian metabolism (Salustri *et al.*, 2019; Hull *et al.*, 2021). ITIH is also linked to Plg, which has protease activity and activates metalloproteins, such as Mmp8 and Mmp9, also included in the network. Mmp8 is engaged in degradation and stabilization of occluding and blood–testis barrier (Chen *et al.*, 2018), while Mmp9 (with Igf1 and Igf2) is important for normal placentation (Ghosh *et al.*, 2011). Igf1 and Igf2, are related to Igfbp3, which interacts with humanin expressed in Leydig cells. Humanin together with IGFBP3 and Bax prevents the activation of germ cell apoptosis (Jia *et al.*, 2015; Lue *et al.*, 2021). Importantly, expression of *IGFBP3* is regulated by the *HOXD10* gene (Xue *et al.*, 2013; Pan *et al.*, 2021), whose expression was found to increase by 2.9-fold in our study. *Hoxd10* is involved in prostate cancer processing, cell differentiation and morphogenesis (Mo *et al.*, 2017; Zhang *et al.*, 2019; Jonkers *et al.*, 2020).

Another gene with a 2.2-fold expression rise is *Slc45a3*, a well-known marker of prostate cancer progression, whose loss determines poorer prognosis for cancer patients (Perner *et al.*, 2013; Hernández-Llodrà *et al.* 2017; Pin *et al.*, 2017). Overexpression of another gene identified in our study (a 12-fold rise for the *Usp39* gene) is linked with promotion of prostate or ovarian tumourigenesis. *Usp39* is also involved in pre-mRNA splicing, cytokinesis and spindle checkpoint function, and is crucial for apoptosis (Huang *et al.*, 2016; Zhao *et al.*, 2016; Yuan *et al.*, 2021).

Furthermore, a 2.2–2.5-fold increase for ncRNA genes (*Gm26848*, *Gm45648*; no data concerning their role) and a 2-fold reduction for one pseudogene (*Gm28661*; also no data) were found (Fig. 7). Following the latest data (November 2021) from the Gene NCBI database, Gm45648 has been annotated as having testis-restricted expression in mice, but no function has been suggested. The second ncRNA *Gm26848* found is suggested as an organic cation transporter-like protein across membranes in *Drosophila sechellia*. There were also two other genes with expression changes: a 3.6-fold decrease in pseudogene *Gm44126*, and a 2.5-fold increase in *4933430L12Rik,* which is probably involved in antisense transcription). ‘Gene’ database data showed that *Gm44126* in mice is a gene coding a protein with the function of UDP-glucuronic acid decarboxylase and contains two domains involved in NAD(P)H processing. Thus, it can be suggested that this gene is a novel candidate involved in the mitochondrial respiratory chain. Our data with 3.6-fold expression decrease of this gene seem to fit into the observed diminished functioning of mitochondrial machinery in *Tcte1^−/−^* mice.

Another three genes manifested 2-fold decreases of their testis expression when comparing *Tcte1^−/−^* versus *Tcte1^+−^* (but not to WT): *Aqp2* (aquaporin-2, which is involved in osmotic transportation of water), with well-known roles in spermatogenesis, the female endometrium cycle, and urinary incontinence (Klein *et al.*, 2013; Ramli *et al.*, 2019); *Mt3* (metallothionein-3, committed in homeostasis and detoxification of toxic metals, also in testis) which is important in apoptosis regulation of Bax and Caspase pathways (Honda *et al.*, 2010; Gao *et al.*, 2021)); and *Rps27rt*, a ribosomal protein (Fig. 7; Supplementary Data File S5). It was also found that Rps27rt may interact indirectly with Usp39 and mitochondrial respiratory complex linking mitochondrial energy processing with apoptosis (Supplementary Data File S5). The last gene, *Ube2l6*, was found to have a 2-fold increased expression level in the testis in *Tcte1^+/−^* animals versus *Tcte1^−/−^* and WT. Its role has been established as a ubiquitin enzyme engaged in: stress damage response, cell cycle progression, embryo development, immune response, and factors linked to ATP machinery (Fig. 7) (Huang *et al.*, 2016; Zhao *et al.*, 2016; Sandy *et al.*, 2020; Cai *et al.*, 2021; Yuan *et al.*, 2021). Increased expression of *Ube2l6* is linked to degradation/suppression of cancer cells and affected downstream apoptotic factors (i.e. Caspase 3, Caspase 9, Bcl-2, Bax) (Li *et al.*, 2018).

STRING analyses of potential interactions between proteins with observed differences in gene expression levels in RNAseq in mouse testis of the KO model for the *Tcte1* gene are shown in Fig. 8.

### Immunofluorescence in sperm

**Figure 9.**
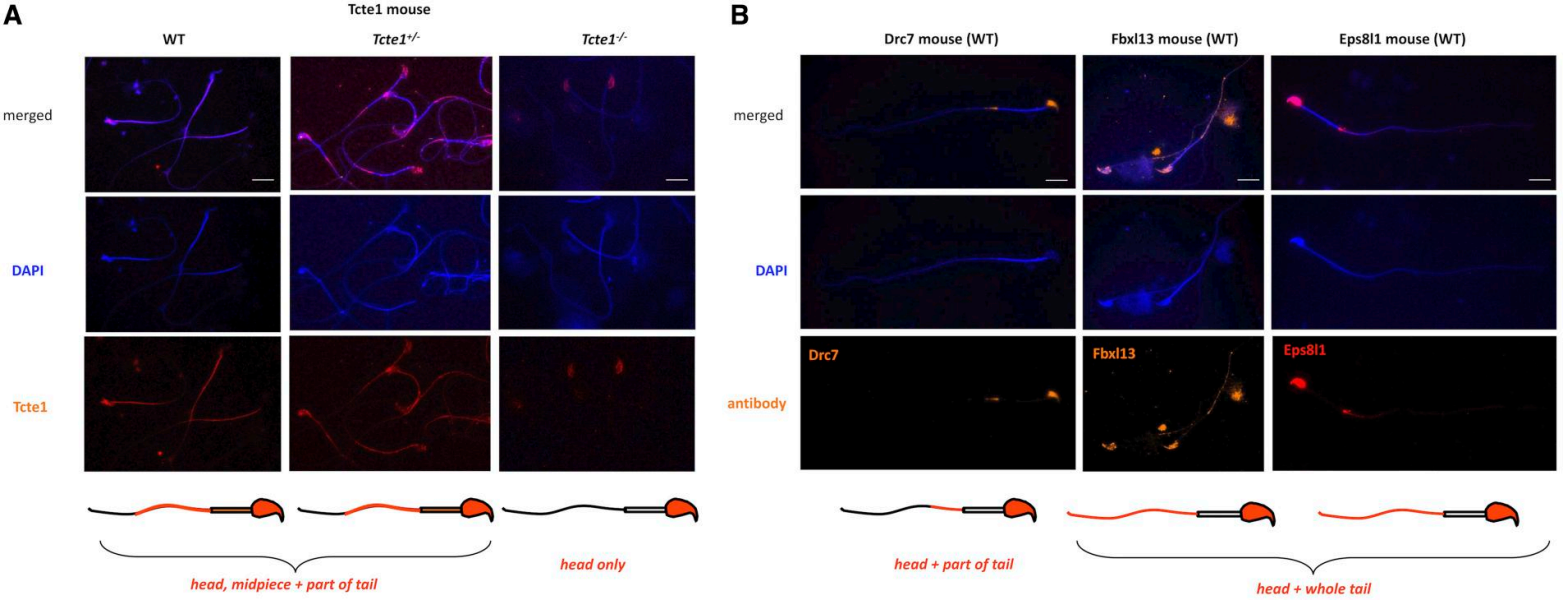
**Immunolocalization of the nexin–dynein regulatory complex (N-DRC) proteins: Tcte1 (Drc5), Drc7, Fbxl13 (Drc6), and Eps8l1 (Drc3) within mouse sperm cells.** (**A**) Localization of Tcte1 in spermatozoa from wild-type (WT), hetero- (^+/−^) and homozygous (^−/−^) animals. (**B**) Localization of other proteins (Drc7, Fbxl13, Eps8l1) building the nexin–dynein regulatory complex (N-DRC), responsible for the coordination of the dynein arm activity and stabilization of the doublet microtubules attachment, in wild-type (WT) spermatozoa. Antibodies: primary (all anti-rabbit 1:100; Biorbyt, Cambridge, UK): Tcte1 (orb357083); Drc7 (orb58695); Fbxl13 (orb678278); Eps8l1 (orb382538); secondary: goat anti-rabbit-AF594 conjugated, 1:500, ab150160, Abcam, Cambridge, UK. Fluorescence microscope: Leica DM5500, filters: DAPI, TxR, FITC, BGR, magnification 630× (with immersion); software: LASX or CytoVision. Bar represents 10 µm.

An example of immunofluorescence *in situ* staining on mouse spermatozoa used to detect the N-DRC proteins has been presented in Fig. 9. WT mouse spermatozoa showed Tcte1 protein presence in the sperm head nucleus, midpiece, and a part of the tail (Fig. 9A). Immunofluorescence signals of Tcte1 in *Tcte1^+/−^* mice were similar to that in WT animals, while in *Tcte1^−/−^* were localized only in the nucleus (Fig. 9A). It seems to confirm that in *Tcte1^−/−^* males, the protein has not been produced properly, revealing only residual amounts in the sperm head nucleus, with no transport to the sperm flagella. The other N-DRC components (Drc7, Fbxl13, and Eps8l1) were localized in the sperm nucleus and among the whole sperm tail (Fbxl13, Eps8l1) or only within its fragment (Drc7), with exception of the midpiece (Fig. 9B). The results obtained seem to suggest co-localization of evaluated N-DRC proteins in various combinations, not common among the midpiece and the terminal part of the sperm tail.

### Sperm ATP levels

**Figure 10.**
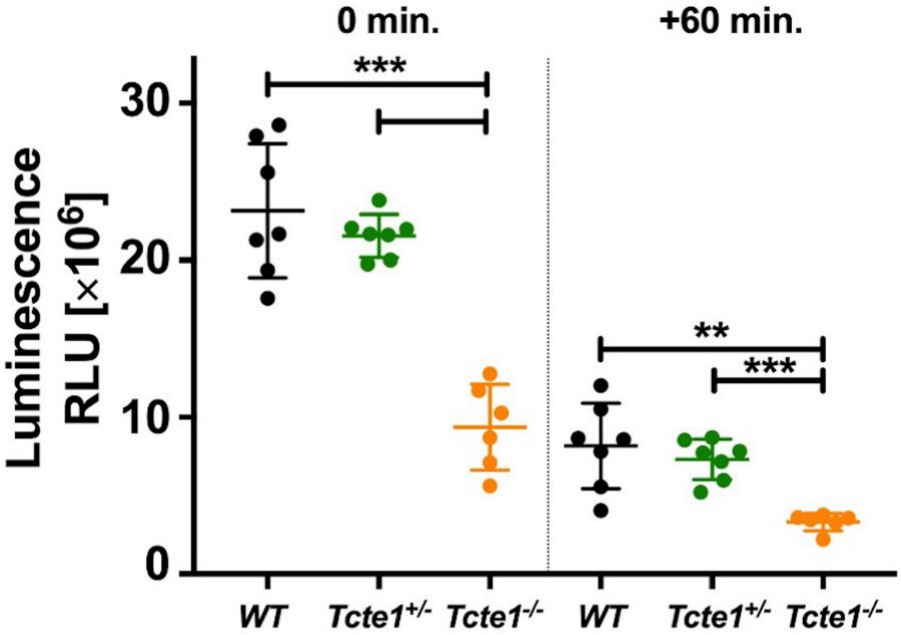
**ATP levels in wild-type (WT), heterozygous (^*+/*^** ^−^ ***)* and homozygous (** ^−^ **^*/*^** ^−^ **) *Tcte1* mice**. Measurements made at two time points (0 and +60 min) showed a decrease of ∼35% in luminescence in all three groups over time. Mean measured values in the homozygous group were ∼2.4-fold lower. Dots: particular animals; long dash: mean value for the group; short dashes, standard deviation; RLU, relative luminescence unit. ***P < 0.0001, **0.0001 < *P* < 0.01.

Measurements performed at two time points (0 and +60 min) revealed that in *Tcte1^−/−^* samples, the luminescence level was ∼2.4-fold lower when compared to WT and *Tcte1^+/−^* mice (*P* < 0.0001) (Fig. 10; Supplementary Table S3). There were no differences between WT and *Tcte1^+/−^* males. Additionally, the decrease in the luminescent signal after 60 min was similar in all three groups studied: ∼35% (WT: 35.27%, *Tcte1^+/−^*: 33.88%, *Tcte1^−/−^* 37.76%).

### Apoptosis in testicular tissue

**Figure 11.**
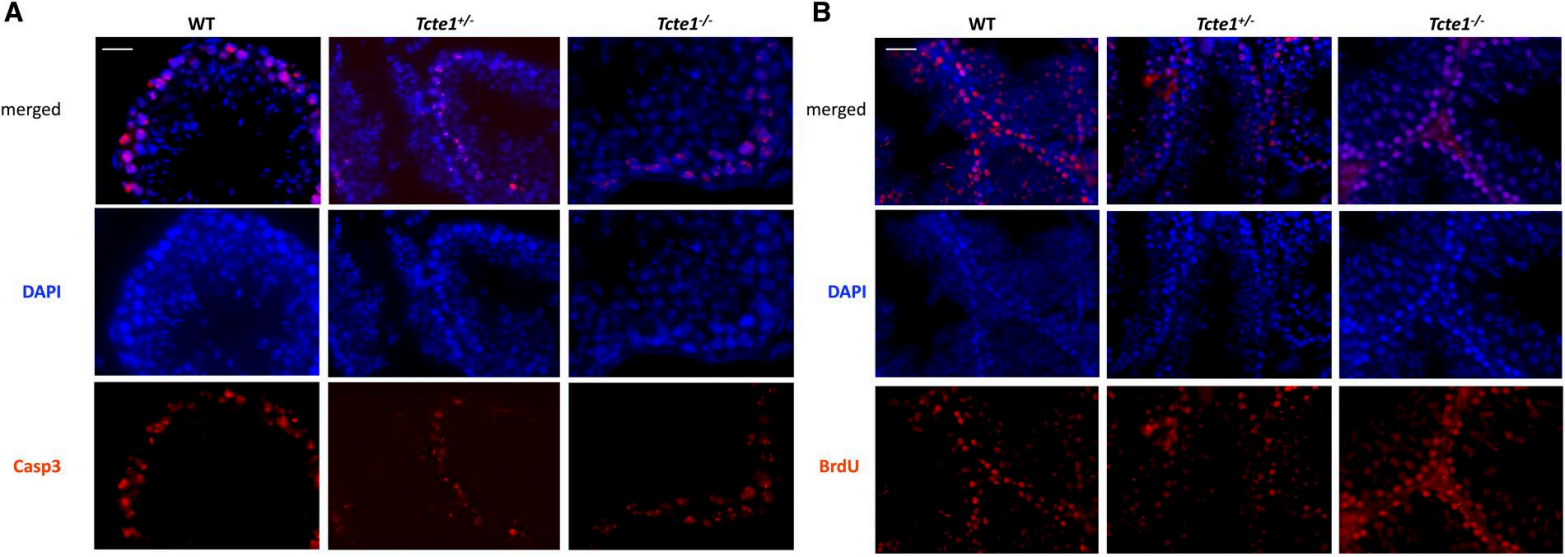
**Immunolocalization of caspase 3 and TUNEL assay results within mouse testicular tissue of the KO *Tcte1* model**. Caspase 3 (**A**) is one of the apoptotic indicators because of its engagement in the induction, transduction, and amplification of intracellular apoptotic signals. Caspase 3 signals were found only in spermatogonia of all three genotypes (wild-type (WT), hetero- (^+/−^), and homozygous (^−/−^)). No differences between the level of fluorescent signals were found between evaluated mouse genotypes, indicating similar levels of apoptosis. Antibodies: primary (1:100) rabbit anti-Casp3 (Biorbyt, Cambridge, UK); secondary (1:500): goat anti-rabbit-AF594 conjugated (ab150160, Abcam, Cambridge, UK). (**B**) TUNEL assay revealed no differences between zygosities (positive BrdU signal). Kit used: ab66110 TUNEL Assay Kit: BrdU-Red; Abcam, Cambridge, UK. Fluorescent microscope: Leica DM5500, filters: DAPI, TxR, magnification 630× (with immersion); software: LASX. Bar represents 5 µm. Analyzed: three independent males per zygosity, on 15–20 tubules each.

Evaluation of the immunofluorescence staining results revealed Casp3-positive signals (indicating apoptosis) in spermatogonia only, in all three mouse genotypes (Fig. 11A). In each zygosity, the numbers of tubules and positively stained spermatogonia (intensity of the signal) were similar, suggesting similar levels of apoptosis. Also, the lack of differences between zygosities was found in TUNEL assays, for all testicular cell types (Fig. 11B). Apoptosis-positive signals were observed in all tubules with both techniques.

### Sequencing: human male samples

There were six potentially associated or important heterozygous SNVs for the *TCTE1* gene found in 15 out of 248 (6.05%) infertile males with drastically decreased semen parameters, ranging from azoospermia to severe oligoasthenozoospermia (Table 1). Three variants were identified with ultrarare allele frequency (MAF ≤0.0005 in the general population; gnomAD) in three males with NOA: c.862C>T (p.Arg288Ter), predicted as a stop-gained mutation leading to non-mediated decay, and thus to protein loss of function with high probability; c.185G>A (p.Arg62His) and c.1048G>A, (p.Gly350Ser), defined as missense ones and probably damaging. The next two SNVs were found as rare missense ones with MAF <0.005: c.397C>T (p.Arg133Cys) (one NOA case) and c.470A>C (p.Glu157Ala) (four NOA cases), and were defined as probably damaging. The last SNV (found in one male with NOA, in one with cryptozoospermia, and in five with severe oligoasthenozoospermia) was not found in the databases, and thus was specified as a novel SNV (c.374T>G; p. Ile125Arg) with missense prediction (Table 1; Supplementary Fig. S4).

**Table 1.** Potentially disease-causing novel and ultrarare heterozygous *TCTE1* variants found in patients with disturbed spermatogenesis.

Sample ID	Phenotype [if available—sperm count: concentration/volume/total//motility (%): a + b/total		Origin	NT change	cDNA position	Prediction	Mutation T@ster	GnomAD	rs	Length of protein	CADD	Position(s) of altered AA [HGVSp]	Polyphen/Sift
**Novel variants**													
P52	SOAs	4.3 × 10^6^/ml/3.0 ml/12.9 × 10^6^//15.0/20.0	Poland	c.374T>G	cDNA.497T>G	Disease causing	Amino acid sequence changed; protein features (might be) affected; splice site changes	Not found	—	Normal	Missense	p.Ile125Arg	Unknown
P88	SOAs	3.4 × 10^6^/ml/1.5 ml/5.1 × 10^6^//2.0/5.0											
P105	SOAs	0.2 × 10^6^/ml/1.5 ml/0.3 × 10^6^//17.0/23.0											
P111	SOAs	3.7 × 10^6^/ml/3.0 ml/11.1 × 10^6^//15.0/20.0											
P161	SOAs	2.3 × 10^6^/ml/3.0 ml/6.90 × 10^6^//10.0/13.0											
P94	Crypto	0.05 × 10^6^/ml/2.0 ml/0.1 × 10^6^//0.0/0.0											
P149	NOA	0.0 × 10^6^/ml/2.0 ml/0.0 × 10^6^//0/0											
**Ultrarare variants (MAF <0.005)**													
U13 No biopsy	NOA	Low volume	USA	c.185G>A	cDNA.308G>A	Disease causing	Amino acid sequence changed	0.00007778	rs201144307	Normal	Missense	p.Arg62His	Probably damaging/deletorious
U17	NOA	Late meiotic arrest	USA	c.397C>T	cDNA.520C>T	Disease causing	Amino acid sequence changed; heterozygous in TGP or ExAC; protein features (might be) affected; splice site changes	0.001722	rs145304614	Normal	Missense	p.Arg133Cys	Probably damaging/deletorious
U50, U57, U117, U235	NOA	Low volume	USA	c.470A>C	cDNA.593A>C	Disease causing	Amino acid sequence changed; heterozygous in TGP or ExAC; protein features (might be) affected; splice site changes	0.002681	rs45591736	Normal	Missense	p.Glu157Ala	Probably damaging/deletorious
U11	NOA	Early meiotic arrest	USA	c.862C>T	cDNA.985C>T	Disease causing	NMD amino acid sequence changed; protein features (might be) affected; splice site changes	0.0002840	rs138414421	NMD	Stop_gained	p.Arg288Ter	Stopgain SNV; PLoF: high confidence
U76	NOA		USA	c.1048G>A	cDNA.1171G>A	Disease causing	Amino acid sequence changed; protein features (might be) affected; splice site changes	0.00006382	rs570334918	Normal	Missense	p.Gly350Ser	Probably damaging/deletorious

Results obtained from the screening of n = 248 participants. Minor allele frequency data obtained from GnomAD v2.1.1.

SOAs, severe oligoasthenozoospermia; NOA, non-obstructive azoospermia; Crypto, cryptozoospermia; NMD, non-mediated decay; NT, nucleotide; AA, amino acid; SNV, single-nucleotide variant; PLoF, loss of function probability; rs, reference SNP accession number; CADD: Combined Annotation Dependent Depletion tool.

### Protein prediction

**Figure 12.**
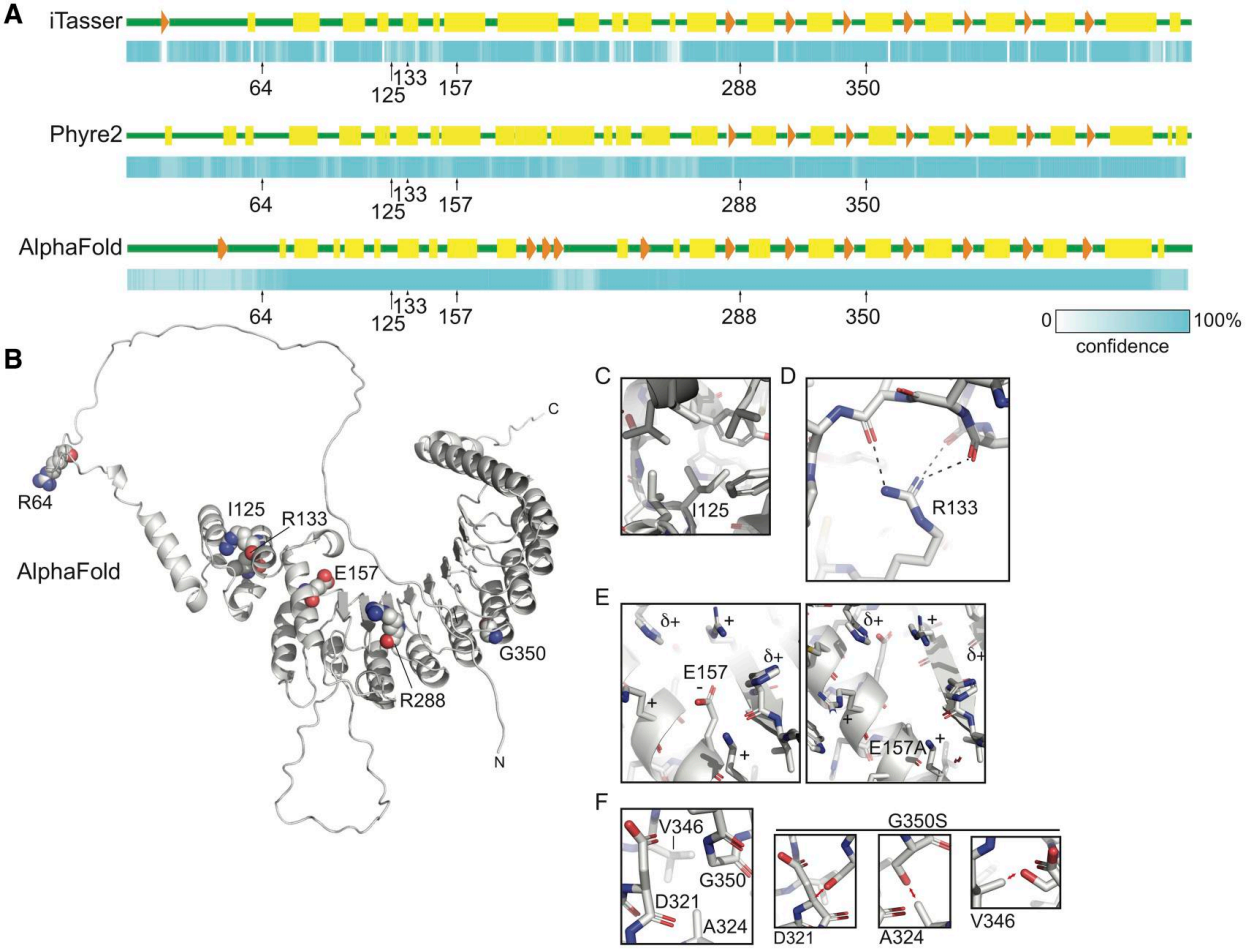
**Homology modeling of TCTE1**. (**A**) Secondary structure diagram and associated confidence scheme generated by I-TASSER (Roy *et al.*, 2010; Yang *et al.*, 2015; Yang and Zhang, 2015], Phyre2 (Kelley *et al.*, 2015], and AlphaFold intensive mode. Positions of amino acids of interest are indicated. Green, predicted unstructured regions; yellow rectangles, predicted helices; orange arrows and arrowheads, predicted strands. The confidence scale ranges from white to cyan, with the probability of the secondary structure increasing with increasing intensity of cyan. (**B**) Cartoon diagram of the homology model of human TCTE1 generated by AlphaFold with residues of interest shown as spheres and labeled. N, amino terminus. C, carboxy terminus. (**C**–**F**) Detailed views of indicated residues and mutations: (C) Ile-125 resides deep within a hydrophobic pocket, which would be disturbed upon substitution with arginine. (D) Mutation R133C would impact the hydrogen bond interactions that organize the architecture of the globular domain N-terminal to the tandem leucine-rich repeats. (E) Mutation of Glu-157 to alanine would destabilize the charge of the surface of this globular region, likely leading to unfolding. (F) Gly-350 allows for the compact interactions of tandem helices of the LRR. Depending on the rotamer it adopted, a serine side chain in this position would sterically clash with residues D321, A324, or V346 in the neighboring helix.

To understand the molecular implications of the identified mutations on the structure and function of TCTE1, we performed homology modeling experiments using the Phyre2, I-TASSER, and AlphaFold tools (Roy *et al.*, 2010; Kelley *et al.*, 2015; Yang *et al.*, 2015; Yang and Zhang, 2015; Tunyasuvunakool *et al.*, 2021). TCTE1 is predicted to contain at least five tandem LRRs in its C-terminus (UniProt Consortium, 2019), while no known three-dimensional protein folds have been assigned to its N-terminus. All homology searches yielded a polypeptide with tandem LRRs with nearly 100% confidence (Fig. 12A; Supplementary Fig. S5). Modeling the N-terminal region of TCTE1 was more difficult; although both Phyre2 and I-TASSER confidently predicted a largely alpha-helical secondary structure for the N-terminal half of TCTE1 (Fig. 12A; Supplementary Fig. S5). Neither tertiary structure was predicted with high confidence; indeed, a comparison of the two demonstrates vastly different folds for the N-terminus from the programs. However, the confidence of the AlphaFold-generated model was high for the region just N-terminal to the LRR. Further, based on the predicted aligned error plot calculated for its models, the confidence of the predicted structure of residues 100–200 was high relative to the position of residues 220–480, providing support for the modeled through-space three-dimensional interactions. That said, we interpreted these models very conservatively.

We attempted to hypothesize how the mutations of interest could affect the tandem LRRs and putative secondary structure of the N-terminus of the protein. Residue R64 resides in a predicted unstructured region and, as such, most likely is solvent exposed in the tertiary structure (Fig. 12A and B). Mutation of this residue from arginine to histidine could therefore affect the surface potential of this disordered region, making it less positively charged. Indeed, this mutation would be expected to impact the interactions that the N-terminus of TCTE1 can have with other molecules, including other members of the N-DRC. The interaction of microtubule-associated proteins, including the N-DRC, with binding partners, is known to be largely charge-based (Kubo and Oda, 2017; Drechsler *et al.*, 2019).

Three mutations of interest occur in the globular region just N-terminal to the LRR. The AlphaFold model clearly shows how mutation I125R would affect the structure of this region (Fig. 12A and C). Residue isoleucine 125 is predicted to be nestled within the hydrophobic core including L97 L109, L112, F91, W131, L197, Y130, and the methyl group of the T121 sidechain. A substitution of positively charged arginine for isoleucine in this position would not be accommodated in this hydrophobic region, likely resulting in local unfolding that could potentially propagate beyond the pocket. Likewise, residue R133 supports the structure of this putative globular domain by forming hydrogen bonds with the backbone of residues 109, 110, and/or 112, thereby possibly contributing to the overall organization of this domain and its positioning relative to the LRR (Fig. 12D). Mutation of this residue to a cysteine would remove the hydrogen bonding groups supporting these interactions, likely leading to a local unfolding that could possibly propagate further. Interestingly, residue E157 is predicted to interact with residues in the LRR, likely stabilizing positively charged residues around it, including several from the linker leading up to the first alpha-strand of the LRR (Fig. 12B and E). Mutation of this residue to alanine would lead to a tertiary destabilization, most likely affecting the overall structure of the N-terminal region of the LRR and the globular region just N-terminal to it.

Two mutations of interest affect the LRR helices. Mutation R288* results in premature truncation, eliminating most of the LRR region from the final protein product, although it is predicted that this premature termination codon would lead to nonsense-mediated decay. Mutation G350S would be expected to result in the destabilization of inter-repeat interactions (Fig. 12F), possibly even unfolding the protein from that position on; the lack of a side chain in glycine allows for the close approach of these tandem structural elements, whereas the addition of the serine side chain would abrogate the tight packing of residues, regardless of which rotamer it adopts. This could result in a restructuring of its C-terminus and/or deforming the surface of the module, therefore affecting its interactions with its binding partners.

Since the N-DRC is conserved from single-celled eukaryotes to mammals, we took advantage of published cryo-electron tomography studies examining the N-DRC from model organism *Chlamydomonas* (Gui *et al.*, 2019). This study was of great interest since it explicitly located the position of the C-terminus of DRC5 with a nanogold particle; importantly, it was consistent with earlier cryo-ET experiments positioning the N-DRC (Heuser *et al.*, 2009). While the resolution of this structure is only 40 Å, it was still useful for placing the modeled TCTE1 structure in the context of the entire N-DRC (Supplementary Fig. S5B). Manual docking of the AlphaFold predicted TCTE1 structure is consistent with the cryo-EM tomography data (Heuser *et al.*, 2009; Gui *et al.*, 2019).

## Discussion

**Figure 13.**
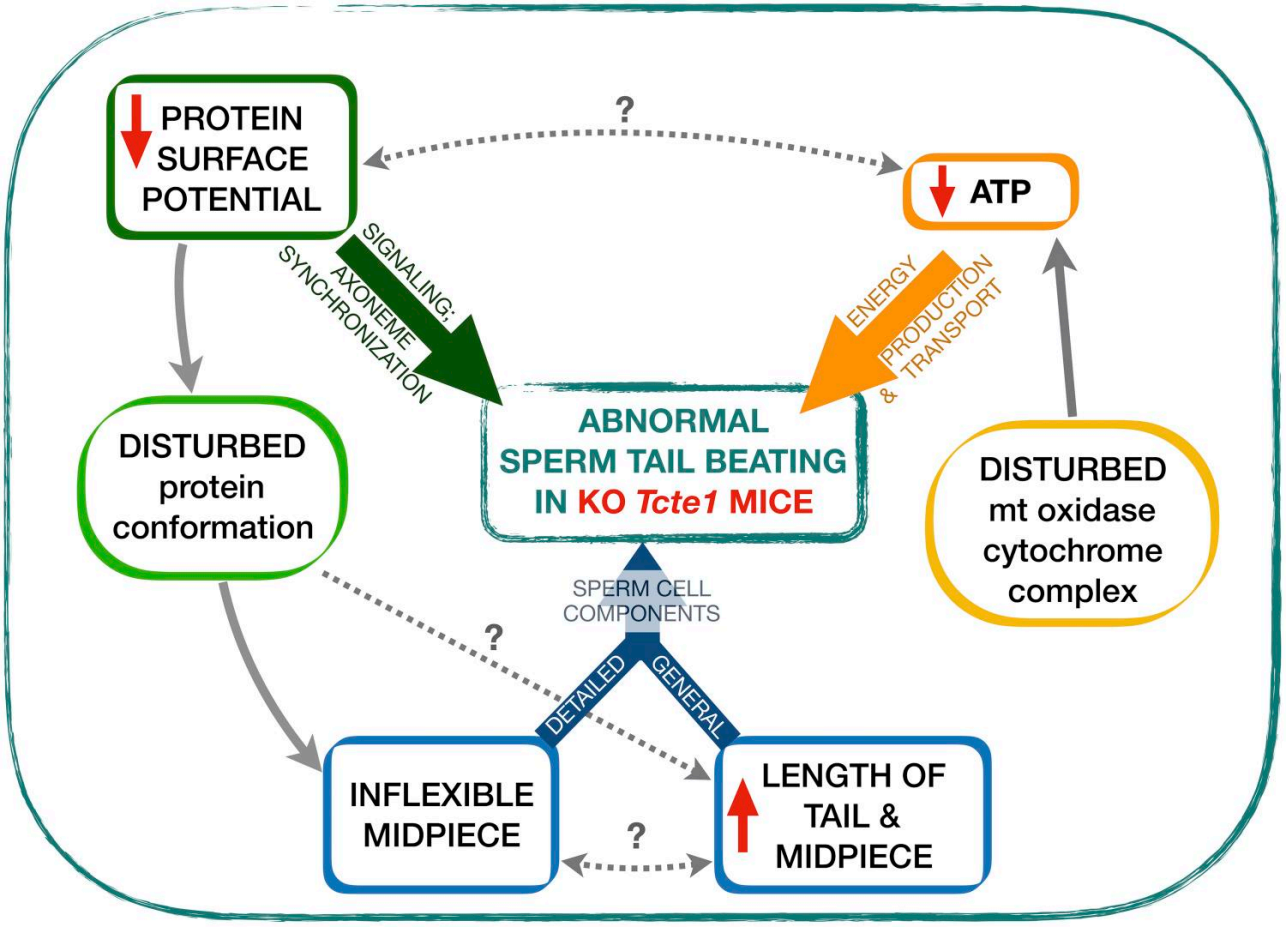
**Summary of findings revealed for abnormal sperm tail beating in the mouse knockout model of the *Tcte1* gene, a component of the N-DRC complex in the sperm tail**. Three major groups of factors required for proper sperm tail beating are marked with bold color arrows (green, yellow, and blue). Documented connections (solid grey arrows) were revealed on the basis of RNAseq data for testis tissues, protein *in silico* predictions for mutated Tcte1 protein, and measurements of ATP level and sperm cell components in mouse spermatozoa. Unresolved possible linkages (cause/effect) are marked with a question (?) mark.

In this study, we have investigated the role of *Tcte1* in male infertility using a mouse knockout model. Using homo- and heterozygous animals, their reproductive potential has been examined, and no progeny were produced by homozygous males. The size of the gonads varied between the zygosity of animals, with two semen phenotypes revealed by sperm quality evaluation. RNAseq analysis of the mutant testicular tissue demonstrated the influence of *Tcte1* mutations on the expression pattern of genes related to mitochondrial cytochrome oxidase activity, apoptosis and spermatogenesis. The ATP level estimates of spermatozoa showed a decrease in homozygous males. Additionally, protein prediction modeling of identified variants in human samples revealed changes in the protein surface charge potential, leading to disruption in helix flexibility or dynamics, and thus, suggesting a disrupted TCTE1 interaction with its binding partners within the axoneme. The major findings concerning observed abnormal sperm tail beating in KO *Tcte1* mice are summarized in Fig. 13. We have also identified and discussed novel functions of genes with testicular expression significantly disturbed in the *Tcte1* knockout. Therefore, this study contains novel and comprehensive data concerning the role of *TCTE1* in male infertility.

### Phenotypes of homozygotes and heterozygotes

There was only one previously published study concerning a knockout mouse model of *Tcte1* (Castaneda e*t al.*, 2017). It was shown that the expression of *Tcte1* starts at the stage of early haploid round spermatids and continues into the elongated spermatids. The authors showed the infertility of homozygous males due to asthenozoospermia derived from limited and circular flagellar beating, followed by fertile heterozygotes. The study did not find any differences between homozygous and WT males in the testicular histology, followed by similar testis weight, sperm concentration, and sperm morphology (Castaneda *et al.*, 2017). We also did not find any structural differences in the testicular and epididymal tissue histology. However, the weight of the testes decreased significantly (∼41% in *Tcte1^−/−^*, 7% in *Tcte1^+/−^*), followed also by testis size reduction (by 22%) and lowered germ cell layer thickness in *Tcte1^−/−^* animals (Fig. 4B and C). When comparing semen parameters (Fig. 6A–C), we found an enormous decline in sperm concentration in both *Tcte1^−^* (3.3-fold) or *Tcte1^+/−^* (2.5-fold) males, and a double increase in abnormal sperm morphology in *Tcte1^−/−^* animals. Similar to Castaneda *et al.* (2017), we also documented the impaired sperm motility representing a circular mode of the sperm tail beating. In addition, the increased ratio of immotile spermatozoa in homozygous males was documented in our study. We can suggest that these discrepancies may result from the various number of animals used for evaluation: three males in the study of Castaneda *et al.*, (2017) versus at least 18 per each zygosity in our study when comparing semen parameters and tissue characteristics (except for sperm morphology evaluation with at least 5 animals used). Furthermore, the differences in knockout construction used could also be causative for the observed variances: reporter-tagged insertion with conditional potential versus the exon 3 deletion. Thus, we have observed two various phenotypes dependent on the zygosity of the animals: homozygous males manifested oligoasthenoteratozoospermia (OAT) and were infertile, while heterozygous males demonstrated oligozoospermia (O) and remained fertile. Data collected in the International Mouse Phenotypic Consortium (IMPC; a repository of all available mouse phenotypes created, followed by the possible linkage to human disease defects; https://www.mousephenotype.org), concern only one *Tcte1* mouse study (Castaneda *et al.*, 2017), while we reveal an unmatched phenotype of decreased sperm count.

We suggest that a similar effect, decreased sperm count linked to *TCTE1* mutations, could be observed in humans. Our pilot mutation screening of 248 human infertile males with severely decreased sperm count (from oligo-, via crypto-, to azoospermia) revealed six heterogeneous SNVs (one novel, three ultrarare, and two rare; all predicted as disease-causing) with a total frequency of 6.05% (Table 1). We propose that molecular epidemiology studies of infertile patients should reveal the existence of homozygous or compound heterozygous *TCTE1* variants with clear direct downgrading effects on spermatogenesis. However, we claim that also heterozygous variants can be causative, similar to *SPINK2* whose deficiency has been documented as responsible for oligoasthenozoospermia in heterozygous mice versus azoospermia in homozygous animals, as well as azoospermia in human homozygous brothers versus their heterozygous father with oligozoospermia (Kherraf *et al.*, 2017). It can be explained by the probable occurrence of haploinsufficiency; when the single copy of the WT allele is in a heterozygous combination, the variant allele is insufficient to produce the WT phenotype. Our observations, in addition to the *SPINK2* data and data related to infertility (i.e. P1/P2 protamines, *Pde8b*, *Trim28*, *Tcfl5*), and to the other diseases (i.e. *PIKFYVE* in cataract, *NFIA*, *SCN1A* in neurological or epilepsy disturbances, *KLF1* in hemoglobin persistence, *NR5A2* in pancreatic cancer), seem to confirm that the severity of the phenotype depends on the gene expression level (Jodar and Oliva, 2014; Tan *et al.*, 2020; Leal *et al.*, 2021; Sandhu *et al.*, 2021; Heshusius *et al.*, 2022; Mei *et al.*, 2022; Ogura *et al.*, 2022; Valassina *et al.*, 2022; Xu *et al.*, 2022). We have documented variable phenotypes for a novel *TCTE1* variant c.374T>G (p.Ile125Arg) that has been documented in seven males, with azoospermia, cryptozoospermia, or severe oligoasthenozoospermia. Thus, it seems to suggest the reasonable candidacy of haploinsufficiency as the mechanism responsible for the observed phenotypic spectra also in the case of the *TCTE1* gene. There is only one study available documenting *TCTE1^−/−^* in a human case, so far (Zhou *et al.*, 2022). Among 130 asthenozoospermic patients, the authors found a frameshift mutation c.396-397insTC (p.Arg133Serfs*33) in one of them that resulted in the premature translational arrest of the forming peptide. Successful delivery was possible only after *in vitro* fertilization (IVF). Importantly, the authors also checked the IVF rate in homozygous mice but showed no pregnancy success. The unresolved question was whether homozygous *TCTE1* mutation in humans causes only asthenozoospermia or rather the semen quality phenotype is mixed, including also abnormal morphology, but this was not evaluated by the authors.

### Kallikreins, aquaporin 2

Our pathway analysis revealed a network including two genes with changed expression level in testis: *Igf2* (2-fold increased) and *Klk1b22* (8-fold decreased), supported by an interesting set of proteins linked to apoptosis and cellular processing (Figs 7 and 8; Supplementary Data File S5 and Fig. S3). We can suggest that the observed *Igf2* overexpression in our study can be one of the associated factors leading to decreased sperm count and testis parameters in the evaluated KO mice model because of its known relation to spermatogenesis and testis development (Damaschke *et al.*, 2017; Neirijnck *et al.*, 2019; Cannarella *et al.*, 2020; Matsuzaki *et al.*, 2020). Documented reductions can be supported by the observed constrained expression of *Klk1b22*; kallikrein is mostly known for its neuronal activity; however, it cannot be excluded that we have revealed an additional role of this protein, similar to the other members of the kallikrein family with roles in semen liquefaction and degradation of extracellular matrix proteins in the interstitial area surrounding Leydig cells of the adult mouse testes (*Klk1b27*, *Klk1b21*, *Klk1b24*) (Fink *et al.*, 2007; Marques *et al.*, 2016, Du Plessis *et al.*, 2013; Michaelis *et al.* 2017). Notably, *Klk1b22* expression is significantly enriched in testis. Expression of all four kallikreins was decreased by 2.5 to 8-fold in our *Tcte1* KO model, indicating their participation in the reduction of the sperm parameters measured (Fig. 5).

We have also documented a 2-fold decrease in the expression of *Aqp2* (Aquaporin 2), which is involved in fluid resorption and water movement within the duct system, with a known role in spermatogenesis (Klein *et al.*, 2013; Ramli *et al.*, 2019). It was documented that in stallions, Aqp2 is expressed in Leydig cells, round and elongated spermatids (Klein *et al.*, 2013). In addition, this protein works within a network of molecules that are linked to cell membrane trafficking (i.e. Rab11a, Myo5b), followed by activity of protein kinases (Prkacb, Prkaca) that are known as regulators of cell proliferation, chromatin condensation/decondensation, and nuclear envelope disassembly/reassembly, and all is supported by ATP metabolism (Hspa8) (Supplementary Data File S5) (Markou *et al.*, 2022; Wagner *et al.*, 2022). Considering the literature data, we suggest Aqp2 plays an important role in the reduction of the spermatid volume during spermatogenesis and in proper sperm morphology assessment and the observed morphological differences between *Tcte1* mice zygosities in our study. However, such measurements require high-resolution microscopy, thus, we can only speculate the Aqp2 role in our model.

### Disruptions in sperm cell structure

The TCTE1 protein contains five LRRs that are known to be directly involved in receptor-mediated signaling on a structural basis for interaction between proteins (Kobe and Kajava 2001; Ng *et al.*, 2011). It was documented that variants in genes encoding LRRs are commonly implicated in the pathogenesis of more than 60 human diseases (Ng *et al.*, 2011; Matsushima *et al.*, 2019). LRR mutations mostly occur within regions that are protective for the hydrophobic core of the LRR domains. Thus, mutations in LRRs may affect the charge of the surface potential, leading to conformational changes of the protein or induction of protein cumulation. Our prediction model of potential changes in the TCTE1 protein resulting from mutations found in human samples seems to point out the prospective impact on the interactions of the TCTE1 with other molecules (binding partners) known to be largely charge-based, including N-DRC elements or microtubule-associated proteins (Kubo and Oda, 2017; Drechsler *et al.*, 2019). In the case of the TCTE1 protein, observed amino acid substitutions promote the abnormal aggregation of the TCTE1 protein, and decrease its structural stability, probably leading to the observed disturbances. The beating motion of flagella is maintained by an electrostatic cross-bridge between negatively charged tubulins and positively charged N-DRC (Kubo and Oda, 2017). Thus, changes in the charge potential of the TCTE1 surface, which is one of the N-DRC components, seem to provide modified (reduced) flagellar beating, similar to that with mutations in another N-DRC element, the DRC4 protein, where changes in charge were essential for proper flagellar motility (Kubo and Oda, 2017; Lewis *et al.*, 2016). Also, the inflexibility of the midpiece observed in spermatozoa of *Tcte1* knockout mice seems to be a consequence of the changed structure of LRRs in the Tcte1 protein.

It is also known that the lengths of the sperm midpiece and tail may be crucial in the determination of sperm swimming velocity, which is then followed by a decreased fertilization rate (Firman and Simmons, 2010; Gu *et al.*, 2019). Available data show that sperm with a shorter midpiece may swim slowly and are observed in asthenozoospermic cases (Anderson and Dixson, 2002; Firman and Simmons, 2010; Gu *et al.*, 2019). A meta-analysis of sperm shape features according to sperm competition theory showed that, in the majority of vertebrates, a longer sperm cell (including midpiece or flagellum) has been attributed to faster swimming and that the sperm size may be increased when sperm number is lowered (Lüpold *et al.*, 2020). In our study, we have documented longer sperm tails and midpieces in *Tcte1^−/−^* animals that, in fact, were infertile. Thus, we can suggest that: (i) the observed longer sperm tails and midpieces may be co-responsible for the aberrant motility observed, and (ii) the documented longer sperm tails and midpieces may be an indirect result of the constrained sperm count observed, because of some sort of compensation for aberrant motility. However, this issue remains only a suggestion because of restricted literature data relative to similar observations. Bennison *et al.* (2016) revealed that, in a passerine bird, sperm tail velocity was correlated to total sperm length, but only up to a certain point of the length value, at which the velocity started to decrease. On the other hand, Gu *et al.* (2019) showed that in mammalian species, there were some changes in the axonemal slopes when the flagellar length increased. The results obtained in our study and supported by other research concerning sperm tail-beating pathways should be a good starting point for the creation of further mathematical computations for a better understanding of asthenozoospermia.

### Constrained energy metabolism

Flagellar motility is determined by the coordinated activity of asymmetrically distributed dyneins in the axoneme. The driving factor for this activity is the hydrolysis of ATP released from the OXPHOS system (Mitochondrial Oxidative Phosphorylation System) (Ford 2006; Tourmente *et al.*, 2015). ATP is being produced in mitochondria placed within the sperm midpiece. In our study, ATP level measurements revealed its decreased levels in sperm cells of *Tcte1^−/−^* mice, which can result in faulty dynein functioning, leading to asthenozoospermia via abnormal sperm tail beating. We have documented that *Tcte1* KO resulted in significant changes in the expression level of testicular genes involved in mitochondrial energy transport. That leads to the conclusion that homozygous *Tcte1* mutation results not only in an inflexible midpiece leading to asymmetric circular motility, as presented in this study and by others (Fung *et al.*, 2018), and then to asthenozoospermia, but also to aberrant functioning of mitochondrial OXPHOS machinery leading to the disruption of ATP production. Thus, the sperm tail has also insufficient power for beating because of the decreased ATP content. Castaneda *et al.* (2017) suggested that the reason for the observed ATP decrease may result from disturbances in a glycolytic pathway. It is already known that glycolytic energy is enough for spermatozoa to survive (Nascimento *et al.*, 2008; Zhang *et al.*, 2022). However, for sperm motility and then fertilization, OXPHOS energy production is required (Ford 2006; Tourmente *et al.*, 2015; Zhang *et al.*, 2022). In our study, RNAseq results clearly revealed the influence of the *Tcte1^−/−^* mutation on genes involved directly in the electron transport chain, such as *mt-Co2* and *mt-Co3*, crucial components of the respiratory chain complex IV, one of the OXPHOS elements. Observed reduced expression seems to clearly demonstrate decreased ATP production via disturbed electron chain transportation, and the reduction in the sperm motility observed in *Tcte1^−/−^* males, leading to the observed asthenozoospermia. Detailed STRING analysis revealed definite interactions between both listed proteins identified in our study, and a list of other mitochondrial ones (mt-Co1, mt-Cytb, Cox5a, Cox6a, Cox7c, mt-Atp6; Supplementary Data File S5 and Fig. S3) clearly related to proper processing of cytochrome energetic activities (Ganetzky *et al.*, 2019; Brischigliaro and Zeviani, 2021). Overexpression or mutations in *COX1*, *COX2*, and *COX3* were previously observed in infertile men with varicocele and OAT, or with diabetes (Perrotta *et al.*, 2012; Heidari *et al.*, 2016; Mostafa *et al.*, 2016). Additionally, it was shown that the system of COX2 and prostaglandins plays a key role in the development of the male gonad, spermatogenesis, and steroidogenesis, especially in the functioning of Sertoli and Leydig cells, and was also found to be significant in the background of idiopathic infertility status (Frungieri *et al.*, 2015). It seems that, in our study, the reason for the observed disturbed motility, represented as circular movement of spermatozoa, supported by increased ratio of immotile sperm cells in *Tcte^−/−^* mice, leading to asthenozoospermia, was a result of two factors: (i) inflexibility of the sperm midpiece resulting from a changed protein charge level in the Tcte1 protein, followed by elevated lengths of the sperm tail and midpiece, and (ii) the decreased amount of ATP produced, with an inability to get sufficient energy for proper sperm motility.

### Protection and/or compensation?

We have also documented an important role of the *Fetub* gene for the first time in the male testis. Besides its known role in female fertility (Bansho *et al.*, 2017; Karmilin *et al.*, 2019), a detailed later study suggested a role for it in the context of prostate cancer (Zhan *et al.*, 2020). It was found that overexpression of *FETUB* increases the apoptosis of prostate cancer cells, followed by the inhibition of their proliferation, migration, and invasion, and also slows the growth of tumours when compared to controls (Zhan *et al.*, 2020). Thus, in the overexpression mode, Fetub plays a protective role for the prostate. It seems to explain the results obtained in our study, where *Fetub* expression was increased almost 5-fold in *Tcte1^−/−^* mice, but no tumor signs were observed in the mouse testis at the tissue level. Zhan *et al.* (2020) found a connection between *Fetub* overexpression, and induction of Bax (Bcl-2-associated X protein) and caspases, resulting in the promotion of apoptosis. It is known that Bax/Bcl-2 induction/inhibition is directly related to the release of cytochrome c (Cyt c) in caspase-dependent signaling (Dadsena *et al.*, 2021; Li *et al.*, 2021; Samaiya *et al.*, 2021). Cytochrome c is one of the two electron carriers in the electron transport chain responsible for transport directly to the respiratory chain complex IV (Alvarez-Paggi *et al.*, 2017; Yin and O’Neil 2021). Due to Cyt c release, complex IV is essential for apoptosis and cell fate (Li *et al.*, 2021; Samaiya *et al.*, 2021). Following all these factors, our results seem to reveal a connection between *Fetub* and diminished mitochondrial chain IV activity in mice testis, which has not been reported so far. It can be also supported by the known relationship between FETUB and the inactivation of the PI3K/AKT signaling pathway (a key player in the regulation of cell proliferation and apoptosis in cancerogenesis), in which Bax/Bcl-2 is involved (Zhan *et al.*, 2020).

The novel testis-protective role in our study can be also credited to three other genes with increased expression levels in testicular tissue: *Hoxd10* (3-fold increase), *Slc45a3* (2.2-fold), and *Ube2l* (2-fold). Overexpression/activation of *HOXD10* has already been documented as an inhibitor of tumorigenesis (Mo *et al.*, 2017; Zhang *et al.*, 2019; Jonkers *et al.*, 2020). Expression of *Slc45a3*, also known as ‘prostein’, has been documented as weaker in cancer tissues (Perner *et al.*, 2013; Hernández-Llodrà *et al.* 2017; Pin *et al.*, 2017). An elevated expression level of *Ube2l* is known to be connected with the degradation or suppression of tumor cells (Li *et al.*, 2018). On the other hand, a 12-fold increased gene expression level was observed for *Usp39* (ubiquitin specific peptidase 39), an oncogenic splicing factor whose expression is positively linked with cancerogenesis in prostate or ovaries, while its silencing has been shown to induce apoptosis and cell cycle arrest (Huang *et al.*, 2016; Zhao *et al.*, 2016; Yuan *et al.*, 2021).

Taking together data obtained for the genes related to cancerogenesis and/or apoptosis described above, we can hypothesize that we observe an interesting novel network of interactions between two groups of genes that are reciprocally neutralizing the potential influence on the testis tissue. Mainly, in the first group, we have genes with a suggested novel anti-cancer role in the testis (*Fetub*, *Hoxd10*, *Slc45a3*, *Ube2l6*), with increased expression levels inhibiting cancer cell development, supported by the protective role of *Mt3*; while the *Usp39* gene, with elevated expression levels (the most of all the observed changes), should lead to some tumor-like changes within the testis tissue. Following the fact that both histological evaluation, as well as apoptosis stainings (Fig. 11), showed no differences between heterozygous and homozygous mutant mice, our hypothesis seems to be justifiable. On the other hand, there is a prediction that many of the genes (especially so-called cancer-testis-associated antigens, CTA) related to spermatogenesis may function in tumorigenesis (CheNg *et al.*, 2011; Bode *et al.*, 2014; Wang *et al.*, 2016; Babatunde *et al.*, 2017; Nagirnaja *et al.*, 2018; Yang *et al.*, 2020; Aurrière *et al.*, 2021). Due to known similarities between oncogenic and spermatogenic processes, especially in energy metabolism, we can carefully hypothesize that in our KO model, the genes with changed expression profiles, and known to be highly involved in tumorigenesis, may also be important in spermatogenesis. Obviously, there is a need for further studies in this direction to confirm these suggestions or to reveal the other (still unknown) mechanisms.

## Conclusions

We have documented that the *TCTE1* gene should be added to the ‘male infertility list’ of molecular background because of its crucial role in spermatogenesis and proper sperm functioning. The mutations in the mouse *Tcte1* knockout model revealed two phenotypes, depending on zygosity with infertile oligoasthenoteratozoospermic homozygotes and fertile oligozoospermic heterozygotic males, suggesting the involvement of a haploinsufficiency mechanism. All collected data indicate that the Tcte1 protein is functioning in spermatogenesis and show how its mutations add a level of complexity to the clinical manifestation of decreased semen parameters. The disrupted energy machinery of the spermatozoa, followed by newly suggested roles of genes related to cancerogenesis and/or apoptosis (here in the context of their neutralizing or compensative effect on the testicular tissue), seems to underline the complex and devastating effect of the single-gene mutation on a variety of testicular molecular networks, leading to reproductive failure. Further advanced studies are required to reveal the details of the mechanisms responsible for the molecular pathways of the interactions between Tcte1 (the structural protein) and other non-structural networks whose expression levels were changed remarkably in this study.

## Supplementary Material

hoae020_Supplementary_Data

## Data Availability

All data generated or analyzed during this study are included in this published article and its supplemental information files. RNAseq data are available in GEO database (https://www.ncbi.nlm.nih.gov/geo/) under the accession number: GSE207805. The results described in the publication are based on whole-genome or exome sequencing data which includes sensitive information in the form of patient-specific germline variants. Information regarding such variants must not be shared publicly following the European Union legislation described in the following document: https://publications.jrc.ec.europa.eu/repository/bitstream/JRC113479/policy_report_-_review_of_eu_national_legislation_on_genomics_-_with_identifiers_1.pdf, therefore the access to raw data that support findings of this study are available from the corresponding author upon reasonable request.
