# Supplementary material for: Effects of Tcte1 knockout on energy chain transportation and spermatogenesis: implications for male infertility
Source: Hum Reprod Open. 2024 Apr 4;2024(2):hoae020. doi: 10.1093/hropen/hoae020 (PMC11035007; doi:10.1093/hropen/hoae020)
Supplement: hoae020_Supplementary_Data [file hoae020_supplementary_data.zip › Supplementary Tables S1-S3.pdf]

Supplementary Table S1: Reproductive potential of KO Tcte1 mice.

	mated pairs	pairs with success	pairs w/o success	mean time to get F4 (weeks)	mean time from mating to 1st pedigree (days)	time from mating to 1st pedigree (range/days)	mean time between litters (days)	mean time between litters (range/days)	no. of live litters	no. of litters/ sucessed pair	no.of "0" litters	pups/"0" litter	range	no. of all pups	pups/live litter	range	no. of males (% of all pups)	males/litter	range	no. of ho males (% of all males)	ho males/litter	range	no. of het males (% of all males)	het males/litter	range
♂ *Tcte1*$^{-/-}$ × ♀WT	4	0	4	na	na	na	na	na	na	na	na	na	na	na	na	na	na	na	na	na	na	na	na	na	na
♂ *Tcte1*$^{+/-}$ × ♀*Tcte1*$^{+/-}$	5	5	0	17.2 (12-22)	39.8 ± 12.21	25-50	33.7 ± 8.40	21-50	14	2.8	2	1.50	1-3	109	7.85 ± 0.54	1-10	55 (50.46%)	3.88 ± 0.89	1-7	17 (33.25%)	1.23 ± 0.33	0-3	27 (48.75%)	1.90 ± 0.74	0-4
♂ *Tcte1*$^{+/-}$ × ♀WT	3	3	0	14.0 (12-16)	29.67 ± 1.53	28-31	38.44 ± 12.61	21-75	7	2.33	2	na	na	58	8.22 ± 0.63	7-10	32 (55.17%)	4.67 ± 1.16	1-7	0 (0%)	0.00	0	16 (50%)	2.33 ± 0.58	1-4

	no. of wt males (% of all males)	wt males/litter	range	no. of females (% of all pups)	females/litter	range	no. of ho females (% of all females)	ho females/litter	range	no. of het females (% of all females)	het females/litter	range	no. of wt females (% of all females)	wt females/litter	range	m:f	mho: mhet	mho: mwt	mhet: mwt	mho: fho	mhet: fhet	mwt: fwt	fho: fhet	fho: fwt	fhet: fwt
♂ *Tcte1*$^{-/-}$ × ♀ WT	na	na	na	na	na	na	na	na	na	na	na	na	na	na	na	na	na	na	na	na	na	na	na	na	na
♂ *Tcte1*$^{+/-}$ × ♀*Tcte1*$^{+/-}$	11 (18%)	0.75 ± 0.49	0-3	54 (49.53%)	3.97 ± 1.07	1-8	14 (24.67%)	1.08 ± 0.77	0-4	33 (60.92%)	2.42 ± 0.50	0-5	7 (15.75%)	0.70 ± 0.57	0-3	1.02	0.63	1.55	2.45	1.21	0.82	1.57	0.42	2.00	4.71
♂ *Tcte1*$^{+/-}$ × ♀ WT	16 (50%)	2.33 ± 0.58	0-4	26 (42.26%)	3.56 ± 1.78	1-6	0 (0%)	0.00	0	17 (58.47%)	2.33 ± 1.61	0-4	9 (41.53%)	1.22 ± 0.39	0-2	1.23	0.00	0.00	1.00	0.00	0.94	1.78	0.00	0.00	1.89

Mating combinations and their results including: time of breeding, number of litters and pups, number of male and female pups, and genotypes observed in pups of each combination.
*** $p<0.0001$, ** $0.0001< p < 0.01$, * $0.01 < p < 0.05$

Supplementary Table S2: Characteristics of antibodies used for N-DRC complex staining in mouse sperm.

Antibody	Cat. no.	Host/reactivity	Immunogen/target	UniProt ID	Human antigen sequence identity to the mouse ortholog
TCTE1 (DRC5)	orb357083 (biorbyt)	Host: rabbit (polyclonal)<br>Reactivity: human	Recombinant Human T-complex-associated testis-expressed protein 1 protein (285-501AA)	Human: Q5JU00<br>Mouse: A6H639	92%
DRC7	orb58695 (biorbyt)	Host: rabbit (polyclonal)<br>Reactivity: human, mouse, rat, bovine, canine, guinea pig	synthetic peptide directed towards the C-terminal region of Human CCDC135	Human: Q8IY82<br>Mouse: Q6V3W6	98%
FBXL13 (DRC6)	orb678278 (biorbyt)	Host: rabbit (polyclonal)<br>Reactivity: human, mouse, rat	Immunogen sequence region: 358-707aa	Human: Q8NEE6<br>Mouse: Q8CDU4	79%
EPS8L1 (DRC3)	orb382538 (biorbyt)	Host: rabbit (polyclonal)<br>Reactivity: human, mouse, rat	KLH-conjugated synthetic peptide encompassing a sequence within the center region of human EPS8L1	Human: Q8TE68<br>Mouse: Q8R5F8	93%

Human antigen sequence identity between human and mouse orthologs was also shown (blast comparison; information from Biorbyt producer).

Supplementary Table S3: ATP measurement data obtained for KO Tcte1 male mice.

		mice ID - wt							RLU
**measurement**	**replicates**	**1693**	**1720**	**1721**	**1730**	**1681**	**1682**	**1683**	
1st (0 min.)	a	28388802	21122622	19100922	17468506	28139908	21700056	25668440	
	b	28380046	21324386	19457374	17629914	28081684	21744034	25615467	
	c	29016734	21420028	19552152	17658638	27529972	21576932	25434664	
	mean	***28595194***	***21289012***	***19370149***	***17585686***	***27917188***	***21673674***	***25572857***	***23143394,33***
2nd (+60 min.)	a	8510890	8572207	7704007	5511118	4001735	10346051	11997899	
	b	8572993	8668582	7833232	5565773	4035294	10593685	12032172	
	c	8612547	8698597	7860539	5592265	4052669	10609660	12045947	
	mean	***8565477***	***8646462***	***7799259***	***5556385***	***4029899***	***10516465***	***12025339***	***8162755,333***
decrease between 1st and 2nd measurement [%]									35,27034633

		mice ID - het							RLU
**measurement**	**replicates**	**1690**	**1691**	**1694**	**1719**	**1729**	**1739**	**1740**	
1st (0 min.)	a	19576760	21816762	21446012	19888686	21738396	21443152	23664120	
	b	19834880	22136644	21628246	20034788	22036918	21725480	23828796	
	c	19820430	22221754	21744642	20098396	22105804	21806154	23908434	
	mean	***19744023,3***	***22058386,7***	***21606300***	***20007290***	***21960372,7***	***21658262***	***23800450***	***21547869,24***
2nd (+60 min.)	a	7087197	8519749	8434987	7674274	5133504	5933583	7487391	
	b	7179810	8758131	8496914	7865748	5235606	5980421	7787251	
	c	7210382	8786597	8643022	7927184	5265705	6007312	7882931	
	mean	***7159129,67***	***8688159***	***8524974***	***7822402***	***5211605***	***5973772***	***7719191***	***7299890,429***
decrease between 1st and 2nd measurement [%]									33,87755118

		mice ID - ho						RLU
**measurement**	**replicates**	**1689**	**1696**	**1697**	**1722**	**1728**	**1738**	
1st (0 min.)	a	6959861	11571426	10123925	8600342	5554801	12496586	
	b	7134516	11712155	10307569	8730003	5632718	12869310	
	c	7181678	11796400	10357292	8759706	5655810	12922464	
	mean	***7092018***	***11693327***	***10262929***	***8696684***	***5614443***	***12762787***	***9353697,882***
2nd (+60 min.)	a	3516661	3246644	3722686	3532410	2207121	3385805	
	b	3563360	3281591	3753644	3599807	2205906	3478121	
	c	3585550	3303216	3776773	3628704	2199649	3497031	
	mean	***3555191***	***3277150***	***3751034***	***3586973***	***2204225***	***3453653***	***3304704,39***
decrease between 1st and 2nd measurement [%]								37,7647605

wt = wild type, het = heterozygous, ho - homozygous. RLU = relative luminescence unit.
