# Supplementary material for: Effects of Tcte1 knockout on energy chain transportation and spermatogenesis: implications for male infertility
Source: Hum Reprod Open. 2024 Apr 4;2024(2):hoae020. doi: 10.1093/hropen/hoae020 (PMC11035007; doi:10.1093/hropen/hoae020)

Supplementary Figure S1: Schematic models of exon 3 deleted in KO mice.

A Tcte1 protein exon 3 (deleted in KO) prediction of 5 top models (software: I-TASSER). C-score is the confidence score of the prediction. C-score ranges [0-1], where a higher score indicates a more reliable prediction; TM-score is a measure of global structural similarity between query and template protein., RMSD - root-mean-square deviation of the average distance between the atoms.
B The deletion sequence used for modeling, including exon 3 (yellow).

A

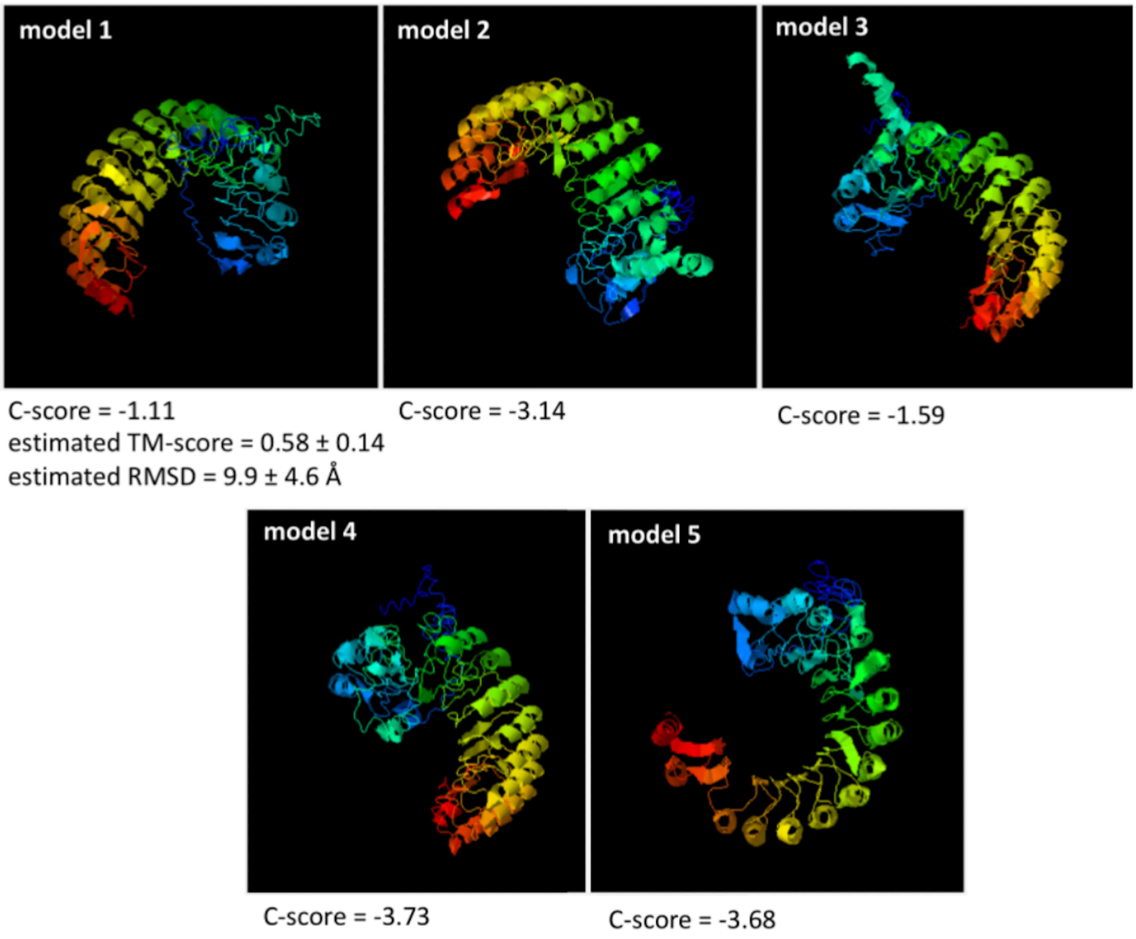

B

tgaagccgggcagtgggctcatgcctttaatcccagcacttgggaggcagaggcaggcggatttctgagttcgaggccagcctggtctacaaagt
gagttccaggacagccaaggctatacagagaaaccctgtctcgaaaaaccaaatatatatatatatatatatatatatatatatatatatatatat
atgacaccagataccgcccagtgcaattgccagttagttgcaggcaagagcaccctcaaagagtgtctctttagaagactatcctctcaaaataaa
gttcccgaggccacaaagggtctcccaagacagtccaggatgctgagttgcgatgttcccagcatggcggctgaaccctgttttccttcctcccttct
caaagacaaccctatcctgaagcagctgccgctagagcaccaaaagaaggtcttgagcaacctgcccccgagctgcctttgacggtgaccgcta
acttgattgacgatgagaattactggcaccgctgctgcatcaagcgctggtccgtgtgccacgtgtccaggcacggcggcagctggaagcgtatgt
tcttcgagagacacctggagaacctgctgaagctattcatcccggggaccacggaccccaacgtgatcctggacctgctgcccctctgcaggaact
acgtgcgccgcattcatgtggaccagttcctcccacctgtgcggatgccgaccccgctccagggagaggaacagtcggactctggcagtgagggc
gaggggagcgagccagagaaggaccactaccagctgcagacgctggtgggtggcctcaaacacctggaggagctggacctggtgtacggcgtc
aaagactgtggcatgaattttgagtggaacctctttctcttcacctaccgagactgctactctctggcagccaccatcaaagcttgccacaccctcaa
ggtactacccccacccccaccgcccccaactcacttgcctcctttctcattgtctttgggattgcttctccctgcctcttcctctccttctaccatcttct
ctcatttcctttgcattcatgagtagaggaagggattgtttagatagttagttgggtttttgttttgttttgttttgaaacttagttgaggcagggtctcat
accttaggcaggcctggaattcaatgtgtagctggaaacgaccttgaatttctgacctcctgcctccaccttacatgcacaaacatgaccaagtgct
gggatcacatgcgtatgacaccactacgccc

Supplementary Figure S2: Examples of hematoxylin and eosine-Y (HE) staining on testicular tissue.

Leica DM5500 light microscope (x40 dry objective, motorized stage), LASX software (with Navigator tool)
(Leica Microsystems GmbH, Wetzlar, Germany).
WT - wild type, HET - heterozygous males, HOM - homozygous males of Tcte1 knockout model.

WT

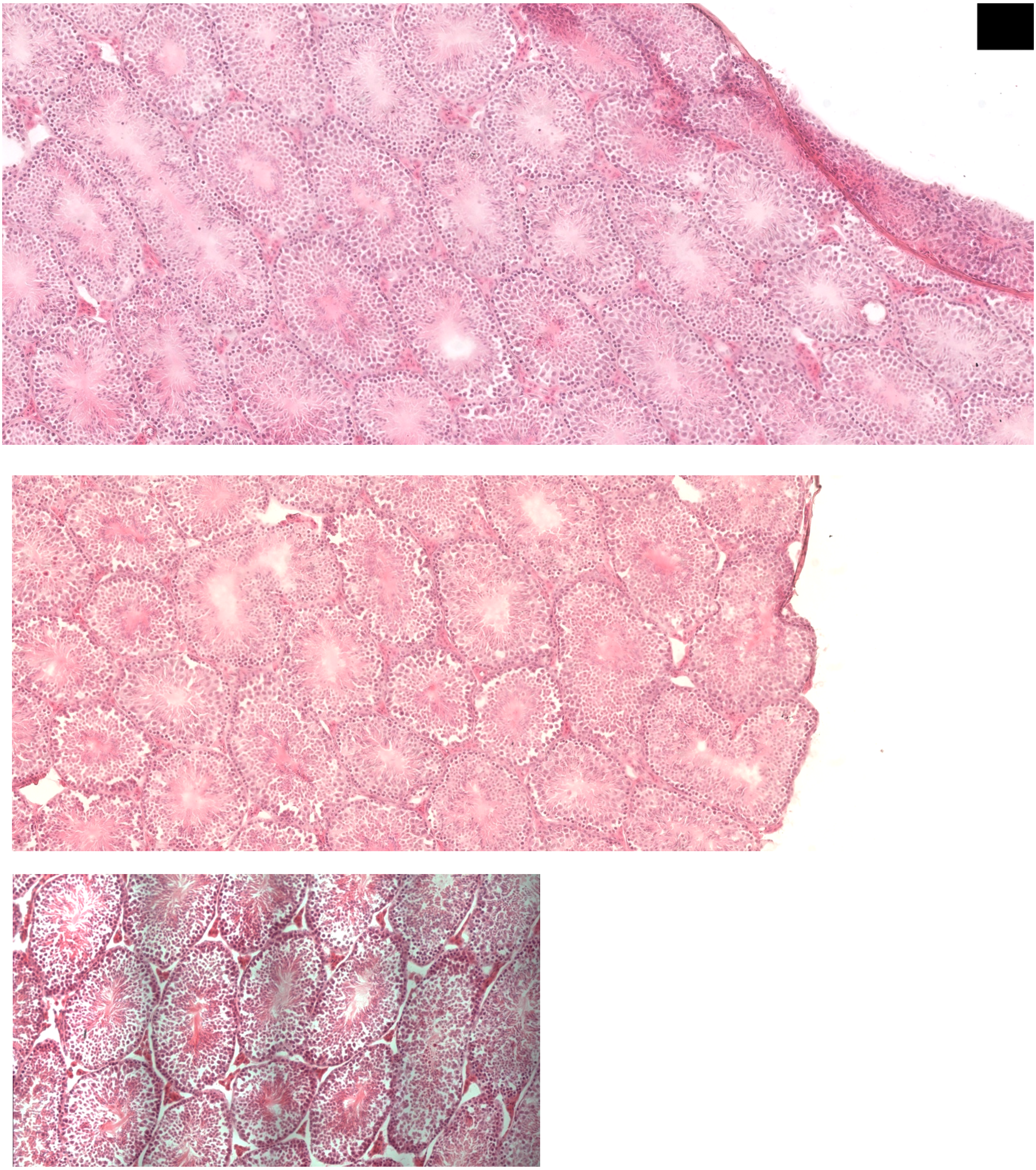

HET

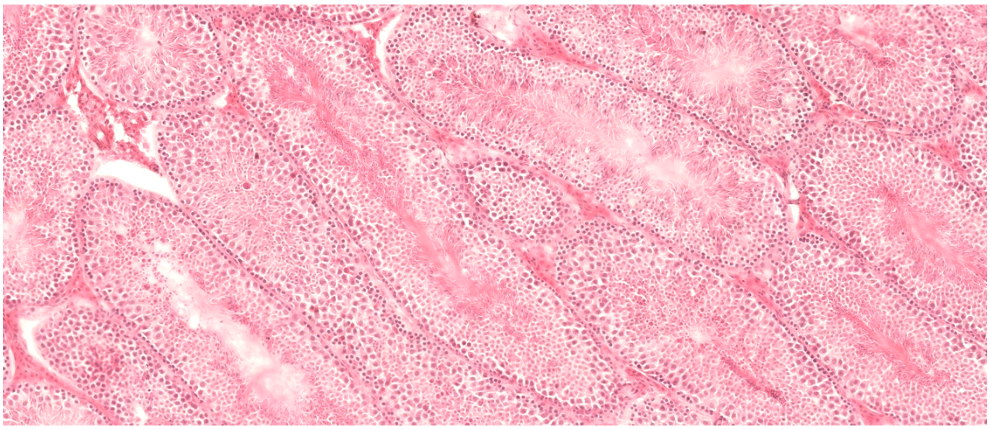

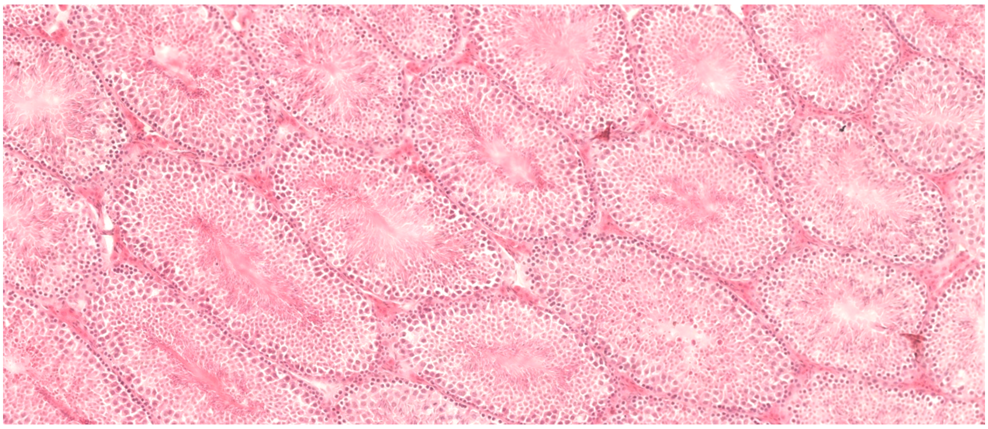

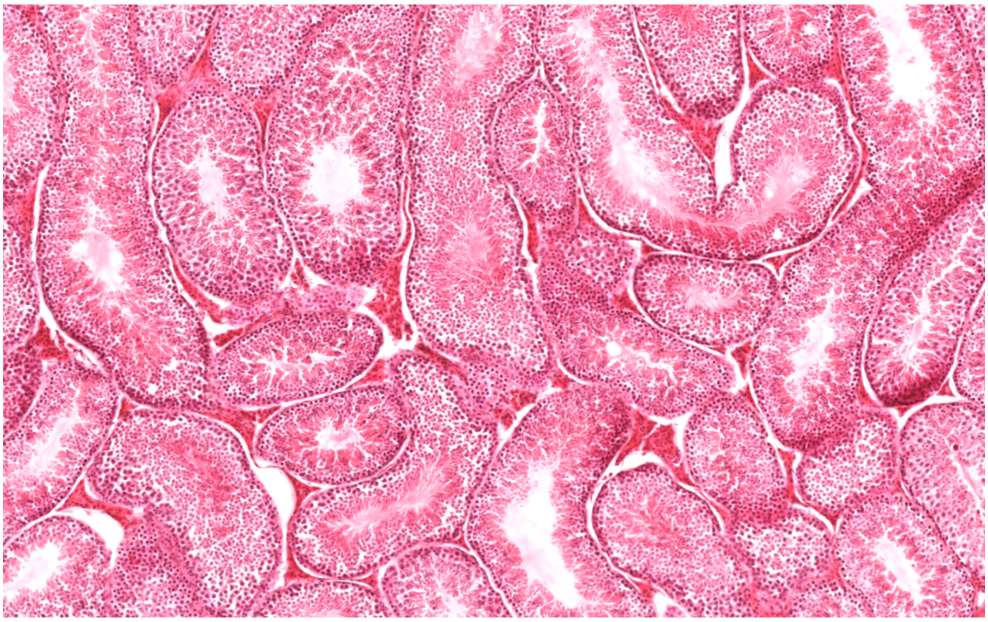

HOM

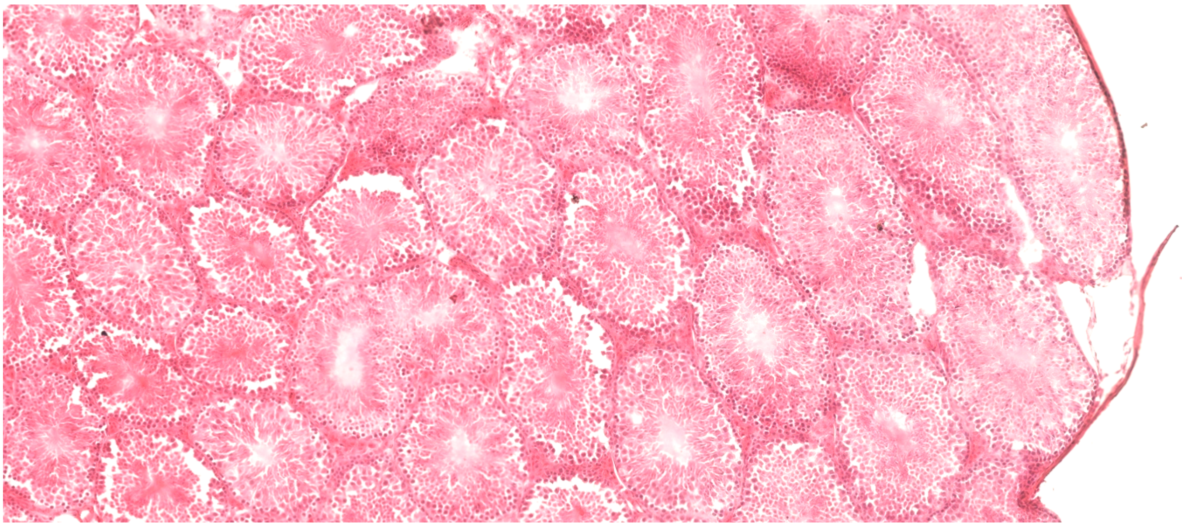

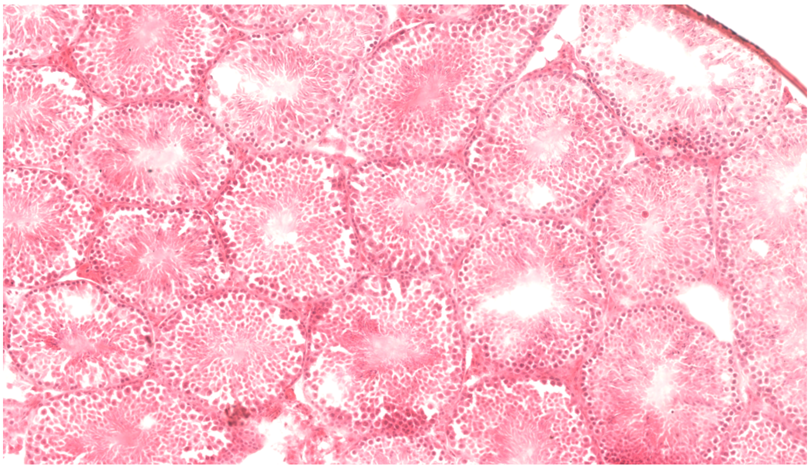

Supplementary Figure S3: STRING analyzis of potential interactions between proteins with observed major differences in gene expression level in mouse testis of the homozygous Tcte1 mice (Usp39, Fetub, Klk1b22).

According to genes revealed in RNAseq analysis (marked with arrows), two groups of interactions were formed for genes with highest expression changes (red arrows); (STRING database, 11th March 2021).

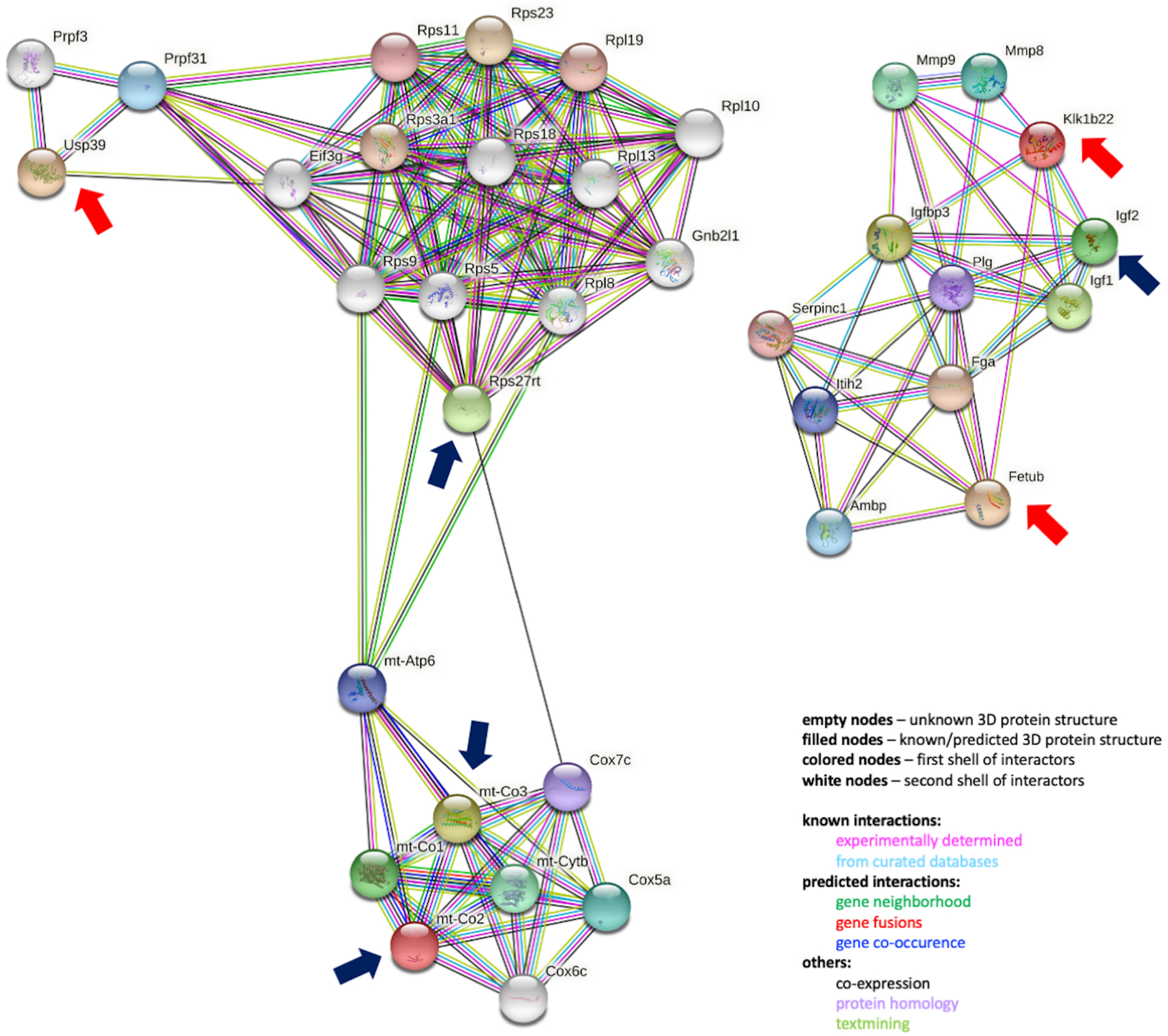

Supplementary Figure S4: Examples of potentially disease-causing rare heterozygous TCTE1 variants found in patients with disturbed spermatogenesis.

Results obtained from screening of n=248 participants.
A novel variant (Sanger sequencing);
B ultra rare variants (WES).

A novel variant

c.374T>G, p.IleArg

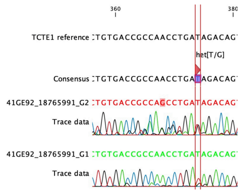

B ultra rare variants

c.862C>T, p.Arg288Ter

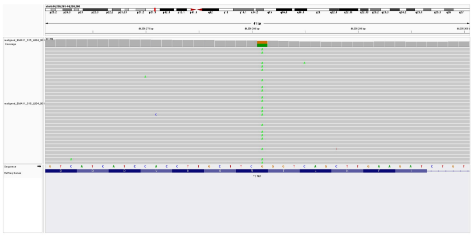

c.185G>A, p.Arg62His

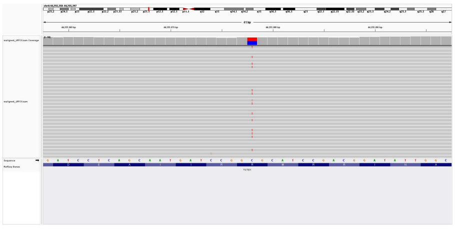

c.1048G>A, p.Gly350Ser

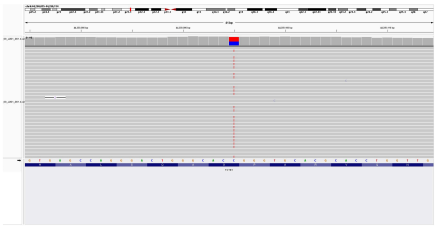

c.397C>T, p.Arg133Cys

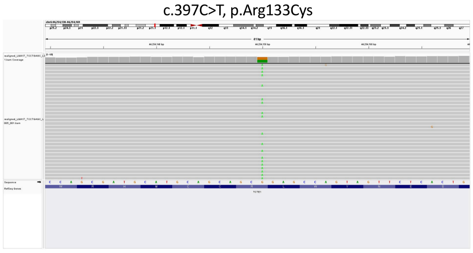

c.470A>C, p.Glu157Ala

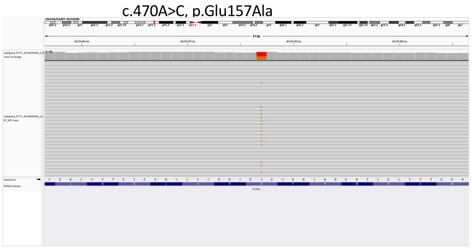

Supplementary Figure S5: The C-terminal region of TCTE1 was consistently modeled and fits well in published maps.

A Superposition of the cartoon representation of three models of human TCTE1 generated by Phyre2 [Kelley eta l., 2015] intensive (green) or fast mode (grey), I-TASSER [Roy et al., 2010; Yang et al., 2015; Yang and Zhang, 2015] (yellow), and AlphaFold (cyan). N- and C-termini are labeled.

B. Manual docking of the model of full-length TCTE1 generated by AlphaFold in maps calculated from a cryo-electron tomography experiment performed on C. reinhardtii using nanogold to locate the C-terminus of DRC5 (EMD-20821) [Gui et al., 2019].

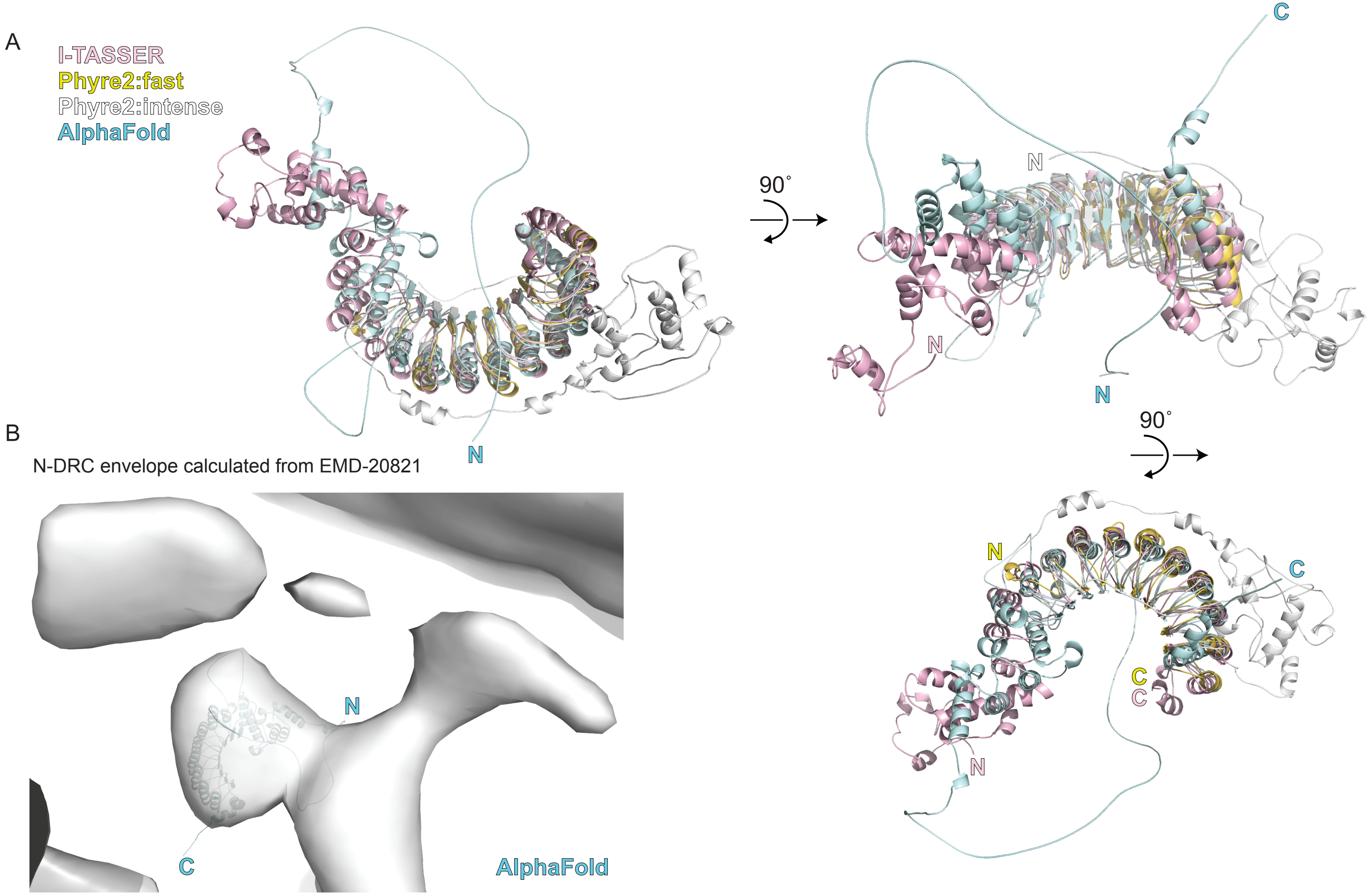

Supplement: hoae020_Supplementary_Data [file hoae020_supplementary_data.zip › Supplementary Figures S1-S5.pdf]
