# Supplementary material for: Effects of Tcte1 knockout on energy chain transportation and spermatogenesis: implications for male infertility
Source: Hum Reprod Open. 2024 Apr 4;2024(2):hoae020. doi: 10.1093/hropen/hoae020 (PMC11035007; doi:10.1093/hropen/hoae020)
Supplement: hoae020_Supplementary_Data [file hoae020_supplementary_data.zip › Titles and legends for Supplementary Videos S1 S2 and S3.docx]

**Titles and legends for Supplementary Videos S1, S2 and S3**

**Supplementary Video S1: Example of circular motility type of spermatozoa of *Tcte1^-/-^* knockout mice.**

**Supplementary Video S2: Example of circular motility type of spermatozoa of *Tcte1^+/-^* knockout mice.**

**Supplementary Video S3: Example of sperm cell motility in *Tcte^+/+^* knockout mice.**

All videos have been recorded using iPhone 11 iOS system, followed by Leica DM5500 microscope and microscope adapter for iPhone (iDu Optics LabCam).
