# Supplementary material for: Effects of Tcte1 knockout on energy chain transportation and spermatogenesis: implications for male infertility
Source: Hum Reprod Open. 2024 Apr 4;2024(2):hoae020. doi: 10.1093/hropen/hoae020 (PMC11035007; doi:10.1093/hropen/hoae020)

Supplementary Data File S1: Vectors used for knockout creation of Tcte1 gene.

Vector Summary

**Vector ID**	VB161011-1047mab
**Vector Name (official)**	pRP[CRISPR]-hCas9-U6>20nt_TGAGCCCACTGCCCGGCTTC
**Date Created (Pacific Time)**	2016-10-10
**Size**	8508 bp
**Vector Type**	Regular plasmid CRISPR vector (single gRNA)
**Inserted gRNA**	20nt_TGAGCCCACTGCCCGGCTTC
**Inserted Nuclease**	hCas9
**Target Sequence**	TGAGCCCACTGCCCGGCTTC
**Copy Number**	High
**Bacterial Resistance**	Ampicillin
**Cloning Host**	Stbl3

User Annotation of Vector

**Vector alias (from user)**	*None*
**Comment (from user)**	*None*

Vector Map

User-inserted region
Eukaryotic region
Bacterial region

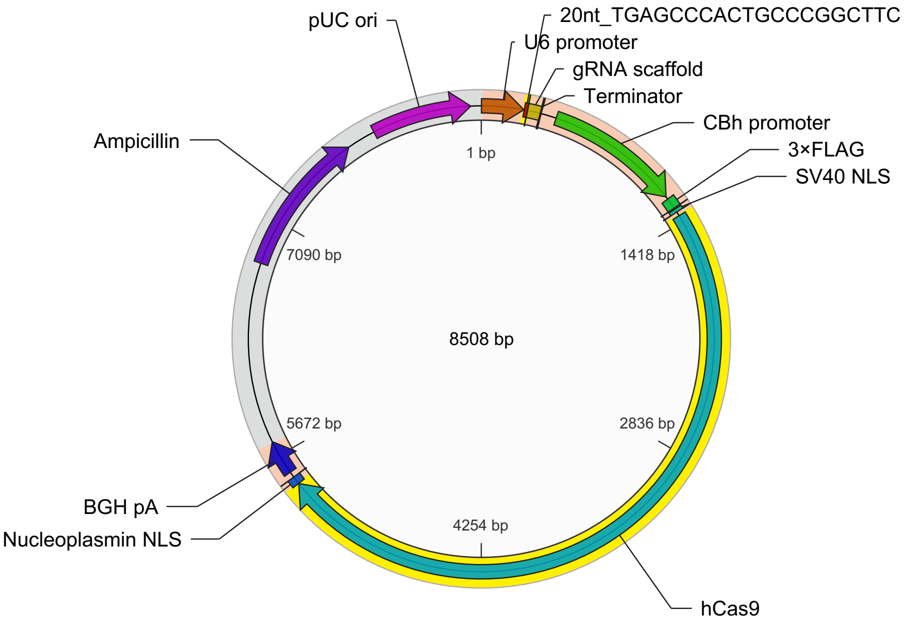

Vector Components

Component Name	Nucleotide Position	Full Name	Description
U6 promoter	1-249	Human U6 promoter	Allows high-level expression of gRNA.
20nt_TGAGCCCACTG CCCGGCTTC	251-270	20nt_TGAGCCCACTGCCCGGCTTC	Component entered by user
gRNA scaffold	271-346	Chimeric gRNA scaffold	Helps hCas9 bind to target DNA.
Terminator	347-352	U6 terminator	Allows transcription termination of gRNA.
CBh promoter	443-1240	Chicken betaActin hybrid promoter	Drives expression of hCas9.
3×FLAG	1256-1321	3 tandem flag epitopes	Allows to detect recombinant fusion proteins.
SV40 NLS	1328-1348	SV40 nuclear localization signal	Allows transportation of protein into the nucleus.
hCas9	1373-5473	Human codon-optimized Cas9	An RNA-guided DNA endonuclease enzyme associated with the CRISPR adaptive immunity system from Streptococcus pyogenes.
Nucleoplasmin NLS	5474-5521	Nucleoplasmin nuclear localization signal	Allows transportation of protein into the nucleus.
BGH pA	5555-5762	Bovine growth hormone polyadenylation	Allows transcription termination and polyadenylation of mRNA.
Ampicillin	6828-7688	Ampicillin resistance gene	Allows selection of the plasmid in E.coli.
pUC ori	7859-8447	pUC origin of replication	Permits high-copy replication and maintenance in E.coli.

Note: (c) denotes complementary strand.

User Annotation of Vector Components

Component Name	Comment by User
20nt_TGAGCCCACTGCCCGGCT TC	*None*
hCas9	*None*

Vector Sequence

```
   1 GAGGGCCTAT TTCCCATGAT TCCTTCATAT TTGCATATAC GATACAAGGC TGTTAGAGAG
  61 ATAATTGGAA TTAATTTGAC TGTAAACACA AAGATATTAG TACAAAATAC GTGACGTAGA
 121 AAGTAATAAT TTCTTGGGTA GTTTGCAGTT TTAAAATTAT GTTTTAAAAT GGACTATCAT
 181 ATGCTTACCG TAACTTGAAA GTATTTCGAT TTCTTGGCTT TATATATCTT GTGGAAAGGA
 241 CGAAACACCG TGAGCCCACT GCCCGGCTTC GTTTTAGAGC TAGAAATAGC AAGTTAAAAT
 301 AAGGCTAGTC CGTTATCAAC TTGAAAAAGT GGCACCGAGT CGGTGCTTTT TTGTTTTAGA
 361 GCTAGAAATA GCAAGTTAAA ATAAGGCTAG TCCGTTTTTA GCGCGTGCGC CAATTCTGCA
 421 GACAAATGGC TCTAGAGGTA CCCGTTACAT AACTTACGGT AAATGGCCCG CCTGGCTGAC
 481 CGCCCAACGA CCCCCGCCCA TTGACGTCAA TAGTAACGCC AATAGGGACT TTCCATTGAC
 541 GTCAATGGGT GGAGTATTTA CGGTAAACTG CCCACTTGGC AGTACATCAA GTGTATCATA
 601 TGCCAAGTAC GCCCCCTATT GACGTCAATG ACGGTAAATG GCCCGCCTGG CATTGTGCCC
 661 AGTACATGAC CTTATGGGAC TTTCCTACTT GGCAGTACAT CTACGTATTA GTCATCGCTA
 721 TTACCATGGT CGAGGTGAGC CCCACGTTCT GCTTCACTCT CCCCATCTCC CCCCCCTCCC
 781 CACCCCCAAT TTTGTATTTA TTTATTTTTT AATTATTTTG TGCAGCGATG GGGGCGGGGG
 841 GGGGGGGGGG GCGCGCGCCA GGCGGGGCGG GGCGGGGCGA GGGGCGGGGC GGGGCGAGGC
 901 GGAGAGGTGC GGCGGCAGCC AATCAGAGCG GCGCGCTCCG AAAGTTTCCT TTTATGGCGA
 961 GGCGGCGGCG GCGGCGGCCC TATAAAAAGC GAAGCGCGCG GCGGGCGGGA GTCGCTGCGC
1021 GCTGCCTTCG CCCCGTGCCC CGCTCCGCCG CCGCCTCGCG CCGCCCGCCC CGGCTCTGAC
1081 TGACCGCGTT ACTCCCACAG GTGAGCGGGC GGGACGGCCC TTCTCCTCCG GGCTGTAATT
1141 AGCTGAGCAA GAGGTAAGGG TTTAAGGGAT GGTTGGTTGG TGGGGTATTA ATGTTTAATT
1201 ACCTGGAGCA CCTGCCTGAA ATCACTTTTT TTCAGGTTGG ACCGGTGCCA CCATGGACTA
1261 TAAGGACCAC GACGGAGACT ACAAGGATCA TGATATTGAT TACAAAGACG ATGACGATAA
1321 GATGGCCCCA AAGAAGAAGC GGAAGGTCGG TATCCACGGA GTCCCAGCAG CCGACAAGAA
1381 GTACAGCATC GGCCTGGACA TCGGCACCAA CTCTGTGGGC TGGGCCGTGA TCACCGACGA
1441 GTACAAGGTG CCCAGCAAGA AATTCAAGGT GCTGGGCAAC ACCGACCGGC ACAGCATCAA
1501 GAAGAACCTG ATCGGAGCCC TGCTGTTCGA CAGCGGCGAA ACAGCCGAGG CCACCCGGCT
1561 GAAGAGAACC GCCAGAAGAA GATACACCAG ACGGAAGAAC CGGATCTGCT ATCTGCAAGA
1621 GATCTTCAGC AACGAGATGG CCAAGGTGGA CGACAGCTTC TTCCACAGAC TGGAAGAGTC
1681 CTTCCTGGTG GAAGAGGATA AGAAGCACGA GCGGCACCCC ATCTTCGGCA ACATCGTGGA
1741 CGAGGTGGCC TACCACGAGA AGTACCCCAC CATCTACCAC CTGAGAAAGA AACTGGTGGA
1801 CAGCACCGAC AAGGCCGACC TGCGGCTGAT CTATCTGGCC CTGGCCCACA TGATCAAGTT
1861 CCGGGGCCAC TTCCTGATCG AGGGCGACCT GAACCCCGAC AACAGCGACG TGGACAAGCT
1921 GTTCATCCAG CTGGTGCAGA CCTACAACCA GCTGTTCGAG GAAAACCCCA TCAACGCCAG
1981 CGGCGTGGAC GCCAAGGCCA TCCTGTCTGC CAGACTGAGC AAGAGCAGAC GGCTGGAAAA
2041 TCTGATCGCC CAGCTGCCCG GCGAGAAGAA GAATGGCCTG TTCGGAAACC TGATTGCCCT
2101 GAGCCTGGGC CTGACCCCCA ACTTCAAGAG CAACTTCGAC CTGGCCGAGG ATGCCAAACT
2161 GCAGCTGAGC AAGGACACCT ACGACGACGA CCTGGACAAC CTGCTGGCCC AGATCGGCGA
2221 CCAGTACGCC GACCTGTTTC TGGCCGCCAA GAACCTGTCC GACGCCATCC TGCTGAGCGA
2281 CATCCTGAGA GTGAACACCG AGATCACCAA GGCCCCCCTG AGCGCCTCTA TGATCAAGAG
2341 ATACGACGAG CACCACCAGG ACCTGACCCT GCTGAAAGCT CTCGTGCGGC AGCAGCTGCC
2401 TGAGAAGTAC AAAGAGATTT TCTTCGACCA GAGCAAGAAC GGCTACGCCG GCTACATTGA
2461 CGGCGGAGCC AGCCAGGAAG AGTTCTACAA GTTCATCAAG CCCATCCTGG AAAAGATGGA
2521 CGGCACCGAG GAACTGCTCG TGAAGCTGAA CAGAGAGGAC CTGCTGCGGA AGCAGCGGAC
2581 CTTCGACAAC GGCAGCATCC CCCACCAGAT CCACCTGGGA GAGCTGCACG CCATTCTGCG
2641 GCGGCAGGAA GATTTTTACC CATTCCTGAA GGACAACCGG GAAAAGATCG AGAAGATCCT
2701 GACCTTCCGC ATCCCCTACT ACGTGGGCCC TCTGGCCAGG GGAAACAGCA GATTCGCCTG
2761 GATGACCAGA AAGAGCGAGG AAACCATCAC CCCCTGGAAC TTCGAGGAAG TGGTGGACAA
2821 GGGCGCTTCC GCCCAGAGCT TCATCGAGCG GATGACCAAC TTCGATAAGA ACCTGCCCAA
2881 CGAGAAGGTG CTGCCCAAGC ACAGCCTGCT GTACGAGTAC TTCACCGTGT ATAACGAGCT
2941 GACCAAAGTG AAATACGTGA CCGAGGGAAT GAGAAAGCCC GCCTTCCTGA GCGGCGAGCA
```

3001 GAAAAAGGCC ATCGTGGACC TGCTGTTCAA GACCAACCGG AAAGTGACCG TGAAGCAGCT
3061 GAAAGAGGAC TACTTCAAGA AAATCGAGTG CTTCGACTCC GTGGAAATCT CCGGCGTGGA
3121 AGATCGGTTC AACGCCTCCC TGGGCACATA CCACGATCTG CTGAAAATTA TCAAGGACAA
3181 GGACTTCCTG GACAATGAGG AAAACGAGGA CATTCTGGAA GATATCGTGC TGACCCTGAC
3241 ACTGTTTGAG GACAGAGAGA TGATCGAGGA ACGGCTGAAA ACCTATGCCC ACCTGTTCGA
3301 CGACAAAGTG ATGAAGCAGC TGAAGCGGCG GAGATACACC GGCTGGGGCA GGCTGAGCCG
3361 GAAGCTGATC AACGGCATCC GGGACAAGCA GTCCGGCAAG ACAATCCTGG ATTTCCTGAA
3421 GTCCGACGGC TTCGCCAACA GAAACTTCAT GCAGCTGATC CACGACGACA GCCTGACCTT
3481 TAAAGAGGAC ATCCAGAAAG CCCAGGTGTC CGGCCAGGGC GATAGCCTGC ACGAGCACAT
3541 TGCCAATCTG GCCGGCAGCC CCGCCATTAA GAAGGGCATC CTGCAGACAG TGAAGGTGGT
3601 GGACGAGCTC GTGAAAGTGA TGGGCCGGCA CAAGCCCGAG AACATCGTGA TCGAAATGGC
3661 CAGAGAGAAC CAGACCACCC AGAAGGGACA GAAGAACAGC CGCGAGAGAA TGAAGCGGAT
3721 CGAAGAGGGC ATCAAAGAGC TGGGCAGCCA GATCCTGAAA GAACACCCCG TGGAAAACAC
3781 CCAGCTGCAG AACGAGAAGC TGTACCTGTA CTACCTGCAG AATGGGCGGG ATATGTACGT
3841 GGACCAGGAA CTGGACATCA ACCGGCTGTC CGACTACGAT GTGGACCATA TCGTGCCTCA
3901 GAGCTTTCTG AAGGACGACT CCATCGACAA CAAGGTGCTG ACCAGAAGCG ACAAGAACCG
3961 GGGCAAGAGC GACAACGTGC CCTCCGAAGA GGTCGTGAAG AAGATGAAGA ACTACTGGCG
4021 GCAGCTGCTG AACGCCAAGC TGATTACCCA GAGAAAGTTC GACAATCTGA CCAAGGCCGA
4081 GAGAGGCGGC CTGAGCGAAC TGGATAAGGC CGGCTTCATC AAGAGACAGC TGGTGGAAAC
4141 CCGGCAGATC ACAAAGCACG TGGCACAGAT CCTGGACTCC CGGATGAACA CTAAGTACGA
4201 CGAGAATGAC AAGCTGATCC GGGAAGTGAA AGTGATCACC CTGAAGTCCA AGCTGGTGTC
4261 CGATTTCCGG AAGGATTTCC AGTTTTACAA AGTGCGCGAG ATCAACAACT ACCACCACGC
4321 CCACGACGCC TACCTGAACG CCGTCGTGGG AACCGCCCTG ATCAAAAAGT ACCCTAAGCT
4381 GGAAAGCGAG TTCGTGTACG GCGACTACAA GGTGTACGAC GTGCGGAAGA TGATCGCCAA
4441 GAGCGAGCAG GAAATCGGCA AGGCTACCGC CAAGTACTTC TTCTACAGCA ACATCATGAA
4501 CTTTTTCAAG ACCGAGATTA CCCTGGCCAA CGGCGAGATC CGGAAGCGGC CTCTGATCGA
4561 GACAAACGGC GAAACCGGGG AGATCGTGTG GGATAAGGGC CGGGATTTTG CCACCGTGCG
4621 GAAAGTGCTG AGCATGCCCC AAGTGAATAT CGTGAAAAAG ACCGAGGTGC AGACAGGCGG
4681 CTTCAGCAAA GAGTCTATCC TGCCCAAGAG GAACAGCGAT AAGCTGATCG CCAGAAAGAA
4741 GGACTGGGAC CCTAAGAAGT ACGGCGGCTT CGACAGCCCC ACCGTGGCCT ATTCTGTGCT
4801 GGTGGTGGCC AAAGTGGAAA AGGGCAAGTC CAAGAAACTG AAGAGTGTGA AAGAGCTGCT
4861 GGGGATCACC ATCATGGAAA GAAGCAGCTT CGAGAAGAAT CCCATCGACT TTCTGGAAGC
4921 CAAGGGCTAC AAAGAAGTGA AAAAGGACCT GATCATCAAG CTGCCTAAGT ACTCCCTGTT
4981 CGAGCTGGAA AACGGCCGGA AGAGAATGCT GGCCTCTGCC GGCGAACTGC AGAAGGGAAA
5041 CGAACTGGCC CTGCCCTCCA AATATGTGAA CTTCCTGTAC CTGGCCAGCC ACTATGAGAA
5101 GCTGAAGGGC TCCCCCGAGG ATAATGAGCA GAAACAGCTG TTTGTGGAAC AGCACAAGCA
5161 CTACCTGGAC GAGATCATCG AGCAGATCAG CGAGTTCTCC AAGAGAGTGA TCCTGGCCGA
5221 CGCTAATCTG GACAAAGTGC TGTCCGCCTA CAACAAGCAC CGGGATAAGC CCATCAGAGA
5281 GCAGGCCGAG AATATCATCC ACCTGTTTAC CCTGACCAAT CTGGGAGCCC CTGCCGCCTT
5341 CAAGTACTTT GACACCACCA TCGACCGGAA GAGGTACACC AGCACCAAAG AGGTGCTGGA
5401 CGCCACCCTG ATCCACCAGA GCATCACCGG CCTGTACGAG ACACGGATCG ACCTGTCTCA
5461 GCTGGGAGGC GAC**AAAAGGC CGGCGGCCAC GAAAAAGGCC GGCCAGGCAA AAAAGAAAAA**
5521 **G**TAAGAATTC CTAGAGCTCG CTGATCAGCC TCGACTGTGC CTTCTAGTTG CCAGCCATCT
5581 GTTGTTTGCC CCTCCCCCGT GCCTTCCTTG ACCCTGGAAG GTGCCACTCC CACTGTCCTT
5641 TCCTAATAAA ATGAGGAAAT TGCATCGCAT TGTCTGAGTA GGTGTCATTC TATTCTGGGG
5701 GGTGGGGTGG GGCAGGACAG CAAGGGGGAG GATTGGGAAG AGAATAGCAG GCATGCTGGG
5761 GAGCGGCCGC AGGAACCCCT AGTGATGGAG TTGGCCACTC CCTCTCTGCG CGCTCGCTCG
5821 CTCACTGAGG CCGGGCGACC AAAGGTCGCC CGACGCCCGG GCTTTGCCCG GGCGGCCTCA
5881 GTGAGCGAGC GAGCGCGCAG CTGCCTGCAG GGGCGCCTGA TGCGGTATTT TCTCCTTACG
5941 CATCTGTGCG GTATTTCACA CCGCATACGT CAAAGCAACC ATAGTACGCG CCCTGTAGCG
6001 GCGCATTAAG CGCGGCGGGT GTGGTGGTTA CGCGCAGCGT GACCGCTACA CTTGCCAGCG
6061 CCTTAGCGCC CGCTCCTTTC GCTTTCTTCC CTTCCTTTCT CGCCACGTTC GCCGGCTTTC

```
6121  CCCGTCAAGC TCTAAATCGG GGGCTCCCTT TAGGGTTCCG ATTTAGTGCT TTACGGCACC
6181  TCGACCCCAA AAAACTTGAT TTGGGTGATG GTTCACGTAG TGGGCCATCG CCCTGATAGA
6241  CGGTTTTTCG CCCTTTGACG TTGGAGTCCA CGTTCTTTAA TAGTGGACTC TTGTTCCAAA
6301  CTGGAACAAC ACTCAACTCT ATCTCGGGCT ATTCTTTTGA TTTATAAGGG ATTTTGCCGA
6361  TTTCGGTCTA TTGGTTAAAA AATGAGCTGA TTTAACAAAA ATTTAACGCG AATTTTAACA
6421  AAATATTAAC GTTTACAATT TTATGGTGCA CTCTCAGTAC AATCTGCTCT GATGCCGCAT
6481  AGTTAAGCCA GCCCCGACAC CCGCCAACAC CCGCTGACGC GCCCTGACGG GCTTGTCTGC
6541  TCCCGGCATC CGCTTACAGA CAAGCTGTGA CCGTCTCCGG GAGCTGCATG TGTCAGAGGT
6601  TTTCACCGTC ATCACCGAAA CGCGCGAGAC GAAAGGGCCT CGTGATACGC CTATTTTTAT
6661  AGGTTAATGT CATGATAATA ATGGTTTCTT AGACGTCAGG TGGCACTTTT CGGGGAAATG
6721  TGCGCGGAAC CCCTATTTGT TTATTTTTCT AAATACATTC AAATATGTAT CCGCTCATGA
6781  GACAATAACC CTGATAAATG CTTCAATAAT ATTGAAAAAG GAAGAGTATG AGTATTCAAC
6841  ATTTCCGTGT CGCCCTTATT CCCTTTTTTG CGGCATTTTG CCTTCCTGTT TTTGCTCACC
6901  CAGAAACGCT GGTGAAAGTA AAAGATGCTG AAGATCAGTT GGGTGCACGA GTGGGTTACA
6961  TCGAACTGGA TCTCAACAGC GGTAAGATCC TTGAGAGTTT TCGCCCCGAA GAACGTTTTC
7021  CAATGATGAG CACTTTTAAA GTTCTGCTAT GTGGCGCGGT ATTATCCCGT ATTGACGCCG
7081  GGCAAGAGCA ACTCGGTCGC CGCATACACT ATTCTCAGAA TGACTTGGTT GAGTACTCAC
7141  CAGTCACAGA AAAGCATCTT ACGGATGGCA TGACAGTAAG AGAATTATGC AGTGCTGCCA
7201  TAACCATGAG TGATAACACT GCGGCCAACT TACTTCTGAC AACGATCGGA GGACCGAAGG
7261  AGCTAACCGC TTTTTTGCAC AACATGGGGG ATCATGTAAC TCGCCTTGAT CGTTGGGAAC
7321  CGGAGCTGAA TGAAGCCATA CCAAACGACG AGCGTGACAC CACGATGCCT GTAGCAATGG
7381  CAACAACGTT GCGCAAACTA TTAACTGGCG AACTACTTAC TCTAGCTTCC CGGCAACAAT
7441  TAATAGACTG GATGGAGGCG GATAAAGTTG CAGGACCACT TCTGCGCTCG GCCCTTCCGG
7501  CTGGCTGGTT TATTGCTGAT AAATCTGGAG CCGGTGAGCG TGGAAGCCGC GGTATCATTG
7561  CAGCACTGGG GCCAGATGGT AAGCCCTCCC GTATCGTAGT TATCTACACG ACGGGGAGTC
7621  AGGCAACTAT GGATGAACGA AATAGACAGA TCGCTGAGAT AGGTGCCTCA CTGATTAAGC
7681  ATTGGTAACT GTCAGACCAA GTTTACTCAT ATATACTTTA GATTGATTTA AAACTTCATT
7741  TTTAATTTAA AAGGATCTAG GTGAAGATCC TTTTTGATAA TCTCATGACC AAAATCCCTT
7801  AACGTGAGTT TTCGTTCCAC TGAGCGTCAG ACCCCGTAGA AAAGATCAAA GGATCTTCTT
7861  GAGATCCTTT TTTTCTGCGC GTAATCTGCT GCTTGCAAAC AAAAAAACCA CCGCTACCAG
7921  CGGTGGTTTG TTTGCCGGAT CAAGAGCTAC CAACTCTTTT TCCGAAGGTA ACTGGCTTCA
7981  GCAGAGCGCA GATACCAAAT ACTGTTCTTC TAGTGTAGCC GTAGTTAGGC CACCACTTCA
8041  AGAACTCTGT AGCACCGCCT ACATACCTCG CTCTGCTAAT CCTGTTACCA GTGGCTGCTG
8101  CCAGTGGCGA TAAGTCGTGT CTTACCGGGT TGGACTCAAG ACGATAGTTA CCGGATAAGG
8161  CGCAGCGGTC GGGCTGAACG GGGGGTTCGT GCACACAGCC CAGCTTGGAG CGAACGACCT
8221  ACACCGAACT GAGATACCTA CAGCGTGAGC TATGAGAAAG CGCCACGCTT CCCGAAGGGA
8281  GAAAGGCGGA CAGGTATCCG GTAAGCGGCA GGGTCGGAAC AGGAGAGCGC ACGAGGGAGC
8341  TTCCAGGGGG AAACGCCTGG TATCTTTATA GTCCTGTCGG GTTTCGCCAC CTCTGACTTG
8401  AGCGTCGATT TTTGTGATGC TCGTCAGGGG GGCGGAGCCT ATGGAAAAAC GCCAGCAACG
8461  CGGCCTTTTT ACGGTTCCTG GCCTTTTGCT GGCCTTTTGC TCACATGT
```

Qualification by Sequencing

Primer Name	Primer Sequence	Strand
**gRNA-F1**	CGGAGCCTATGGAAAAACGC	Forward
**hCas9-R1**	CAGATCCGGTTCTTCCGTCT	Reverse

Qualification by Restriction Enzyme Digestion

Cutters	Locations	Fragments(bp)
**ScaI**	2919, 4476, 4971, 5346, 7135	1557, 495, 375, 1789, 4292

Vector Summary

**Vector ID**	VB161011-1048psn
**Vector Name (official)**	pRP[CRISPR]-hCas9-U6>20nt_ATGACTTAGACTGGGCGTAG
**Date Created (Pacific Time)**	2016-10-10
**Size**	8508 bp
**Vector Type**	Regular plasmid CRISPR vector (single gRNA)
**Inserted gRNA**	20nt_ATGACTTAGACTGGGCGTAG
**Inserted Nuclease**	hCas9
**Target Sequence**	ATGACTTAGACTGGGCGTAG
**Copy Number**	High
**Bacterial Resistance**	Ampicillin
**Cloning Host**	Stbl3

User Annotation of Vector

**Vector alias (from user)**	*None*
**Comment (from user)**	*None*

Vector Map

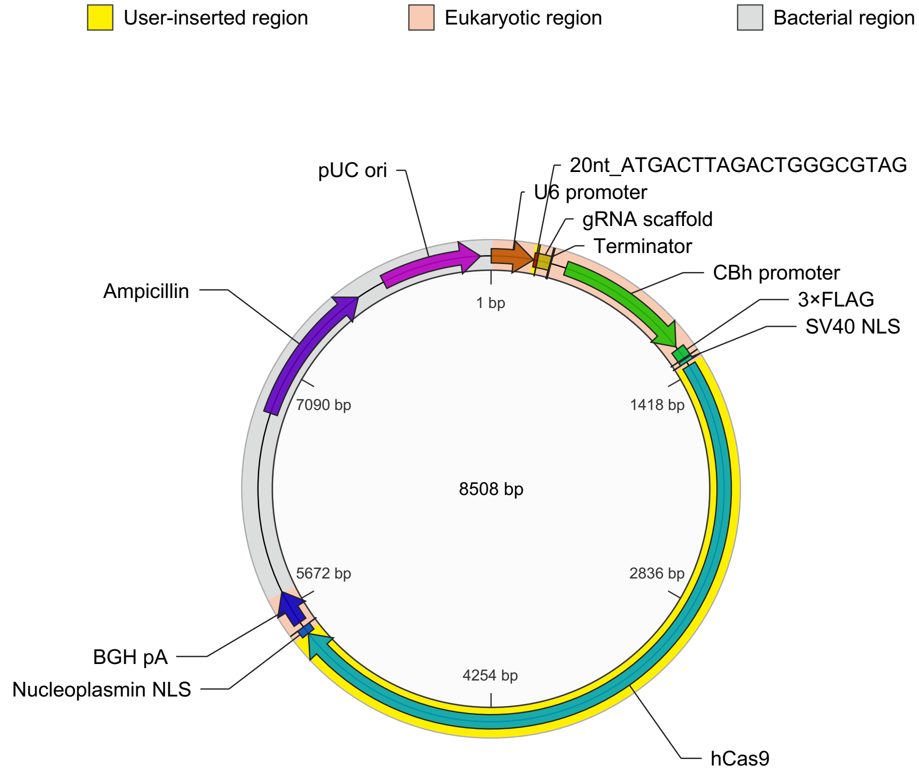
User-inserted region
Eukaryotic region
Bacterial region
pUC ori
20nt_ATGACTTAGACTGGGCGTAG
U6 promoter
gRNA scaffold
Terminator
CBh promoter
3×FLAG
SV40 NLS
Ampicillin
1 bp
7090 bp
1418 bp
8508 bp
5672 bp
2836 bp
4254 bp
BGH pA
Nucleoplasmin NLS
hCas9

Vector Components

Component Name	Nucleotide Position	Full Name	Description
U6 promoter	**1-249**	Human U6 promoter	Allows high-level expression of gRNA.
20nt_ATGACTTAGACT GGGCGTAG	251-270	20nt_ATGACTTAGACTGGGCGTAG	Component entered by user
gRNA scaffold	**271-346**	Chimeric gRNA scaffold	Helps hCas9 bind to target DNA.
Terminator	347-352	U6 terminator	Allows transcription termination of gRNA.
CBh promoter	**443-1240**	Chicken betaActin hybrid promoter	Drives expression of hCas9.
3×FLAG	1256-1321	3 tandem flag epitopes	Allows to detect recombinant fusion proteins.
SV40 NLS	**1328-1348**	SV40 nuclear localization signal	Allows transportation of protein into the nucleus.
hCas9	1373-5473	Human codon-optimized Cas9	An RNA-guided DNA endonuclease enzyme associated with the CRISPR adaptive immunity system from Streptococcus pyogenes.
Nucleoplasmin NLS	**5474-5521**	Nucleoplasmin nuclear localization signal	Allows transportation of protein into the nucleus.
BGH pA	5555-5762	Bovine growth hormone polyadenylation	Allows transcription termination and polyadenylation of mRNA.
Ampicillin	**6828-7688**	Ampicillin resistance gene	Allows selection of the plasmid in E.coli.
pUC ori	7859-8447	pUC origin of replication	Permits high-copy replication and maintenance in E.coli.

Note: (c) denotes complementary strand.

User Annotation of Vector Components

Component Name	Comment by User
20nt_ATGACTTAGACTGGGCGT AG	*None*
hCas9	*None*

Vector Sequence

1	GAGGGCCTAT	TTCCCATGAT	TCCTTCATAT	TTGCATATAC	GATACAAGGC	TGTTAGAGAG
61	ATAATTGGAA	TTAATTTGAC	TGTAAACACA	AAGATATTAG	TACAAAATAC	GTGACGTAGA
121	AAGTAATAAT	TTCTTGGGTA	GTTTGCAGTT	TTAAAATTAT	GTTTTAAAAT	GGACTATCAT
181	ATGCTTACCG	TAACTTGAAA	GTATTTCGAT	TTCTTGGCTT	TATATATCTT	GTGGAAAGGA
241	CGAAACACCG	ATGACTTAGA	CTGGGCGTAG	GTTTTAGAGC	TAGAAATAGC	AAGTTAAAAT
301	AAGGCTAGTC	CGTTATCAAC	TTGAAAAAGT	GGCACCGAGT	CGGTGCTTTT	TTGTTTTAGA
361	GCTAGAAATA	GCAAGTTAAA	ATAAGGCTAG	TCCGTTTTTA	GCGCGTGCGC	CAATTCTGCA
421	GACAAATGGC	TCTAGAGGTA	CCCGTTACAT	AACTTACGGT	AAATGGCCCG	CCTGGCTGAC
481	CGCCCAACGA	CCCCCGCCCA	TTGACGTCAA	TAGTAACGCC	AATAGGGACT	TTCCATTGAC
541	GTCAATGGGT	GGAGTATTTA	CGGTAAACTG	CCCACTTGGC	AGTACATCAA	GTGTATCATA
601	TGCCAAGTAC	GCCCCCTATT	GACGTCAATG	ACGGTAAATG	GCCCGCCTGG	CATTGTGCCC
661	AGTACATGAC	CTTATGGGAC	TTTCCTACTT	GGCAGTACAT	CTACGTATTA	GTCATCGCTA
721	TTACCATGGT	CGAGGTGAGC	CCCACGTTCT	GCTTCACTCT	CCCCATCTCC	CCCCCCTCCC
781	CACCCCCAAT	TTTGTATTTA	TTTATTTTTT	AATTATTTTG	TGCAGCGATG	GGGGCGGGGG
841	GGGGGGGGGG	GCGCGCGCCA	GGCGGGGCGG	GGCGGGGCGA	GGGGCGGGGC	GGGGCGAGGC
901	GGAGAGGTGC	GGCGGCAGCC	AATCAGAGCG	GCGCGCTCCG	AAAGTTTCCT	TTTATGGCGA
961	GGCGGCGGCG	GCGGCGGCCC	TATAAAAAGC	GAAGCGCGCG	GCGGGCGGGA	GTCGCTGCGC
1021	GCTGCCTTCG	CCCCGTGCCC	CGCTCCGCCG	CCGCCTCGCG	CCGCCCGCCC	CGGCTCTGAC
1081	TGACCGCGTT	ACTCCCACAG	GTGAGCGGGC	GGGACGGCCC	TTCTCCTCCG	GGCTGTAATT
1141	AGCTGAGCAA	GAGGTAAGGG	TTTAAGGGAT	GGTTGGTTGG	TGGGGTATTA	ATGTTTAATT
1201	ACCTGGAGCA	CCTGCCTGAA	ATCACTTTTT	TTCAGGTTGG	ACCGGTGCCA	CCATGGACTA
1261	TAAGGACCAC	GACGGAGACT	ACAAGGATCA	TGATATTGAT	TACAAAGACG	ATGACGATAA
1321	GATGGCCCCA	AAGAAGAAGC	GGAAGGTCGG	TATCCACGGA	GTCCCAGCAG	CCGACAAGAA
1381	GTACAGCATC	GGCCTGGACA	TCGGCACCAA	CTCTGTGGGC	TGGGCCGTGA	TCACCGACGA
1441	GTACAAGGTG	CCCAGCAAGA	AATTCAAGGT	GCTGGGCAAC	ACCGACCGGC	ACAGCATCAA
1501	GAAGAACCTG	ATCGGAGCCC	TGCTGTTCGA	CAGCGGCGAA	ACAGCCGAGG	CCACCCGGCT
1561	GAAGAGAACC	GCCAGAAGAA	GATACACCAG	ACGGAAGAAC	CGGATCTGCT	ATCTGCAAGA
1621	GATCTTCAGC	AACGAGATGG	CCAAGGTGGA	CGACAGCTTC	TTCCACAGAC	TGGAAGAGTC
1681	CTTCCTGGTG	GAAGAGGATA	AGAAGCACGA	GCGGCACCCC	ATCTTCGGCA	ACATCGTGGA
1741	CGAGGTGGCC	TACCACGAGA	AGTACCCCAC	CATCTACCAC	CTGAGAAAGA	AACTGGTGGA
1801	CAGCACCGAC	AAGGCCGACC	TGCGGCTGAT	CTATCTGGCC	CTGGCCCACA	TGATCAAGTT
1861	CCGGGGCCAC	TTCCTGATCG	AGGGCGACCT	GAACCCCGAC	AACAGCGACG	TGGACAAGCT
1921	GTTCATCCAG	CTGGTGCAGA	CCTACAACCA	GCTGTTCGAG	GAAAACCCCA	TCAACGCCAG
1981	CGGCGTGGAC	GCCAAGGCCA	TCCTGTCTGC	CAGACTGAGC	AAGAGCAGAC	GGCTGGAAAA
2041	TCTGATCGCC	CAGCTGCCCG	GCGAGAAGAA	GAATGGCCTG	TTCGGAAACC	TGATTGCCCT
2101	GAGCCTGGGC	CTGACCCCCA	ACTTCAAGAG	CAACTTCGAC	CTGGCCGAGG	ATGCCAAACT
2161	GCAGCTGAGC	AAGGACACCT	ACGACGACGA	CCTGGACAAC	CTGCTGGCCC	AGATCGGCGA
2221	CCAGTACGCC	GACCTGTTTC	TGGCCGCCAA	GAACCTGTCC	GACGCCATCC	TGCTGAGCGA
2281	CATCCTGAGA	GTGAACACCG	AGATCACCAA	GGCCCCCCTG	AGCGCCTCTA	TGATCAAGAG
2341	ATACGACGAG	CACCACCAGG	ACCTGACCCT	GCTGAAAGCT	CTCGTGCGGC	AGCAGCTGCC
2401	TGAGAAGTAC	AAAGAGATTT	TCTTCGACCA	GAGCAAGAAC	GGCTACGCCG	GCTACATTGA
2461	CGGCGGAGCC	AGCCAGGAAG	AGTTCTACAA	GTTCATCAAG	CCCATCCTGG	AAAAGATGGA
2521	CGGCACCGAG	GAACTGCTCG	TGAAGCTGAA	CAGAGAGGAC	CTGCTGCGGA	AGCAGCGGAC
2581	CTTCGACAAC	GGCAGCATCC	CCCACCAGAT	CCACCTGGGA	GAGCTGCACG	CCATTCTGCG
2641	GCGGCAGGAA	GATTTTTACC	CATTCCTGAA	GGACAACCGG	GAAAAGATCG	AGAAGATCCT
2701	GACCTTCCGC	ATCCCCTACT	ACGTGGGCCC	TCTGGCCAGG	GGAAACAGCA	GATTCGCCTG
2761	GATGACCAGA	AAGAGCGAGG	AAACCATCAC	CCCCTGGAAC	TTCGAGGAAG	TGGTGGACAA
2821	GGGCGCTTCC	GCCCAGAGCT	TCATCGAGCG	GATGACCAAC	TTCGATAAGA	ACCTGCCCAA
2881	CGAGAAGGTG	CTGCCCAAGC	ACAGCCTGCT	GTACGAGTAC	TTCACCGTGT	ATAACGAGCT
2941	GACCAAAGTG	AAATACGTGA	CCGAGGGAAT	GAGAAAGCCC	GCCTTCCTGA	GCGGCGAGCA

3001 GAAAAAGGCC ATCGTGGACC TGCTGTTCAA GACCAACCGG AAAGTGACCG TGAAGCAGCT
3061 GAAAGAGGAC TACTTCAAGA AAATCGAGTG CTTCGACTCC GTGGAAATCT CCGGCGTGGA
3121 AGATCGGTTC AACGCCTCCC TGGGCACATA CCACGATCTG CTGAAAATTA TCAAGGACAA
3181 GGACTTCCTG GACAATGAGG AAAACGAGGA CATTCTGGAA GATATCGTGC TGACCCTGAC
3241 ACTGTTTGAG GACAGAGAGA TGATCGAGGA ACGGCTGAAA ACCTATGCCC ACCTGTTCGA
3301 CGACAAAGTG ATGAAGCAGC TGAAGCGGCG GAGATACACC GGCTGGGGCA GGCTGAGCCG
3361 GAAGCTGATC AACGGCATCC GGGACAAGCA GTCCGGCAAG ACAATCCTGG ATTTCCTGAA
3421 GTCCGACGGC TTCGCCAACA GAAACTTCAT GCAGCTGATC CACGACGACA GCCTGACCTT
3481 TAAAGAGGAC ATCCAGAAAG CCCAGGTGTC CGGCCAGGGC GATAGCCTGC ACGAGCACAT
3541 TGCCAATCTG GCCGGCAGCC CCGCCATTAA GAAGGGCATC CTGCAGACAG TGAAGGTGGT
3601 GGACGAGCTC GTGAAAGTGA TGGGCCGGCA CAAGCCCGAG AACATCGTGA TCGAAATGGC
3661 CAGAGAGAAC CAGACCACCC AGAAGGGACA GAAGAACAGC CGCGAGAGAA TGAAGCGGAT
3721 CGAAGAGGGC ATCAAAGAGC TGGGCAGCCA GATCCTGAAA GAACACCCCG TGGAAAACAC
3781 CCAGCTGCAG AACGAGAAGC TGTACCTGTA CTACCTGCAG AATGGGCGGG ATATGTACGT
3841 GGACCAGGAA CTGGACATCA ACCGGCTGTC CGACTACGAT GTGGACCATA TCGTGCCTCA
3901 GAGCTTTCTG AAGGACGACT CCATCGACAA CAAGGTGCTG ACCAGAAGCG ACAAGAACCG
3961 GGGCAAGAGC GACAACGTGC CCTCCGAAGA GGTCGTGAAG AAGATGAAGA ACTACTGGCG
4021 GCAGCTGCTG AACGCCAAGC TGATTACCCA GAGAAAGTTC GACAATCTGA CCAAGGCCGA
4081 GAGAGGCGGC CTGAGCGAAC TGGATAAGGC CGGCTTCATC AAGAGACAGC TGGTGGAAAC
4141 CCGGCAGATC ACAAAGCACG TGGCACAGAT CCTGGACTCC CGGATGAACA CTAAGTACGA
4201 CGAGAATGAC AAGCTGATCC GGGAAGTGAA AGTGATCACC CTGAAGTCCA AGCTGGTGTC
4261 CGATTTCCGG AAGGATTTCC AGTTTTACAA AGTGCGCGAG ATCAACAACT ACCACCACGC
4321 CCACGACGCC TACCTGAACG CCGTCGTGGG AACCGCCCTG ATCAAAAAGT ACCCTAAGCT
4381 GGAAAGCGAG TTCGTGTACG GCGACTACAA GGTGTACGAC GTGCGGAAGA TGATCGCCAA
4441 GAGCGAGCAG GAAATCGGCA AGGCTACCGC CAAGTACTTC TTCTACAGCA ACATCATGAA
4501 CTTTTTCAAG ACCGAGATTA CCCTGGCCAA CGGCGAGATC CGGAAGCGGC CTCTGATCGA
4561 GACAAACGGC GAAACCGGGG AGATCGTGTG GGATAAGGGC CGGGATTTTG CCACCGTGCG
4621 GAAAGTGCTG AGCATGCCCC AAGTGAATAT CGTGAAAAAG ACCGAGGTGC AGACAGGCGG
4681 CTTCAGCAAA GAGTCTATCC TGCCCAAGAG GAACAGCGAT AAGCTGATCG CCAGAAAGAA
4741 GGACTGGGAC CCTAAGAAGT ACGGCGGCTT CGACAGCCCC ACCGTGGCCT ATTCTGTGCT
4801 GGTGGTGGCC AAAGTGGAAA AGGGCAAGTC CAAGAAACTG AAGAGTGTGA AAGAGCTGCT
4861 GGGGATCACC ATCATGGAAA GAAGCAGCTT CGAGAAGAAT CCCATCGACT TTCTGGAAGC
4921 CAAGGGCTAC AAAGAAGTGA AAAAGGACCT GATCATCAAG CTGCCTAAGT ACTCCCTGTT
4981 CGAGCTGGAA AACGGCCGGA AGAGAATGCT GGCCTCTGCC GGCGAACTGC AGAAGGGAAA
5041 CGAACTGGCC CTGCCCTCCA AATATGTGAA CTTCCTGTAC CTGGCCAGCC ACTATGAGAA
5101 GCTGAAGGGC TCCCCCGAGG ATAATGAGCA GAAACAGCTG TTTGTGGAAC AGCACAAGCA
5161 CTACCTGGAC GAGATCATCG AGCAGATCAG CGAGTTCTCC AAGAGAGTGA TCCTGGCCGA
5221 CGCTAATCTG GACAAAGTGC TGTCCGCCTA CAACAAGCAC CGGGATAAGC CCATCAGAGA
5281 GCAGGCCGAG AATATCATCC ACCTGTTTAC CCTGACCAAT CTGGGAGCCC CTGCCGCCTT
5341 CAAGTACTTT GACACCACCA TCGACCGGAA GAGGTACACC AGCACCAAAG AGGTGCTGGA
5401 CGCCACCCTG ATCCACCAGA GCATCACCGG CCTGTACGAG ACACGGATCG ACCTGTCTCA
5461 GCTGGGAGGC GAC**AAAAGGC** **CGGCGGCCAC** **GAAAAAGGCC** **GGCCAGGCAA** **AAAAGAAAAA**
5521 **G**TAAGAATTC CTAGAGCTCG CTGATCAGCC TCGACTGTGC CTTCTAGTTG CCAGCCATCT
5581 GTTGTTTGCC CCTCCCCCGT GCCTTCCTTG ACCCTGGAAG GTGCCACTCC CACTGTCCTT
5641 TCCTAATAAA ATGAGGAAAT TGCATCGCAT TGTCTGAGTA GGTGTCATTC TATTCTGGGG
5701 GGTGGGGTGG GGCAGGACAG CAAGGGGGAG GATTGGGAAG AGAATAGCAG GCATGCTGGG
5761 GAGCGGCCGC AGGAACCCCT AGTGATGGAG TTGGCCACTC CCTCTCTGCG CGCTCGCTCG
5821 CTCACTGAGG CCGGGCGACC AAAGGTCGCC CGACGCCCGG GCTTTGCCCG GGCGGCCTCA
5881 GTGAGCGAGC GAGCGCGCAG CTGCCTGCAG GGGCGCCTGA TGCGGTATTT TCTCCTTACG
5941 CATCTGTGCG GTATTTCACA CCGCATACGT CAAAGCAACC ATAGTACGCG CCCTGTAGCG
6001 GCGCATTAAG CGCGGCGGGT GTGGTGGTTA CGCGCAGCGT GACCGCTACA CTTGCCAGCG
6061 CCTTAGCGCC CGCTCCTTTC GCTTTCTTCC CTTCCTTTCT CGCCACGTTC GCCGGCTTTC

6121 CCCGTCAAGC TCTAAATCGG GGGCTCCCTT TAGGGTTCCG ATTTAGTGCT TTACGGCACC
6181 TCGACCCCAA AAAACTTGAT TTGGGTGATG GTTCACGTAG TGGGCCATCG CCCTGATAGA
6241 CGGTTTTTCG CCCTTTGACG TTGGAGTCCA CGTTCTTTAA TAGTGGACTC TTGTTCCAAA
6301 CTGGAACAAC ACTCAACTCT ATCTCGGGCT ATTCTTTTGA TTTATAAGGG ATTTTGCCGA
6361 TTTCGGTCTA TTGGTTAAAA AATGAGCTGA TTTAACAAAA ATTTAACGCG AATTTTAACA
6421 AAATATTAAC GTTTACAATT TTATGGTGCA CTCTCAGTAC AATCTGCTCT GATGCCGCAT
6481 AGTTAAGCCA GCCCCGACAC CCGCCAACAC CCGCTGACGC GCCCTGACGG GCTTGTCTGC
6541 TCCCGGCATC CGCTTACAGA CAAGCTGTGA CCGTCTCCGG GAGCTGCATG TGTCAGAGGT
6601 TTTCACCGTC ATCACCGAAA CGCGCGAGAC GAAAGGGCCT CGTGATACGC CTATTTTTAT
6661 AGGTTAATGT CATGATAATA ATGGTTTCTT AGACGTCAGG TGGCACTTTT CGGGGAAATG
6721 TGCGCGGAAC CCCTATTTGT TTATTTTTCT AAATACATTC AAATATGTAT CCGCTCATGA
6781 GACAATAACC CTGATAAATG CTTCAATAAT ATTGAAAAAG GAAGAGT**ATG AGTATTCAAC**
6841 **ATTTCCGTGT CGCCCTTATT CCCTTTTTTG CGGCATTTTG CCTTCCTGTT TTTGCTCACC**
6901 **CAGAAACGCT GGTGAAAGTA AAAGATGCTG AAGATCAGTT GGGTGCACGA GTGGGTTACA**
6961 **TCGAACTGGA TCTCAACAGC GGTAAGATCC TTGAGAGTTT TCGCCCCGAA GAACGTTTTC**
7021 **CAATGATGAG CACTTTTAAA GTTCTGCTAT GTGGCGCGGT ATTATCCCGT ATTGACGCCG**
7081 **GGCAAGAGCA ACTCGGTCGC CGCATACACT ATTCTCAGAA TGACTTGGTT GAGTACTCAC**
7141 **CAGTCACAGA AAAGCATCTT ACGGATGGCA TGACAGTAAG AGAATTATGC AGTGCTGCCA**
7201 **TAACCATGAG TGATAACACT GCGGCCAACT TACTTCTGAC AACGATCGGA GGACCGAAGG**
7261 **AGCTAACCGC TTTTTTGCAC AACATGGGGG ATCATGTAAC TCGCCTTGAT CGTTGGGAAC**
7321 **CGGAGCTGAA TGAAGCCATA CCAAACGACG AGCGTGACAC CACGATGCCT GTAGCAATGG**
7381 **CAACAACGTT GCGCAAACTA TTAACTGGCG AACTACTTAC TCTAGCTTCC CGGCAACAAT**
7441 **TAATAGACTG GATGGAGGCG GATAAAGTTG CAGGACCACT TCTGCGCTCG GCCCTTCCGG**
7501 **CTGGCTGGTT TATTGCTGAT AAATCTGGAG CCGGTGAGCG TGGAAGCCGC GGTATCATTG**
7561 **CAGCACTGGG GCCAGATGGT AAGCCCTCCC GTATCGTAGT TATCTACACG ACGGGGAGTC**
7621 **AGGCAACTAT GGATGAACGA AATAGACAGA TCGCTGAGAT AGGTGCCTCA CTGATTAAGC**
7681 **ATTGGTAA**CT GTCAGACCAA GTTTACTCAT ATATACTTTA GATTGATTTA AAACTTCATT
7741 TTTAATTTAA AAGGATCTAG GTGAAGATCC TTTTTGATAA TCTCATGACC AAAATCCCTT
7801 AACGTGAGTT TTCGTTCCAC TGAGCGTCAG ACCCCGTAGA AAAGATCAAA GGATCTTCTT
7861 GAGATCCTTT TTTTCTGCGC GTAATCTGCT GCTTGCAAAC AAAAAAACCA CCGCTACCAG
7921 CGGTGGTTTG TTTGCCGGAT CAAGAGCTAC CAACTCTTTT TCCGAAGGTA ACTGGCTTCA
7981 GCAGAGCGCA GATACCAAAT ACTGTTCTTC TAGTGTAGCC GTAGTTAGGC CACCACTTCA
8041 AGAACTCTGT AGCACCGCCT ACATACCTCG CTCTGCTAAT CCTGTTACCA GTGGCTGCTG
8101 CCAGTGGCGA TAAGTCGTGT CTTACCGGGT TGGACTCAAG ACGATAGTTA CCGGATAAGG
8161 CGCAGCGGTC GGGCTGAACG GGGGGTTCGT GCACACAGCC CAGCTTGGAG CGAACGACCT
8221 ACACCGAACT GAGATACCTA CAGCGTGAGC TATGAGAAAG CGCCACGCTT CCCGAAGGGA
8281 GAAAGGCGGA CAGGTATCCG GTAAGCGGCA GGGTCGGAAC AGGAGAGCGC ACGAGGGAGC
8341 TTCCAGGGGG AAACGCCTGG TATCTTTATA GTCCTGTCGG GTTTCGCCAC CTCTGACTTG
8401 AGCGTCGATT TTTGTGATGC TCGTCAGGGG GGCGGAGCCT ATGGAAAAAC GCCAGCAACG
8461 CGGCCTTTTT ACGGTTCCTG GCCTTTTGCT GGCCTTTTGC TCACATGT

Qualification by Sequencing

Primer Name	Primer Sequence	Strand
**gRNA-F1**	CGGAGCCTATGGAAAAACGC	Forward
**hCas9-R1**	CAGATCCGGTTCTTCCGTCT	Reverse

Qualification by Restriction Enzyme Digestion

Cutters	Locations	Fragments(bp)
**ScaI**	2919, 4476, 4971, 5346, 7135	1557, 495, 375, 1789, 4292

Supplementary Data File S2:

Real time PCR conditions used for determination of relative expression level of *Tcte1* gene in mouse knockout animals.

Real-time PCR:

The cDNA was synthesized from 3 µg of total RNA using iScript™ Reverse Transcription Supermix (Bio-Rad Laboratories) in 20 µL reaction volume in a thermocycler PTC-200 (MJ Research).

The Real-time PCR was performed using specific primers for *Tcte1* (forward primer: 5'-GAGCGAGCCAGAGAAGGAC-3'; reverse primer: 5'- CGGTAGGTGAAGAGAAAGAGGT-3') with SsoAdvanced ™ SYBR® Green supermix (Bio-Rad Laboratories).

The threshold cycle (Ct) values of each studied transcript was analyzed with CFX384 Touch™ Real-time PCR detection system (Bio-Rad) using standard cycling parameters. All samples and standard curve were run in duplicates. The relative expression level of each studied transcript was normalized with reference to three housekeeping genes according to GeNorm software.

Supplementary Data File S3: Genotyping protocol for Tcte1 knockout model.

Genotyping Protocol for NTMCK-160509-AKM-01_Mouse Tcte1 Project

3.3. PCR primers:

3.3a. Identification of wildtype and positives:

Mouse Tcte1-F: CGTTTTAAGAATGTATTGAGGGTTGGG

Mouse Tcte1-R: CTCCGTAGGCTCCTGCCAATATG

Product Size: WT: 1915 bp; MT: ~630 bp, delete~1280 bp

Annealing Temp: 59℃

Purify and extract the PCR products from gel and perform sequencing for analysis.

Forward Primer for sequencing

Mouse Tcte1-SEQ-F: CGTTTTAAGAATGTATTGAGGGTTGGG

PCR assay:

Table PCR mixture

	×1	×10	×20	×30	×40	×50	×60
ddH_2O	21.85	218.5	437	655.5	874	1092.5	1311
dNTP Mix	1.5	15	30	45	60	75	90
10×buffer	3	30	60	90	120	150	180
HS Taq	0.15	1.5	3	4.5	6	7.5	9
Mouse Tcte1-F	1	10	20	30	40	50	60
Mouse Tcte1-R	1	10	20	30	40	50	60
DNA Template	1.5	15	30	45	60	75	90
Total	30	300	600	900	1200	1500	1800

PCR reaction conditions

A. Initial denaturation 94℃ 5 min

B. Denaturation 94℃ 30 s

C. Annealing 59 ℃ 30 s

D. Extension 72 ℃ 2 min

(B–D: 35cycles)

E. Additional extension 72 ℃ 5 min

F. Keeping temperature 12 ℃

PCR result analysis:

① One band: 630 bp; heterozygotes and homozygotes;

② One band: 1915 bp or 0 bp; wildtype.

3.3b. Identification of heterozygotes and homozygotes:

(To confirm whether the positive founders were homozygotes or not, one primer located inside the deletion sequence and other located outside.)

Mouse Tcte1-Wt/He-F: AGCTTGCCACACCCTCAAGGTACTA

Mouse Tcte1-R: CTCCGTAGGCTCCTGCCAATATG

Product Size: 658 bp or 0 bp

Annealing Temp: 59℃

PCR assay:

Table PCR mixture

	×1	×10	×20	×30	×40	×50	×60
ddH_2O	21.85	218.5	437	655.5	874	1092.5	1311
dNTP Mix	1.5	15	30	45	60	75	90
10×buffer	3	30	60	90	120	150	180
HS Taq	0.15	1.5	3	4.5	6	7.5	9
Mouse Tcte1-Wt/He-F	1	10	20	30	40	50	60

Mouse Tcte1-R	1	10	20	30	40	50	60
DNA Template	1.5	15	30	45	60	75	90
Total	30	300	600	900	1200	1500	1800

PCR reaction conditions

A. Initial denaturation	94℃	5 min	
B. Denaturation	94℃	30 s	35cycles
C. Annealing	59 ℃	30 s	
D. Extension	72 ℃	30 s	
E. Additional extension	72 ℃	5 min	
F. Keeping temperature	12 ℃		

PCR result analysis:

① One band: 658 bp; heterozygotes;

② No band: 0 bp; homozygotes.

Supplementary Data File S4:

Detailed parameters of mouse sperm morphology. Statistically significant values: $p < 0.05$; ns – statistically not significant ($p \geq 0.05$). For samples with normal distribution (positive Shapiro-Wilk normality test), t-test with Welch's correction was applied; for samples without normal distribution (midpiece and tail defects), Kolmogorov-Smirnoff test was applied (GraphPad Prism v. 7.0e). n=200 spermatozoa were analyzed per each mouse; for WT and $Tcte1^{-/-}$ n=5 animals were evaluated, while for $Tcte1^{-/+}$ the number of animals was n=7. Spermatozoa stained with the classic Papanicolau staining procedure. Bright field microscope: Leica DM5500, magnification 630x (with immersion), software: CytoVision.

sperm morphology		genotype	% [mean ± SD]	p value		
				$Tcte1^{-/-}$ vs. $Tcte^{+/-}$	$Tcte1^{-/-}$ vs. WT	$Tcte1^{+/-}$ vs. WT
**normal**		$Tcte1^{-/-}$	35.41 ± 9.10	< 0.0001	0.0009	ns
		$Tcte^{+/-}$	71.94 ± 8.97			
		WT	70.81 ± 11.78			
**abnormal**	**head defects:**					
	sum	$Tcte1^{-/-}$	95.08 ± 2.49	0.0163	ns	ns
		$Tcte^{+/-}$	65.83 ± 23.60			
		WT	84.33 ± 11.89			
	hook less	$Tcte1^{-/-}$	1.30 ± 1.72	ns	ns	ns
		$Tcte^{+/-}$	3.07 ± 4.21			
		WT	2.81 ± 2.68			
	big size	$Tcte1^{-/-}$	1.65 ± 1.22	ns	ns	ns
		$Tcte^{+/-}$	3.03 ± 2.95			
		WT	0.71 ± 1.60			
	small size	$Tcte1^{-/-}$	0.36 ± 0.81	ns	ns	ns
		$Tcte^{+/-}$	0			
		WT	0			
	amorphous	$Tcte1^{-/-}$	26.11 ± 6.35	ns	0.0011	0.0314
		$Tcte^{+/-}$	33.72 ± 13.00			
		WT	48.88 ± 7.83			
	banana shaped	$Tcte1^{-/-}$	13.11 ± 9.30	ns	ns	ns
		$Tcte^{+/-}$	6.85 ± 7.16			
		WT	8.59 ± 5.05			
	decapitated	$Tcte1^{-/-}$	52.55 ± 14.24	0.0039	0.0073	ns
		$Tcte^{+/-}$	19.16 ± 9.33			
		WT	23.33 ± 10.79			
	**midpiece defects:**					
	sum	$Tcte1^{-/-}$	0.38 ± 0.55	0.0417	0.0079	ns
		$Tcte^{+/-}$	9.71 ± 12.64			
		WT	8.20 ± 11.42			
	thin	$Tcte1^{-/-}$	0.38 ± 0.55	ns	ns	ns
		$Tcte^{+/-}$	0			
		WT	0.87 ± 1.27			
	folded	$Tcte1^{-/-}$	0	0.0366	0.0476	ns
		$Tcte^{+/-}$	9.71 ± 12.64			
		WT	7.33 ± 11.98			
	**tail defects:**					
	sum	$Tcte1^{-/-}$	4.54 ± 2.79	0.0006	ns	0.0006
		$Tcte^{+/-}$	24.46 ± 24.65			
		WT	7.47 ± 4.06			
	short	$Tcte1^{-/-}$	0	ns	ns	ns
		$Tcte^{+/-}$	0.18 ± 0.47			
		WT	0.26 ± 0.59			
	folded	$Tcte1^{-/-}$	0	ns	ns	ns
		$Tcte^{+/-}$	0.18 ± 0.47			
		WT	1.93 ± 3.23			
	coiled	$Tcte1^{-/-}$	4.54 ± 2.79	0.0008	ns	0.0005
		$Tcte^{+/-}$	23.94 ± 25.12			
		WT	5.33 ± 2.43			
	double	$Tcte1^{-/-}$	0	ns	ns	ns
		$Tcte^{+/-}$	0.17 ± 0.45			
		WT	0			

NORMAL SPERMATOZOA

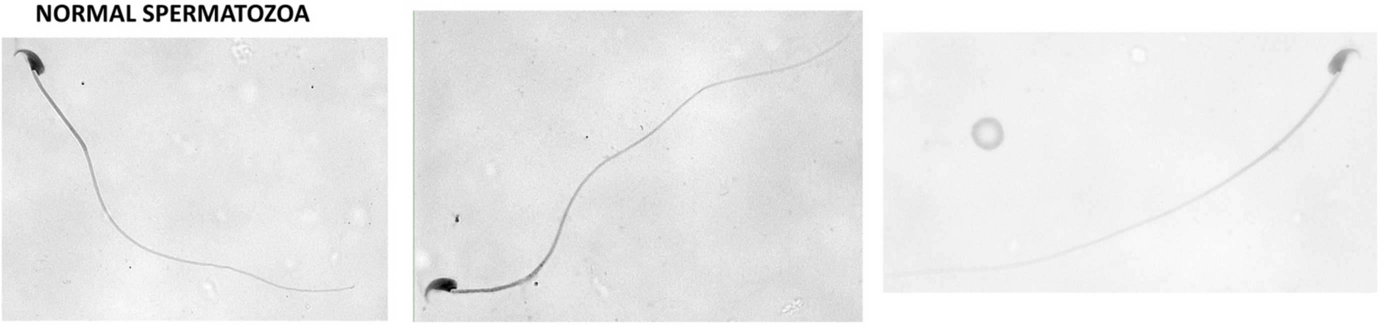

ABNORMAL SPERM MORPHOLOGY

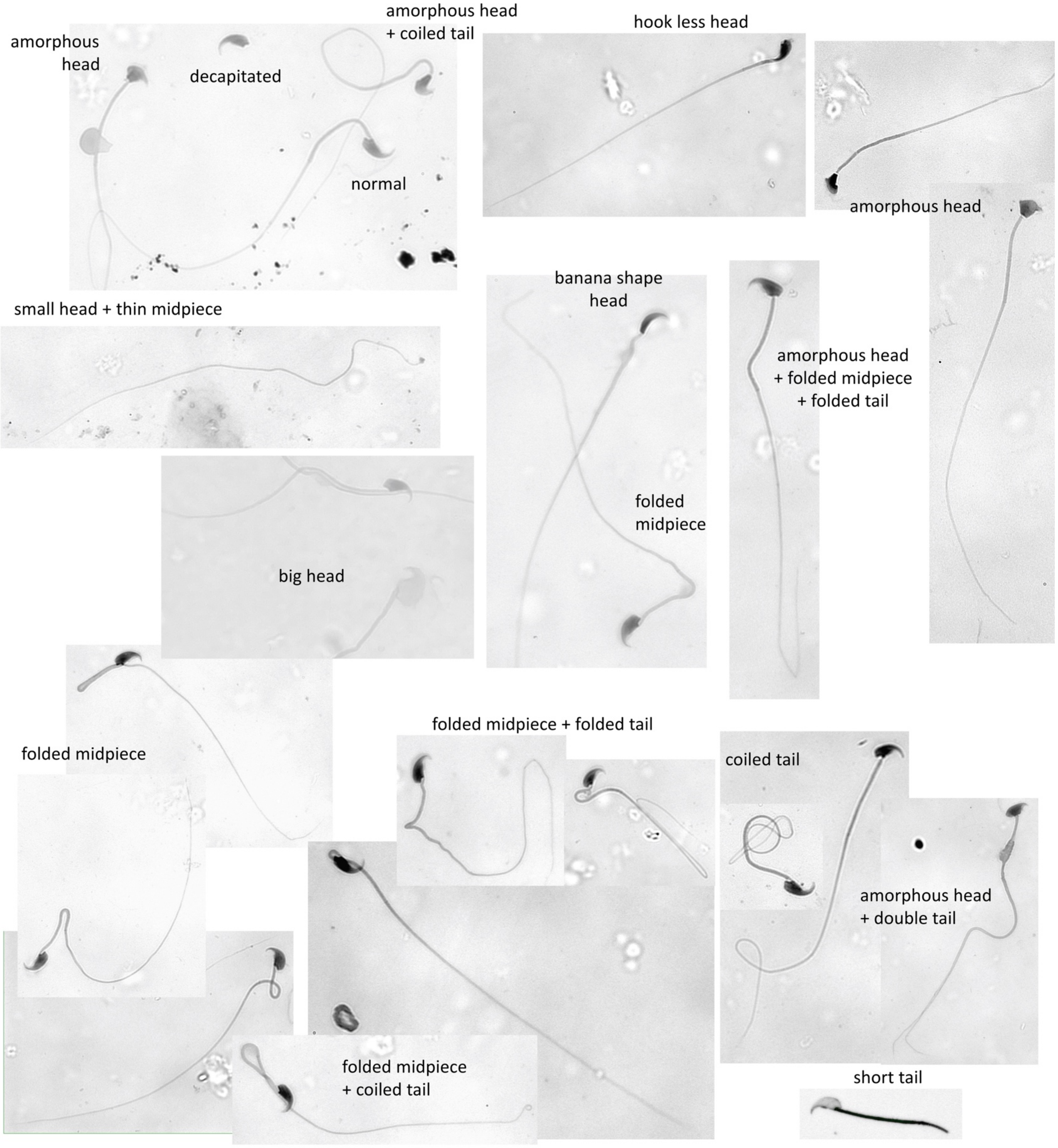

Supplement: hoae020_Supplementary_Data [file hoae020_supplementary_data.zip › Supplementary Data Files S1-S4.pdf]
